# First Organocatalytic Asymmetric Synthesis of 1-Benzamido-1,4-Dihydropyridine Derivatives

**DOI:** 10.3390/molecules23102692

**Published:** 2018-10-19

**Authors:** Fernando Auria-Luna, Eugenia Marqués-López, Raquel P. Herrera

**Affiliations:** Laboratorio de Organocatálisis Asimétrica, Departamento de Química Orgánica, Instituto de Síntesis Química y Catálisis Homogénea (ISQCH) CSIC-Universidad de Zaragoza, C/Pedro Cerbuna 12, E-50009 Zaragoza, Spain; ferauria@unizar.es (F.A.-L.); mmaamarq@unizar.es (E.M.-L.)

**Keywords:** chiral base, 1,4-dihydropyridine, enantioselective, hydrazone, organocatalysis

## Abstract

Preliminary results concerning the first asymmetric synthesis of highly functionalized 1-benzamido-1,4-dihydropyridine derivatives via the reaction of hydrazones with alkylidenemalononitriles in the presence of β-isocupreidine catalyst are reported. The moderate, but promising, enantioselectivity observed (40–54% ee), opens the door to a new area of research for the asymmetric construction of new chiral 1,4-dihydropyridine derivatives, whose enantioselective catalytic preparation are still very limited. Moreover, the use of hydrazones for the enantioselective construction of chiral 1,4-dihydropyridines has been overlooked in the literature so far. Therefore, our research represents a pivotal example in this field which remains still unexplored.

## 1. Introduction

**Figure 1:**
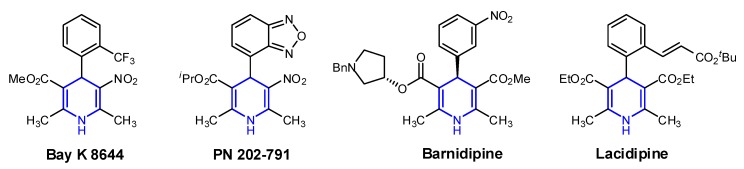
1,4-Dihydropyridine based drugs as calcium channel blockers.

1,4-Dihydropyridine (1,4-DHP) [1,2,3,4,5] ring is a challenging structural core in organic chemistry due to its biological properties [6,7,8,9], especially as calcium channel blockers (Figure 1) [10,11]. It is noteworthy that its range of application has been recently extended to other affections such as antioxidant, antidiabetic and antitumor agents [12]. 

Therefore, the potential of 1,4-dihydropyridines as valuable building blocks in organic synthesis has attracted the attention of many scientists [13,14]. Moreover, it has been found that the stereochemistry at C-4 can be related with both qualitative and quantitative differences in their biological activity. Thus, the control of the stereoselectivity in this chiral center has become an inspiring task of research and, therefore, there is a growing interest for the development of new enantioselective methods. However, there are only a few examples of organocatalytic enantioselective syntheses to obtain these compounds [15,16]. 

**Scheme 1:**
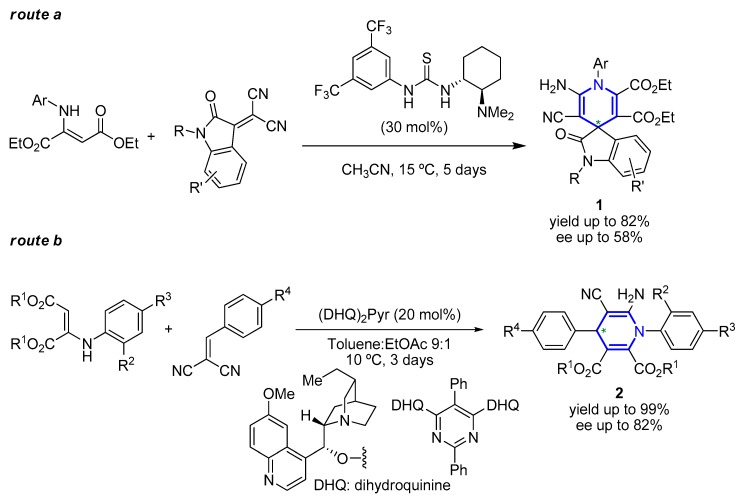
Synthesis of highly substituted chiral 1,4-DHPs **1** (route a) [17]) and **2** (route b) [16]).

Recently, we have successfully contributed to this field with two pioneering works (Scheme 1) [16,17]. In the first example, we used Takemoto’s thiourea to synthesize chiral 2-oxospiro-[indole-3,4′-(1′,4′-dihydropyridine)] derivatives **1** with good reactivity and promising enantioselectivities (Scheme 1, route a) [17]. The most recent work has allowed us to obtain for the first time a family of substituted chiral 1,4-dihydropyridines **2** with very good results, using a bis-cinchona derivative as organocatalyst (Scheme 1, route b) [16].

**Scheme 2:**
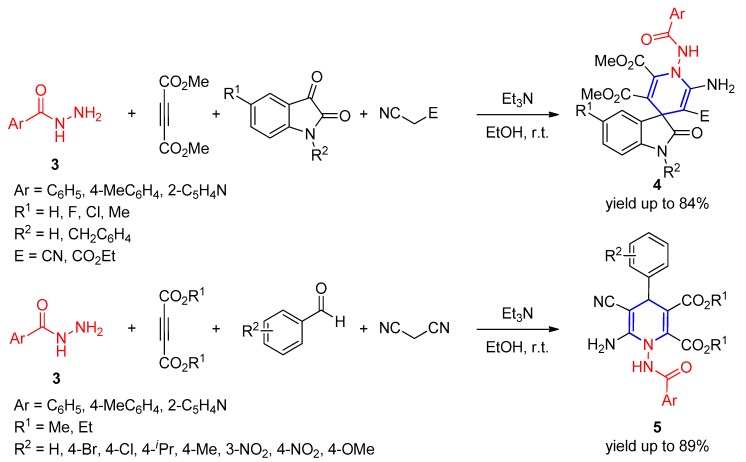
Synthesis of highly substituted benzamidospiro[indoline-3,4′-pyridines] **4** [18] and benzamido-1,4-dihydropyridines **5** [19].

Related to our work reported herein, we must remark that Yan and co-workers previously published two interesting racemic versions using benzohydrazides **3**, instead of anilines, in two concomitant multicomponent processes to obtain 1-benzamidospiro[indoline-3,4′-pyridines] **4** [18] or benzamido-1,4-dihydropyridines **5** [19] (Scheme 2).

Based on this idea, we envisioned that the use of chiral amine-based catalysts could provide the first asymmetric version of a related reaction. Moreover, to the best of our knowledge, the use of hydrazones for the enantioselective construction of 1,4-dihydropyridines has been overlooked in the literature so far. It is worth noting that the development of new asymmetric strategies to obtain enantioenriched 1,4-DHPs is still desirable since there are only scarce examples in this field of research [15,16]. 

## 2. Results and Discussion

### 2.1. Synthesis of Starting Materials: Hydrazones ***7*** and Alkylidenemalononitriles ***9***

**Scheme 3:**
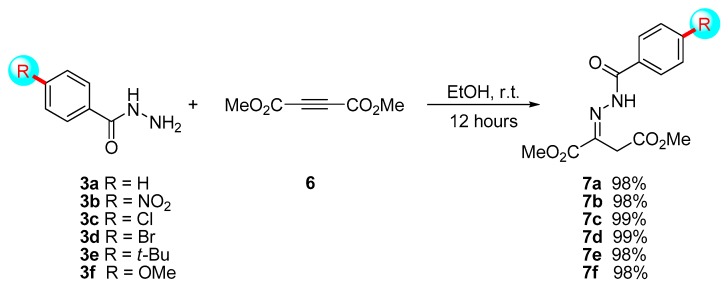
Preparation of hydrazones **7a**–**f**.

On the base of our recent works [16,17], we hypothesized that the enamine generated from hydrazones **7** could provide the same reactivity as reported in Scheme 1 and Scheme 2. With this aim in mind, we firstly synthesized six different hydrazones **7a**–**f** as described below in Scheme 3. 

Hydrazones **7** were prepared after 12 h at room temperature with quantitative yields from differently substituted benzohydrazides **3**, bearing different electronic properties in the aromatic ring (electron-withdrawing or electron-donating groups or none of them) (Scheme 3). In contrast to our previous works [16,17], the reaction between alkynyl **6** and hydrazide **3** leads to pure hydrazones **7** instead of the corresponding enamine (shown in Scheme 1), as previously obtained by us in the reaction between alkynyl **6** and an aniline. 

**Scheme 4:**
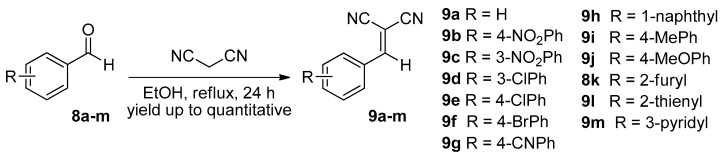
Synthesis of alkylidenemalononitriles **9a**–**m**.

The preparation of diverse alkylidenemalononitriles **9a**–**m** was further performed, following the general procedure described in Scheme 4, in quantitative yields [17].

### 2.2. Screening of the Reaction Conditions

**Scheme 5:**
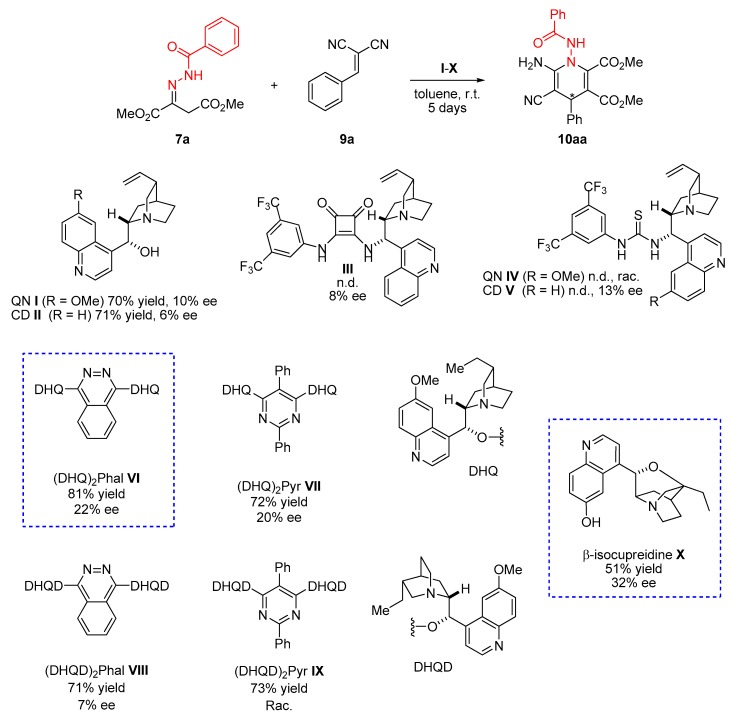
Chiral organocatalysts **I-X** tested to synthesize chiral 1,4-DHPs **10aa**. Rac. = racemic mixture. N.d. = not determined.

To carry out the synthesis of the first chiral 1-benzamido-1,4-dihydropyridine derivatives **10** [16,17], we foresaw that a chiral organic base catalyst could promote this reaction, starting directly from the preformed intermediates: hydrazones **7** and malononitriles **9**, giving rise to the desired final benzamido-1,4-dihydropyridines **10**. To explore the viability of this hypothesis, the efficiency of different chiral organocatalysts **I-X**, with a base moiety in their structure, was initially studied in a model reaction between hydrazone **7a** and malononitrile **9a** (Scheme 5). 

As reported in Scheme 5, the most promising value of enantioselectivity was achieved with β-isocupreidine **X** (32% ee), while the best reactivity was found with (DHQ)_2_Phal **VI** (81% yield). These results encouraged us to continue with both catalysts **VI** and **X** in the subsequent screening of different parameters to optimize this process (Table 1).

As shown in Table 1, parameters such as solvent, concentration of the reaction, amount of each reagent and catalyst were analyzed. In general, catalyst **VI** still provided better reactivity in some reactions media, such as MeCN, AcOEt, CH_2_Cl_2_ or MeOH (entries 1, 3, 5 and 13), in comparison with catalyst **X** (entries 2, 4, 6 and 14). However, better enantioselectivities were found with catalyst **X** in almost all solvents (except in CH_2_Cl_2_, entry 6). The best compromise in terms of reactivity and enantioselectivity was achieved in THF (entries 12, 15–20) using catalyst **X**. Concentration of the reaction medium led to a slight decrease in the enantioselectivity of the process with a smooth increase of the reactivity (entry 17). The opposite behavior is true when the reaction was diluted (entry 18). Variations in the amount of catalyst did not provide an appreciable improvement of the process (entries 19 and 20). Increasing the amount of reagents **7a** or **9a** gave rise to similar results in both cases (entries 15 and 16), although the use of 1.5 equivalent of **9a** led to slightly better reactivity (entry 15). Therefore, we continue to the ensuing exploration of the scope of the reaction with these conditions (entry 15), as they are the best ones at this stage.

### 2.3. Scope of the Reaction

**Scheme 6:**
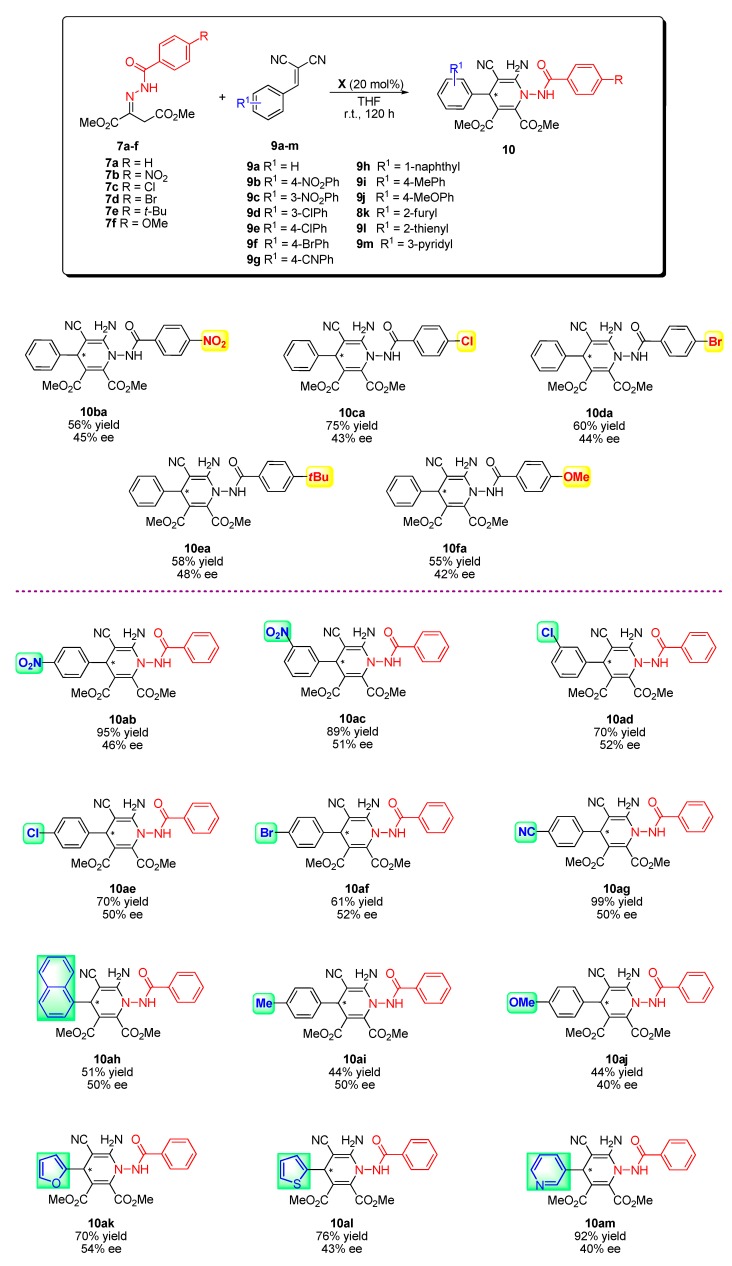
Scope of the reaction to obtain 1-benzamido-1,4-dihydropyridine derivatives **10**.

Once the reaction conditions were optimized, a series of 1-benzamido-1,4-dihydropyridine derivatives **10** were synthesized as shown in Scheme 6.

The final products **10** were isolated with high yields (up to 99%) and with moderate but promising enantioselectivities (up to 54% ee) since this work represents the first chiral version to obtain enantioenriched 1-benzamido-1,4-dihydropyridines. The results suggest a dependence of the reactivity of the process on the electronic properties of the aromatic ring in the alkylidenemalononitriles **9**, since electron-withdrawing groups (4-NO_2_, 3-NO_2_, 3-Cl, 4-Cl, 4-Br or 4-CN) or heteroaromatic rings (2-furyl, 2-thienyl, 3-pyridyl) afforded better results in comparison with electrondonating substituents (4-Me and 4-MeO) or in the absence of substituents (1-naphthyl or phenyl). On the other hand, the enantioselectivity of the process does not have a clear correlation with the electronic properties of the aromatic ring in the alkylidenemalononitriles **9**. Interestingly, we were able to obtain the desired 1-benzamido-1,4-dihydropyridines **10** changing the substituent in the aromatic ring of the hydrazone (**7b**–**f**), achieving more similar results in terms of enantioselectivity and reactivity than those obtained with hydrazone **7a**.

Based on our previously reported works for the obtainment of chiral 1,4-dihydropyridine derivatives [16,17], in the field of cinchona alkaloids and in our own experience using this kind of organocatalysts [20,21,22], we think that the same mechanism could be operating in this case [16,17]. Thus, a first Michael addition reaction between the enamine generated in situ from hydrazones **7** and the alkylidenemalononitriles **9**, followed by an intramolecular nucleophilic cyclization and a final tautomerization would yield 1-benzamido-1,4-dihydropyridines **10**. However, in order to really know if β-isocupreidine **X** is acting as a bifunctional catalyst [23,24,25] and to better understand the role of this structure, more studies are ongoing in our lab.

## 3. Materials and Methods

### 3.1. General Experimental Methods

Purification of reaction products was carried out by column chromatography using silical-gel (0.063–0.200 mm). Analytical thin layer chromatography was performed on 0.25 mm silical gel 60-F plates. ESI (Zaragoza, Spain) and MicroTof-Q Bruker mass analyzer (Zaragoza, Spain) were used for HRMS measurements. HPLC was performed on analytical HPLC Waters (Delta 600 Separation Module, 2996 Photodiode Array Detector, Zaragoza, Spain). IR spectra have been registered in a PerkinElmer Spectrum 100 FT-IR Spectrometer (Zaragoza, Spain). The optical rotation measurements were taken on a JASCO Digital polarimeter DIP-370 (Zaragoza, Spain). NMR spectroscopy was conducted using a Bruker AVANCE–II spectrometer (Zaragoza, Spain). ^1^H-NMR spectra were recorded at 300 and 400 MHz; ^13^C-APT-NMR spectra were recorded at 75 and 100 MHz; (min: minor isomer); DMSO-*d*_6_ was used as the deuterated solvent. Chemical shifts were reported in the δ scale relative to residual DMSO (2.50 ppm) for ^1^H-NMR and to the central line of DMSO-*d*_6_ (39.52 ppm) for ^13^C-APT-NMR. Spectral data for many of the synthesized compounds are consistent with values previously reported in the literature: hydrazone **7a** [26]; alkylidenemalononitriles **9a** [27], **9b** [27], **9c** [28], **9d** [28], **9e** [27], **9f** [29], **9g** [30], **9i** [27], **9j** [27], **9k** [28], **9l** [31] and **9m** [31]; and 1,4-dihydropyridines **10ac** [19], **10ae** [19], **10af** [19] and **10ai** [19]. Melting points were recorded on a Gallenkamp MDP350 Variable Heater melting point apparatus without correction. Catalysts **I** [32], **II** [32], **VI** [33], **VII** [34], **VIII** [33], **IX** [35] and **X** [36] were commercially available and **III** [37], **IV** [38] and **V** [38] were synthesized as reported.

### 3.2. General Procedure for the Synthesis of Hydrazones ***7***

To a solution of the corresponding hydrazide **3** (5 mmol) in ethanol (15 mL), dimethyl acetylenedicarboxylate **6** (5 mmol, 615 μL) was slowly added due to the exothermic characteristics of the reaction. The reaction mixture was stirred for 12 h at room temperature. Then, the solvent was evaporated under vacuum, and the reaction crude was purified by filtration, washing the white precipitate with small portions of ethanol (3 × 3 mL) giving rise to the corresponding final adduct **7** (Scheme 3).

*(E)-Dimethyl 2-(2-(benzoyl)hydrazono)succinate* (**7a**) [26]: Following the general procedure, compound **7a** was obtained as a white solid in a 98% yield. 

*(E)-Dimethyl 2-(2-(4-nitrobenzoyl)hydrazono)succinate* (**7b**): Following the general procedure, compound **7b** was obtained as a white solid in a 98% yield. mp 146–148 °C. ^1^H-NMR (300 MHz, DMSO-*d*_6_) δ 3.65 (s, 3H), 3.76 (s, 3H), 3.90 (s, 2H), 8.06 (d, *J* = 8.1 Hz, 2H), 8.35 (d, *J* = 8.5 Hz, 2H), 11.70 (s, 1H). ^13^C-APT-NMR (75 MHz, DMSO-*d*_6_) δ 32.6 (1C), 52.2 (1C), 52.6 (1C), 123.3 (2C), 130.3 (2C), 138.8 (1C), 149.3 (1C), 164.4 (1C), 168.2 (3C). IR (neat) (cm^–1^) ν 3451, 3244, 3113, 1729, 1690, 1671, 1520, 1349, 1241, 1124, 1005, 852, 719. HRMS (ESI+) calcd for C_13_H_13_N_3_NaO_7_ 346.0646; found 346.0649 [M + Na]. 

*(E)-Dimethyl 2-(2-(4-chlorobenzoyl)hydrazono)succinate* (**7c**): Following the general procedure, compound **7c** was obtained as a white solid in a 99% yield. mp 138–140 °C. ^1^H-NMR (300 MHz, DMSO-*d*_6_) δ 3.64 (s, 3H), 3.76 (s, 3H), 3.89 (s, 2H), 7.60 (d, *J* = 8.6 Hz, 2H), 7.87 (d, *J* = 8.6 Hz, 2H), 11.47 (s, 1H). ^13^C-APT-NMR (75 MHz, DMSO-*d*_6_) δ 32.5 (1C), 52.2 (1C), 52.5 (1C), 128.4 (2C), 130.7 (2C), 131.7 (1C), 137.0 (1C), 164.5 (1C), 168.3 (3C). IR (neat) (cm^–1^) ν 3441, 3238, 2954, 1741, 1726, 1694, 1670, 1594, 1536, 1440, 1250, 1146, 1123, 1111, 1089, 1003, 889, 846, 756. HRMS (ESI+) calcd for C_13_H_13_ClN_2_NaO_5_ 335.0405; found 335.0385 [M + Na].

*(E)-Dimethyl 2-(2-(4-bromobenzoyl)hydrazono)succinate* (**7d**): Following the general procedure, compound **7d** was obtained as a white solid in a 99% yield. mp 114–116 °C. ^1^H-NMR (300 MHz, DMSO-*d*_6_) δ 3.64 (s, 3H), 3.76 (s, 3H), 3.89 (s, 2H), 7.77 (q, *J* = 8.4 Hz, 4H), 11.48 (s, 1H). ^13^C-APT-NMR (75 MHz, DMSO-*d*_6_) δ 30.9 (3C), 32.5 (1C), 52.2 (1C), 52.6 (1C), 125.9 (1C), 130.9 (2C), 131.3 (2C), 132.1 (2C), 164.5 (1C), 168.3 (2C). IR (neat) (cm^–1^) ν 3256, 2953, 1734, 1716, 1685, 1591, 1434, 1222, 1201, 1127, 1111, 1009, 889, 838, 752. HRMS (ESI+) calcd for C_13_H_13_BrN_2_NaO_5_ 378.9900; found 378.9903 [M + Na].

*(E)-Dimethyl 2-(2-(4-tert-butylbenzoyl)hydrazono)succinate* (**7e**): Following the general procedure, compound **7e** was obtained as a white solid in a 98% yield. mp 165–167 °C. ^1^H-NMR (300 MHz, DMSO-*d*_6_) δ 1.31(s, 9H), 3.64 (s, 3H), 3.77 (s, 3H), 3.89 (s, 2H), 7.54 (d, *J* = 8.6 Hz, 2H), 7.78 (d, *J* = 8.6 Hz, 2H), 11.33 (s, 1H). ^13^C-APT-NMR (75 MHz, DMSO-*d*_6_) δ 30.9 (3C), 32.4 (1C), 34.8 (1C), 52.1 (1C), 52.5 (1C), 125.1 (2C), 128.6 (2C), 130.3 (1C), 155.2(1C), 164.6 (1C), 168.4 (3C). IR (neat) (cm^–1^) ν 3232, 3201, 2964, 2949, 1741, 1721, 1671, 1609, 1536, 1432, 1246, 1205, 1150, 1126, 1118, 1021, 895, 857, 841, 707. HRMS (ESI+) calcd for C_17_H_22_N_2_NaO_5_ 357.1421; found 357.1419 [M + Na].

*(E)-Dimethyl 2-(2-(4-methoxybenzoyl)hydrazono)succinate* (**7f**): Following the general procedure, compound **7f** was obtained as a white solid in a 98% yield. mp 144–146 °C. ^1^H-NMR (300 MHz, DMSO-*d*_6_) δ 3.64 (s, 3H), 3.76 (s, 3H), 3.84 (s, 3H), 3.89 (s, 2H), 7.06 (d, *J* = 8.9 Hz, 2H), 7.87 (d, *J* = 8.9 Hz, 2H), 11.25 (s, 1H). ^13^C-APT-NMR (75 MHz, DMSO-*d*_6_) δ 32.4 (1C), 52.2 (1C), 52.5 (1C), 55.5 (1C), 113.6 (2C), 124.9 (1C), 130.9 (2C), 162.4 (1C), 164.6 (1C), 168.5 (3C). IR (neat) (cm^–1^) ν 3419, 3223, 3189, 1737, 1715, 1659, 1601, 1541, 1508, 1436, 1319, 1256, 1205, 1170, 1143, 1109, 1027, 996, 889, 849, 762. HRMS (ESI+) calcd for C_14_H_16_N_2_NaO_6_ 331.0901; found 331.0888 [M + Na].

### 3.3. General Procedure for the Synthesis of 1,4-Dihydropyridines ***10***

To a mixture of β-isocupreidine catalyst **X** (20 mol%, 6.21 mg) and the corresponding benzylidenemalononitrile **9** (0.15 mmol) in tetrahydrofuran (0.5 mL), hydrazones **7** (0.1 mmol) were added. The reaction mixture was stirred for 5 days at room temperature. Then, the reaction crude was purified by column chromatography (SiO_2_, *n*-hexane:diethyl ether 20:80 to 0:100), giving rise to the corresponding final chiral adducts **10** (Scheme 6).

*Dimethyl 6-amino-1-benzamido-5-cyano-4-phenyl-1,4-dihydropyridine-2,3-dicarboxylate* (**10aa**): Following the general procedure, compound **10aa** was obtained after 120 h of reaction at room temperature and was purified by column chromatography (*n*-hexane:diethyl ether 20:80 to 0:100), as a white solid in 61% yield (26.37 mg). mp 106–108 °C. The ee of the product was determined to be 50% by HPLC using a Daicel Chiralpak IC column (*n*-hexane/*i*-PrOH = 70:30, flow rate 1 mL min^−1^, λ = 237.7 nm): τ_major_ = 30.4 min; τ_minor_ = 11.1 min. [α]_D_^24^ = −25.4 (*c* = 0.07, MeOH, 50% ee). ^1^H-NMR (400 MHz, DMSO-*d*_6_) δ 3.51 (s, 3H), 3.57 (s, 0.75H), 3.64 (s, 2.25H), 4.36 (s, 0.75H), 4.48 (s, 0.25H), 6.38 (s, 1.5H), 6.46 (s, 0.5H), 7.24 (t, *J* = 7.3 Hz, 1.5H), 7.35 (t, *J* = 7.4 Hz, 2H), 7.45–7.59 (m, 3.5H), 7.63 (t, *J* = 7.1 Hz, 1H), 7.79–7.91 (m, 2H), 11.21 (s, 0.75H), 11.32 (s, 0.25H). ^13^C-APT-NMR (100 MHz, DMSO-*d*_6_) δ 39.2 (1C), 51.8 (1C), 52.8 (1C), 58.6 (1C), 104.6 (1C), 120.9 (1C), 126.8 (2C), 127.7 (1C), 127.9 (2C), 128.3 (2C), 128.5 (2C), 131.3 (1C), 132.5 (1C), 142.6 (1C), 145.7 (1C), 151.1 (1C), 162.4 (1C), 164.7 (1C), 166.7 (1C). IR (neat) (cm^–1^) ν 3420, 3334, 3249, 3219, 2957, 2190, 1736, 1707, 1662, 1590, 1479, 1428, 1225, 1110, 717, 699, 689. HRMS (ESI+) calcd for C_23_H_20_N_4_NaO_5_ 455.1326; found 455.1340 [M + Na].

*Dimethyl 6-amino-5-cyano-1-(4-nitrobenzamido)-4-phenyl-1,4-dihydropyridine-2,3-dicarboxylate* (**10ba**): Following the general procedure, compound **10ba** was obtained after 120 h of reaction at room temperature and was purified by column chromatography (*n*-hexane:diethyl ether 20:80 to 0:100), as a yellow solid in 56% yield (26.82 mg). mp 238–240 °C. The ee of the product was determined to be 45% by HPLC using a Daicel Chiralpak IC column (*n*-hexane/*i*-PrOH = 70:30, flow rate 1 mL min^−1^, λ = 249.6 nm): τ_major_ = 47.0 min; τ_minor_ = 21.4 min. [α]_D_^24^ = −6.6 (*c* = 0.13, MeOH, 45% ee). ^1^H-NMR (400 MHz, DMSO-*d*_6_) δ 3.51 (s, 3H), 3.58 (s, 0.78H), 3.65 (s, 2.22H), 4.37 (s, 0.74H), 4.44 (s, 0.26H), 6.55 (s, 1.48H), 6.60 (s, 0.52H), 7.24 (t, *J* = 7.3 Hz, 1.48H), 7.35 (t, *J* = 7.5 Hz, 2H), 7.52 (d, *J* = 7.2 Hz, 1.52H), 8.04 (d, *J* = 8.8 Hz, 0.52H), 8.10 (d, *J* = 8.8 Hz, 1.48H), 8.38 (d, *J* = 8.8 Hz, 2H), 11.62 (s, 0.74H), 11.68 (s, 0.26H). ^13^C-APT-NMR (100 MHz, DMSO-*d*_6_) δ 39.2 (1C), 51.8 (1C), 52.9 (1C), 58.1 (1C), 104.9 (1C), 120.8 (1C), 123.5 (min), 123.6 (2C), 126.9 (1C), 127.6 (2C), 128.3 (2C), 128.6 (min), 129.4 (min), 129.5 (2C), 137.0 (1C), 142.2 (1C), 145.6 (1C), 149.7 (1C), 151.0 (1C), 162.4 (1C), 164.6 (1C), 165.4 (1C). IR (neat) (cm^–1^) ν 3420, 3331, 3208, 2955, 2923, 2852, 2193, 1722, 1710, 1679, 1660, 1589, 1527, 1430, 1346, 1231, 1117, 1081, 697. HRMS (ESI+) calcd for C_23_H_19_N_5_NaO_7_ 500.1177; found 500.1175 [M + Na].

*Dimethyl 6-amino-1-(4-chlorobenzamido)-5-cyano-4-phenyl-1,4-dihydropyridine-2,3-dicarboxylate* (**10ca**): Following the general procedure, compound **10ca** was obtained after 120 h of reaction at room temperature and was purified by column chromatography (*n*-hexane:diethyl ether 20:80 to 0:100), as a white solid in 75% yield (34.88 mg). mp 143–145 °C. The ee of the product was determined to be 43% by HPLC using a Daicel Chiralpak IC column (*n*-hexane/*i*-PrOH = 70:30, flow rate 1 mL min^−1^, λ = 254.0 nm): τ_major_ = 24.2 min; τ_minor_ = 11.6 min. [α]_D_^24^ = −26.7 (*c* = 0.10, MeOH, 43% ee). ^1^H-NMR (400 MHz, DMSO-*d*_6_) δ 3.49 (s, 0.9H), 3.51 (s, 2.1H), 3.56 (s, 0.9H), 3.63 (s, 2.1H), 4.35 (s, 0.7H), 4.47 (s, 0.3H), 6.46 (s, 1.4H), 6.50 (s, 0.6H), 7.24 (t, *J* = 7 Hz, 1.4H), 7.30-7.38 (m, 2.2H), 7.53 (d, *J* = 7,1 Hz, 1.4H), 7.63 (d, *J* = 8.6 Hz, 2H), 7.83 (d, *J* = 8.6 Hz, 0.6H), 7.88 (d, *J* = 8.6 Hz, 1.4H), 11.33 (s, 0.7H), 11.42 (s, 0.3H). ^13^C-APT-NMR (100 MHz, DMSO-*d*_6_) δ 39.1 (1C), 51.9 (1C), 52.8 (min), 52.9 (1C), 57.2 (min), 58.3 (1C), 104.7 (1C), 120.9 (1C), 121.0 (min), 126.9 (min), 127.0 (1C), 127.7 (2C), 128.4 (2C), 128.5 (min), 128.6 (2C), 129.9 (2C), 130.2 (1C), 130.3 (min), 137.2 (1C), 137.4 (1C), 142.5 (1C), 143.0 (1C), 145.7 (1C), 151.1 (1C), 151.6 (1C), 162.4 (1C), 164.7 (1C), 164.8 (1C), 165.3 (1C), 165.8 (1C). IR (neat) (cm^–1^) ν 3413, 3331, 3251, 3025, 2956, 2192, 1727, 1715, 1664, 1590, 1432, 1335, 1230, 1093, 1014, 698. HRMS (ESI+) calcd for C_23_H_19_ClN_4_NaO_5_ 489.0912; found 489.0913 [M + Na].

*Dimethyl 6-amino-1-(4-bromobenzamido)-5-cyano-4-phenyl-1,4-dihydropyridine-2,3-dicarboxylate* (**10da**): Following the general procedure, compound **10da** was obtained after 120 h of reaction at room temperature and was purified by column chromatography (*n*-hexane:diethyl ether 20:80 to 0:100), as a yellow solid in 60% yield (30.81 mg). mp 134–136 °C. The ee of the product was determined to be 44% by HPLC using a Daicel Chiralpak IC column (*n*-hexane/*i*-PrOH = 70:30, flow rate 1 mL min^−1^, λ = 330.0 nm): τ_major_ = 24.6 min; τ_minor_ = 10.7 min. [α]_D_^24^ = −20.3 (*c* = 0.15, MeOH, 44% ee). ^1^H-NMR (400 MHz, DMSO-*d*_6_) δ 3.49 (s, 0.75H), 3.51 (s, 2.25H), 3.56 (s, 0.75H), 3.63 (s, 2.25H), 4.35 (s, 0.75H), 4.47 (s, 0.25H), 6.45 (s, 1.5 H), 6.50 (s, 0.50H), 7.24 (t, *J* = 7.3 Hz, 1.5H), 7.34 (t, *J* = 7.5 Hz, 2H), 7.53 (d, *J* = 7.1 Hz, 1.5H), 7.73–7.84 (m, 4H), 11.33 (s, 0.75H), 11.42 (s, 0.25H). ^13^C-APT-NMR (100 MHz, DMSO-*d*_6_) δ 39.2 (1C), 51.9 (1C), 52.8 (1C), 58.3 (1C), 104.7 (1C), 120.9 (1C), 126.3 (1C), 126.8 (1C), 126.9 (min), 127.6 (2C), 128.3 (2C), 128.6 (min), 130.0 (2C), 130.5 (min), 131.4 (2C), 131.5 (1C), 142.4 (1C), 145.6 (1C), 151.1 (1C), 162.4 (1C), 164.6 (1C), 165.9 (1C). IR (neat) (cm^–1^) ν 3417, 3331, 3240, 2955, 2190, 1716, 1663, 1588, 1431, 1230, 1079, 1010, 698. HRMS (ESI+) calcd for C_23_H_19_BrN_4_NaO_5_ 533.0418; found 533.0418 [M + Na].

*Dimethyl 6-amino-1-(4-(tert-butyl)benzamido)-5-cyano-4-phenyl-1,4-dihydropyridine-2,3-dicarboxylate* (**10ea**): Following the general procedure, compound **10ea** was obtained after 120 h of reaction at room temperature and was purified by column chromatography (*n*-hexane:diethyl ether 20:80 to 0:100), as a yellow solid in 58% yield (28.33 mg). mp 141–143 °C. The ee of the product was determined to be 48% by HPLC using a Daicel Chiralpak IC column (*n*-hexane/*i*-PrOH = 70:30, flow rate 1 mL min^−1^, λ = 242.5 nm): τ_major_ = 13.2 min; τ_minor_ = 9.1 min. [α]_D_^24^ = −24.7 (*c* = 0.15, MeOH, 48% ee). ^1^H-NMR (400 MHz, DMSO-*d*_6_) δ 1.31 (s, 9H), 3.51 (s, 3H), 3.58 (s, 0.78H), 3.66 (s, 2.22H), 4.36 (s, 0.74H), 4.48 (s, 0.26H), 6.33 (s, 1.48H), 6.38 (s, 0.52H), 7.24 (t, *J* = 7.4 Hz, 1.48H), 7.35 (t, *J* = 7.4 Hz, 2H), 7.56 (d, *J* = 8.2 Hz, 3.52H), 7.74–7.84 (m, 2H), 11.13 (s, 0.74H), 11.13 (s, 0.26H). ^13^C-APT-NMR (100 MHz, DMSO-*d*_6_) δ 30.9 (1C), 34.8 (1C), 39.2 (1C), 51.9 (1C), 52.8 (1C), 58.7 (1C), 104.5 (1C), 120.9 (1C), 125.2 (min), 125.3 (2C), 126.8 (1C), 127.0 (min), 127.7 (2C), 127.8 (2C), 128.3 (2C), 128.5 (1C), 128.6 (min), 142.7 (1C), 145.7 (1C), 151.1 (1C), 155.6 (1C), 162.4 (1C), 164.7 (1C), 166.4 (1C). IR (neat) (cm^–1^) ν 3261, 2958, 2192, 1731, 1638, 1608, 1438, 1270, 1238, 1118, 849, 698. HRMS (ESI+) calcd for C_27_H_28_N_4_NaO_5_ 511.1948; found 511.1947 [M + Na].

*Dimethyl 6-amino-5-cyano-1-(4-methoxybenzamido)-4-phenyl-1,4-dihydropyridine-2,3-dicarboxylate* (**10fa**): Following the general procedure, compound **10fa** was obtained after 120 h of reaction at room temperature and was purified by column chromatography (*n*-hexane:diethyl ether 20:80 to 0:100), as a yellow solid in 55% yield (25.24 mg). mp 113–115 °C. The ee of the product was determined to be 42% by HPLC using a Daicel Chiralpak IC column (*n*-hexane/*i*-PrOH = 70:30, flow rate 1 mL min^−1^, λ = 253.2 nm): τ_major_ = 32.7 min; τ_minor_ = 14.8 min. [α]_D_^24^ = −25.6 (*c* = 0.08, MeOH, 42% ee). ^1^H-NMR (400 MHz, DMSO-*d*_6_) δ 3.51 (s, 3H), 3.55 (s, 0.75H), 3.62 (s, 2.25H), 3.84 (s, 3H), 4.35 (s, 0.75H), 4.47 (s, 0.25H), 6.33 (s, 1.50H), 6.38 (s, 0.5H), 7.07 (d, *J* = 8.7 Hz, 2H), 7.24 (t, *J* = 7.1 Hz, 1.5H), 7.34 (t, *J* = 7.4 Hz, 2H), 7.55 (d, *J* = 7.5 Hz, 1.5H), 7.77–7.89 (m, 2H), 11.04 (s, 0.75H), 11.16 (s, 0.25H). ^13^C-APT-NMR (100 MHz, DMSO-*d*_6_) δ 39.3 (1C), 51.8 (1C), 52.7 (1C), 55.5 (1C), 58.6 (1C), 104.5 (1C), 113.6 (min), 113.8 (2C), 120.9 (1C), 123.4 (1C), 126.8 (1C), 127.0 (min), 127.7 (2C), 128.3 (2C), 128.6 (min), 129.9 (2C), 142.8 (1C), 145.7 (1C), 151.2 (1C), 162.4 (1C), 162.6 (1C), 164.7 (1C), 166.0 (1C).IR (neat) (cm^–1^) ν 3409, 3334, 3247, 3214, 2953, 2186, 1732, 1713, 1663, 1604, 1587, 1489, 1431, 1249, 1228, 1184, 1115, 1076, 1024, 841, 700. HRMS (ESI+) calcd for C_24_H_22_N_4_NaO_6_ 485.1426; found 485.1425 [M+Na].

*Dimethyl 6-amino-1-benzamido-5-cyano-4-(4-nitrophenyl)-1,4-dihydropyridine-2,3-dicarboxylate* (**10ab**): Following the general procedure, compound **10ab** was obtained after 120 h of reaction at room temperature and was purified by column chromatography (*n*-hexane:diethyl ether 20:80 to 0:100), as a white solid in 95% yield (45.3 mg). mp 158–160 °C. The ee of the product was determined to be 46% by HPLC using a Daicel Chiralpak IA column (*n*-hexane/*i*-PrOH = 70:30, flow rate 1 mL min^−1^, λ = 236.6 nm): τ_major_ = 26.1 min; τ_minor_ = 11.2 min. [α]_D_^25^ = +24.5 (*c* = 0.15, MeOH, 46% ee). ^1^H-NMR (400 MHz, DMSO-*d*_6_) δ 3.51 (s, 3H), 3.64 (s, 3H), 4.50 (s, 1H), 6.62 (s, 2H), 7.54 (t, *J* = 7.4 Hz, 2H), 7.59–7.83 (m, 3H), 7.87 (d, *J* = 7.3 Hz, 2H), 8.24 (d, *J* = 8.7 Hz, 2H), 11.31 (s, 1H). ^13^C-APT-NMR (100 MHz, DMSO-*d*_6_) δ 39.1 (1C), 45.7 (1C), 52.1 (1C), 52.9 (1C), 103.8 (1C), 120.5 (1C), 123.8 (2C), 128.0 (2C), 128.5 (2C), 128.7 (2C), 131.2 (1C), 132.6 (1C), 146.5 (1C), 151.6 (1C), 152.9 (1C), 162.2 (1C), 164.4 (1C), 166.8 (1C). IR (neat) (cm^–1^) ν 3330, 3200, 2953, 2185, 1743, 1708, 1652, 1579, 1516, 1428, 1344, 1225, 1110, 823, 694. HRMS (ESI+) calcd for C_23_H_19_N_5_NaO_7_ 500.1181; found 500.1181 [M + Na].

*Dimethyl 6-amino-1-benzamido-5-cyano-4-(3-nitrophenyl)-1,4-dihydropyridine-2,3-dicarboxylate* (**10ac**) [19]: Following the general procedure, compound **10ac** was obtained after 120 h of reaction at room temperature and was purified by column chromatography (*n*-hexane:diethyl ether 20:80 to 0:100), as a white solid in 89% yield (42.4 mg). The ee of the product was determined to be 51% by HPLC using a Daicel Chiralpak IC column (*n*-hexane/*i*-PrOH = 70:30, flow rate 1 mL min^−1^, λ = 238.9 nm): τ_major_ = 18.6 min; τ_minor_ = 15.4 min. [α]_D_^24^ = −2.8 (*c* = 0.24, MeOH, 51% ee).

*Dimethyl 6-amino-1-benzamido-4-(3-chlorophenyl)-5-cyano-1,4-dihydropyridine-2,3-dicarboxylate* (**10ad**): Following the general procedure, compound **10ad** was obtained after 120 h of reaction at room temperature and was purified by column chromatography (*n*-hexane:diethyl ether 20:80 to 0:100), as a white solid in 70% yield (32.6 mg). mp 134–136 °C. The ee of the product was determined to be 52% by HPLC using a Daicel Chiralpak IC column (*n*-hexane/*i*-PrOH = 80:20, flow rate 1 mL min^−1^, λ = 236.6 nm): τ_major_ = 32.3 min; τ_minor_ = 18.6 min. [α]_D_^24^ = +23.4 (*c* = 0.13, MeOH, 52% ee). ^1^H-NMR (400 MHz, DMSO-*d*_6_) δ 3.53 (s, 3H), 3.65 (s, 3H), 4.41 (s, 1H), 6.49 (s, 2H), 7.31 (d, *J* = 7.4 Hz, 1H), 7.39 (t, *J* = 7.7 Hz, 1H), 7.54 (t, *J* = 7.6 Hz, 3H), 7.60–7.68 (m, 2H), 7.86 (d, *J* = 7.3 Hz, 2H), 11.27 (s, 1H). ^13^C-APT-NMR (100 MHz, DMSO-*d*_6_) δ 39.0 (1C), 52.0 (1C), 52.9 (1C), 58.1 (1C), 104.3 (1C), 120.6 (1C), 126.4 (1C), 127.0 (1C), 127.6 (1C), 127.9 (2C), 128.5 (2C), 130.2 (1C), 131.3 (1C), 132.5 (1C), 133.2 (1C), 142.9 (1C), 148.1 (1C), 151.3 (1C), 162.3 (1C), 164.5 (1C), 166.8 (1C). IR (neat) (cm^–1^) ν 3330, 2953, 2922, 2850, 2184, 1736, 1707, 1654, 1578, 1430, 1227, 1115, 1079, 886, 781, 692. HRMS (ESI+) calcd for C_23_H_19_ClN_4_NaO_5_ 489.0942; found 489.0941 [M + Na].

*Dimethyl 6-amino-1-benzamido-4-(4-chlorophenyl)-5-cyano-1,4-dihydropyridine-2,3-dicarboxylate* (**10ae**) [19]: Following the general procedure, compound **10ae** was obtained after 120 h of reaction at room temperature and was purified by column chromatography (*n*-hexane:diethyl ether 20:80 to 0:100), as a white solid in 70% yield (32.7 mg). The ee of the product was determined to be 50% by HPLC using a Daicel Chiralpak IC column (*n*-hexane/*i*-PrOH = 80:20, flow rate 1 mL min^−1^, λ = 237.7 nm): τ_major_ = 22.5 min; τ_minor_ = 16.4 min. [α]_D_^24^ = −2.2 (*c* = 0.15, MeOH, 50% ee).

*Dimethyl 6-amino-1-benzamido-4-(4-bromophenyl)-5-cyano-1,4-dihydropyridine-2,3-dicarboxylate* (**10af**) [19]: Following the general procedure, compound **10af** was obtained after 120 h of reaction at room temperature and was purified by column chromatography (*n*-hexane:diethyl ether 20:80 to 0:100), as a white solid in 61% yield (31.2 mg). The ee of the product was determined to be 52% by HPLC using a Daicel Chiralpak IC column (*n*-hexane/*i*-PrOH = 80:20, flow rate 1 mL min^−1^, λ = 236.6 nm): τ_major_ = 23.0 min; τ_minor_ = 18.0 min. [α]_D_^24^ = −19.5 (*c* = 0.05, MeOH, 52% ee).

*Dimethyl 6-amino-1-benzamido-5-cyano-4-(4-cyanophenyl)-1,4-dihydropyridine-2,3-dicarboxylate* (**10ag**): Following the general procedure, compound **10ag** was obtained after 120 h of reaction at room temperature and was purified by column chromatography (*n*-hexane:diethyl ether 20:80 to 0:100), as a white solid in 99% yield (45.09 mg). mp 154–156 °C. The ee of the product was determined to be 50% by HPLC using a Daicel Chiralpak IC column (*n*-hexane/*i*-PrOH = 70:30, flow rate 1 mL min^−1^, λ = 234.2 nm): τ_major_ = 22.2 min; τ_minor_ = 18.5 min. [α]_D_^24^ = +4.0 (*c* = 0.07, MeOH, 50% ee). ^1^H-NMR (400 MHz, DMSO-*d*_6_) δ 3.51 (s, 3H), 3.65 (s, 3H), 4.49 (s, 1H), 6.55 (s, 2H), 7.35–7.69 (m,4H), 7.70–7.91 (m, 5H), 11.30 (s, 1H). ^13^C-APT-NMR (100 MHz, DMSO-*d*_6_) δ 39.4 (1C), 52.0 (1C), 52.9 (1C), 57.5 (1C), 103.9 (1C), 109.8 (1C), 118.9 (1C), 120.5 (1C), 127.9 (3C), 128.5 (2C), 128.6 (2C), 131.2 (1C), 132.5 (2C), 143.3 (1C), 151.0 (1C), 151.4 (1C), 162.2 (1C), 164.4 (1C), 166.8 (1C). IR (neat) (cm^–1^) ν 3414, 3313, 3210, 2947, 2231, 2192, 1750, 1704, 1682, 1665, 1590, 1433, 1360, 1272, 1253, 1222, 1115, 929, 846, 686. HRMS (ESI+) calcd for C_24_H_19_N_5_NaO_5_ 480.1258; found 480.1256 [M + Na].

*Dimethyl 6-amino-1-benzamido-5-cyano-4-(naphthalen-1-yl)-1,4-dihydropyridine-2,3-dicarboxylate* (**10ah**): Following the general procedure, compound **10ah** was obtained after 120 h of reaction at room temperature and was purified by column chromatography (*n*-hexane:diethyl ether 20:80 to 0:100), as a yellow solid in 51% yield (24.61 mg). mp 122–124 °C. The ee of the product was determined to be 50% by HPLC using a Daicel Chiralpak IC column (*n*-hexane/*i*-PrOH = 70:30, flow rate 1 mL min^−1^, λ = 221.2 nm): τ_major_ = 27.9 min; τ_minor_ = 16.5 min. [α]_D_^24^ = −28.9 (*c* = 0.10, MeOH, 50% ee). ^1^H-NMR (400 MHz, DMSO-*d*_6_) δ 3.33 (s, 3H), 3.60 (s, 0.75H), 3.67 (s, 2.25H), 5.42 (s, 0.75H), 5.47 (s, 0.25H), 6.33 (s, 1.5H), 6.38 (s, 0.5H), 7.41–7.70 (m, 6.25H), 7.78–7.97 (m, 4H), 8.09 (d, *J* = 6.7 Hz, 0.75H), 8.46 (d, *J* = 8.7 Hz, 1H), 11.27 (s, 0.75H), 11.39 (s, 0.25H). ^13^C-APT-NMR (100 MHz, DMSO-*d*_6_) δ 40.0 (1C), 51.7 (1C), 52.8 (1C), 59.2 (1C), 64.9 (1C), 105.4 (1C), 120.8 (1C), 123.5 (1C), 125.5 (1C), 125.7 (1C), 126.0 (1C), 127.0 (1C), 127.1 (1C), 127.9 (2C), 128.3 (1C), 128.5 (2C), 130.3 (1C), 131.4 (1C), 132.5 (1C), 133.1 (1C), 143.0 (1C), 151.1 (1C), 162.5 (1C), 164.8 (1C), 166.7 (1C). IR (neat) (cm^–1^) ν 3202, 2951, 2194, 1715, 1704, 1661, 1592, 1510, 1426, 1334, 1232, 1070, 782. HRMS (ESI+) calcd for C_27_H_22_N_4_NaO_5_ 505.1482; found 505.1454 [M + Na].

*Dimethyl 6-amino-1-benzamido-5-cyano-4-(p-tolyl)-1,4-dihydropyridine-2,3-dicarboxylate* (**10ai**) [19]: Following the general procedure, compound **10ai** was obtained after 120 h of reaction at room temperature and was purified by column chromatography (*n*-hexane:diethyl ether 20:80 to 0:100), as a white solid in 44% yield (19.6 mg). The ee of the product was determined to be 50% by HPLC using a Daicel Chiralpak IC column (*n*-hexane/*i*-PrOH = 70:30, flow rate 1 mL min^−1^, λ = 237.7 nm): τ_major_ = 15.9 min; τ_minor_ = 9.6 min. [α]_D_^24^ = −19.4 (*c* = 0.12, MeOH, 50% ee).

*Dimethyl 6-amino-1-benzamido-5-cyano-4-(4-methoxyphenyl)-1,4-dihydropyridine-2,3-dicarboxylate* (**10aj**): Following the general procedure, compound **10aj** was obtained after 120 h of reaction at room temperature and was purified by column chromatography (*n*-hexane:diethyl ether 20:80 to 0:100), as a white solid in 40% yield (18.57 mg). mp 137–139 °C. The ee of the product was determined to be 40% by HPLC using a Daicel Chiralpak IC column (*n*-hexane/*i*-PrOH = 70:30, flow rate 1 mL min^−1^, λ = 237.7 nm): τ_major_ = 48.4 min; τ_minor_ = 19.2 min. [α]_D_^24^ = −24.0 (*c* = 0.05, MeOH, 40% ee). ^1^H-NMR (400 MHz, DMSO-*d*_6_) 3.52 (s, 3H), 3.55 (s, 0.75H), 3.63 (s, 2.25H), 3.75 (s, 3H), 4.30 (s, 0.75H), 4.42 (s, 0.25H), 6.32 (s, 1.5H), 6.38 (s, 0.50H), 6.89 (d, *J* = 8.5 Hz, 2.25H), 7.10–7.22 (m, 0.75H), 7.43–7.68 (m, 4H), 7.79–7.88 (m, 2H), 11.19 (s, 0.75H), 11.30 (s, 0.25H). ^13^C-APT-NMR (100 MHz, DMSO-*d*_6_) δ 38.4 (1C), 51.8 (1C), 52.7 (1C), 55.0 (1C), 58.9 (1C), 104.9 (1C), 113.6 (2C), 113.9 (min), 120.9 (1C), 127.9 (2C), 128.5 (2C), 128.8 (2C), 131.3 (1C), 132.5 (1C), 137.9 (1C), 142.2 (1C), 151.0 (1C), 158.2 (1C), 162.5 (1C), 164.8 (1C), 166.7 (1C). IR (neat) (cm^–1^) ν 3313, 3201, 2952, 2838, 2186, 1742, 1707, 1651, 1606, 1509, 1428, 1227, 1175, 1110, 1028, 833, 691. HRMS (ESI+) calcd for C_24_H_22_N_4_NaO_6_ 485.1438; found 485.1435 [M + Na].

*Dimethyl 6-amino-1-benzamido-5-cyano-4-(furan-2-yl)-1,4-dihydropyridine-2,3-dicarboxylate* (**10ak**): Following the general procedure, compound **10ak** was obtained after 120 h of reaction at room temperature and was purified by column chromatography (*n*-hexane:diethyl ether 20:80 to 0:100), as a white solid in 70% yield (29.77 mg). mp 151–153 °C. The ee of the product was determined to be 54% by HPLC using a Daicel Chiralpak IA column (*n*-hexane/*i*-PrOH = 80:20, flow rate 1 mL min^−1^, λ = 236.6 nm): τ_major_ = 16.4 min; τ_minor_ = 12.4 min. [α]_D_^24^ = −15.3 (*c* = 0.10, MeOH, 54% ee). ^1^H-NMR (400 MHz, DMSO-*d*_6_) δ 3.55 (s, 1.35H), 3.58 (s, 1.35H), 3.59 (s, 1.65H), 3.65 (s, 1.65H), 4.51 (s, 0.55H), 4.64 (s, 0.45H), 6.11 (d, *J* = 3.1 Hz, 0.45H), 6.33 (d, *J* = 3.1 Hz, 0.55H), 6.37–6.45 (m, 1H), 6.50 (s, 1.1H), 6.57 (s, 0.9H), 7.46–7.67 (m, 4H), 7.77–7.86 (m, 2H), 11.20 (s, 0.55H), 11.38 (s, 0.45H). ^13^C-APT-NMR (100 MHz, DMSO-*d*_6_) δ 32.4 (1C), 33.0 (min), 52.0 (min), 52.1 (1C), 52.7 (min), 52.9 (1C), 53.8 (min), 55.5 (1C), 101.8 (min), 102.3 (1C), 105.3 (min), 106.0 (1C), 110.6 (min), 110.8 (1C), 120.8 (1C), 120.9 (min), 127.8 (1C), 128.0 (min), 128.3 (min), 128.5 (1C), 131.3 (1C), 131.4 (min), 132.3 (min), 132.5 (1C), 141.6 (1C), 142.3 (min), 143.1 (1C), 143.7 (min), 152.0 (1C), 152.6 (min), 156.4 (min), 157.6 (1C), 162.1 (min), 162.2 (1C), 164.5 (1C), 164.7 (min), 166.1 (min), 166.2 (1C). IR (neat) (cm^–1^) ν 3527, 3406, 3328, 2175, 1735, 1707, 1652, 1578, 1431, 1336, 1225, 1013, 939, 754, 705. HRMS (ESI+) calcd for C_21_H_18_N_4_NaO_6_ 445.1125; found 445.1125 [M + Na].

*Dimethyl 6-amino-1-benzamido-5-cyano-4-(thiophen-2-yl)-1,4-dihydropyridine-2,3-dicarboxylate* (**10al**): Following the general procedure, compound **10al** was obtained after 120 h of reaction at room temperature and was purified by column chromatography (*n*-hexane:diethyl ether 20:80 to 0:100), as a white solid in 76% yield (33.18 mg). mp 124–126 °C. The ee of the product was determined to be 43% by HPLC using a Daicel Chiralpak IC column (*n*-hexane/*i*-PrOH = 70:30, flow rate 1 mL min^−1^, λ = 236.6 nm): τ_major_ = 27.1 min; τ_minor_ = 16.1 min. [α]_D_^24^ = −12.6 (*c* = 0.10, MeOH, 43% ee). ^1^H-NMR (400 MHz, DMSO-*d*_6_) δ 3.55 (s, 1.11H), 3.60 (s, 3H), 3.64 (s, 1.89H), 4.66 (s, 0.63H), 4.82 (s, 0.37H), 6.50 (s, 1.26H), 6.57 (s, 0.74H), 6.88 (s, 0.37H), 7.00 (s, 1H), 7.19 (s, 0.63H), 7.39 (d, *J* = 4.7 Hz, 1H), 7.52 (t, *J* = 6.4 Hz, 2H), 7.62 (t, *J* = 6.7 Hz, 1H), 7.83 (d, *J* = 7.4 Hz, 2H), 11.21 (s, 0.63H), 11.38 (d, 0.37H). ^13^C-APT-NMR (100 MHz, DMSO-*d*_6_) δ 33.7 (1C), 52.0 (1C), 52.8 (1C), 57.9 (1C), 104.3 (1C), 120.8 (1C), 124.5 (1C), 124.9 (1C), 127.1 (1C), 127.9 (2C), 128.3 (min), 128.5 (2C), 131.4 (1C), 132.4 (1C), 142.3 (1C), 148.7 (1C), 151.6 (1C), 162.2 (1C), 164.5 (1C), 166.2 (1C). IR (neat) (cm^–1^) ν 3415, 3332, 3248, 3213, 2954, 2191, 1719, 1685, 1660, 1587, 1434, 1337, 1230, 941, 707, 687. HRMS (ESI+) calcd for C_21_H_18_N_4_NaO_5_S 461.0898; found 461.0897 [M + Na].

*Dimethyl 6′-amino-1′-benzamido-5′-cyano-1′,4′-dihydro-[3,4′-bipyridine]-2′,3′-dicarboxylate* (**10am**): Following the general procedure, compound **10am** was obtained after 120 h of reaction at room temperature and was purified by column chromatography (*n*-hexane:diethyl ether 20:80 to 0:100 to ethyl acetate 100), as a white solid in 92% yield (39.66 mg). mp 157–159 °C. The ee of the product was determined to be 40% by HPLC using a Daicel Chiralpak IC column (*n*-hexane/*i*-PrOH = 70:30, flow rate 1 mL min^−1^, λ = 336.0 nm): τ_major_ = 32.9 min; τ_minor_ = 50.6 min. [α]_D_^24^ = −12.6 (*c* = 0.17, MeOH, 40% ee). ^1^H-NMR (400 MHz, DMSO-*d*_6_) δ 3.52 (s, 3H), 3.64 (s, 3H), 4.44 (s, 1H), 6.53 (s, 2H), 7.42 (dd, *J* = 8 Hz, 5 Hz, 1H), 7.52 (t, *J* = 7.5 Hz, 2H), 7.63 (t, *J* = 7.3 Hz, 1H), 7.87 (d, *J* = 7.3 Hz, 2H), 8.05 (s, 1H), 8.47 (dd, *J* = 4.7 Hz, 1.6 Hz, 1H), 8.67 (s, 1H), 11.31 (s, 1H). ^13^C-APT-NMR (100 MHz, DMSO-*d*_6_) δ 36.9 (1C), 45.8 (1C), 52.0 (1C), 52.9 (1C), 104.1 (1C), 120.6 (1C), 123.8 (1C), 127.9 (2C), 128.5 (2C), 131.2 (1C), 132.6 (1C), 135.3 (1C), 141.0 (1C), 143.1 (1C), 148.2 (1C), 148.7 (1C), 151.5 (1C), 162.2 (1C), 164.4 (1C), 166.8 (1C). IR (neat) (cm^–1^) ν 3317, 3226, 2952, 2185, 1749, 1707, 1685, 1654, 1578, 1425, 1330, 1275, 1221, 1115, 1029, 703. HRMS (ESI+) calcd for C_22_H_20_N_5_O_5_ 434.1468; found 434.1467 [M + H].

## 4. Conclusions

In summary, we have developed the first organocatalytic enantioselective approach for the obtainment of chiral 1-benzamido-1,4-dihydropyridines. Final 1,4-dihydropyridines were reached with high yields and showed promising results of enantioselectivity for the first time, using mild conditions and following a simple approach. Further mechanistic studies are required in order to understand and to prove the role of the β-isocupreidine catalyst in this process. Moreover, additional studies with the aim of improving the enantioselectivity of the method are actively ongoing in our laboratory. 

## Figures and Tables

**Table 1:**
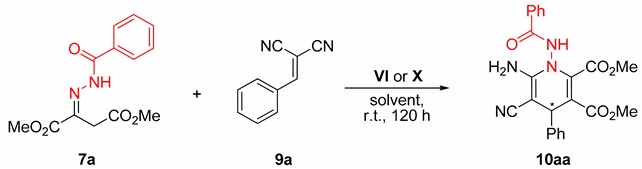
Screening of the reaction conditions for the synthesis of chiral 1-benzamido-1,4-dihydropyridine **10aa** ^a^.

Entry	Solvent (mL)	7a (mmol)	9a (mmol)	Cat. (mol%)	Yield (%) ^b^	ee (%) ^c^
1	MeCN (0.5)	0.1	0.1	**VI** (20%)	>95	7
2	MeCN (0.5)	0.1	0.1	**X** (20%)	56	10
3	AcOEt (0.5)	0.1	0.1	**VI** (20%)	72	20
4	AcOEt (0.5)	0.1	0.1	**X** (20%)	54	40
5	CH_2_Cl_2_ (0.5)	0.1	0.1	**VI** (20%)	67	15
6	CH_2_Cl_2_ (0.5)	0.1	0.1	**X** (20%)	35	Rac. ^d^
7	CHCl_3_ (0.5)	0.1	0.1	**VI** (20%)	44	24
8	CHCl_3_ (0.5)	0.1	0.1	**X** (20%)	49	33
9	Et_2_O (0.5)	0.1	0.1	**VI** (20%)	26	25
10	Et_2_O (0.5)	0.1	0.1	**X** (20%)	39	40
11	THF (0.5)	0.1	0.1	**VI** (20%)	54	17
12	THF (0.5)	0.1	0.1	**X** (20%)	54	50
13	MeOH (0.5)	0.1	0.1	**VI** (20%)	>95	Rac. ^d^
14	MeOH (0.5)	0.1	0.1	**X** (20%)	77	Rac. ^d^
15	THF (0.5)	0.1	0.15	**X** (20%)	61	50
16	THF (0.5)	0.15	0.1	**X** (20%)	51	49
17	THF (0.25)	0.1	0.15	**X** (20%)	58	46
18	THF (1)	0.1	0.15	**X** (20%)	48	52
19	THF (0.5)	0.1	0.15	**X** (10%)	57	48
20	THF (0.5)	0.1	0.15	**X** (30%)	61	46

^a^ Otherwise indicated: To a mixture of catalyst **VI** or **X** (20 mol%) and hydrazone **7a** (0.1 mmol), in the corresponding solvent (0.5 mL), alkylidenemalononitrile **9a** (0.1 mmol) was added. ^b^ Isolated yield after column chromatography (SiO_2_, Hex:Et_2_O 20:80 to Hex:Et_2_O 0:100). ^c^ Determined by chiral HPLC analysis (Daicel Chiralpak IC, Hex:*i*PrOH 70:30, 1 mL/min). ^d^ Racemic mixture.

## Supplementary Materials

The following are available online. Figures S1–S5: ^1^H and ^13^C-APT NMR spectra of hydrazones **7**. Figures S6–S19: ^1^H and ^13^C-APT NMR spectra of 1,4-dihydropyridines **10** Figures S20–S57: HPLC chromatograms of 1,4-dihydropyridines **10**.
