# Supplementary material for: First Organocatalytic Asymmetric Synthesis of 1-Benzamido-1,4-Dihydropyridine Derivatives
Source: Molecules. 2018 Oct 19;23(10):2692. doi: 10.3390/molecules23102692 (PMC6222298; doi:10.3390/molecules23102692)
Supplement: Supplementary file 1 [file molecules-23-02692-s001.pdf]

First organocatalytic asymmetric synthesis of 1-benzamido-1,4-dihydropyridine derivatives

Fernando Auria-Luna [1], Eugenia Marqués-López [1] and Raquel P. Herrera [1,*]

[1] Laboratorio de Organocatálisis Asimétrica. Departamento de Química Orgánica. Instituto de Síntesis Química y Catálisis Homogénea (ISQCH) CSIC-Universidad de Zaragoza. C/ Pedro Cerbuna 12, E- 50009 Zaragoza (Spain); e-mail: ferauria@unizar.es (F.A.-L.); mmaamarq@unizar.es (E.M.-L.)

* Correspondence: (R.P.H.) raquelph@unizar.es; Tel.: +34-97676-1190

Electronic Supplementary Information (ESI)

Figure S1. ^{1}H and ^{13}C-APT NMR spectra of (E)-dimethyl 2-(2-(4-nitrobenzoyl)hydrazono)succinate (7b)

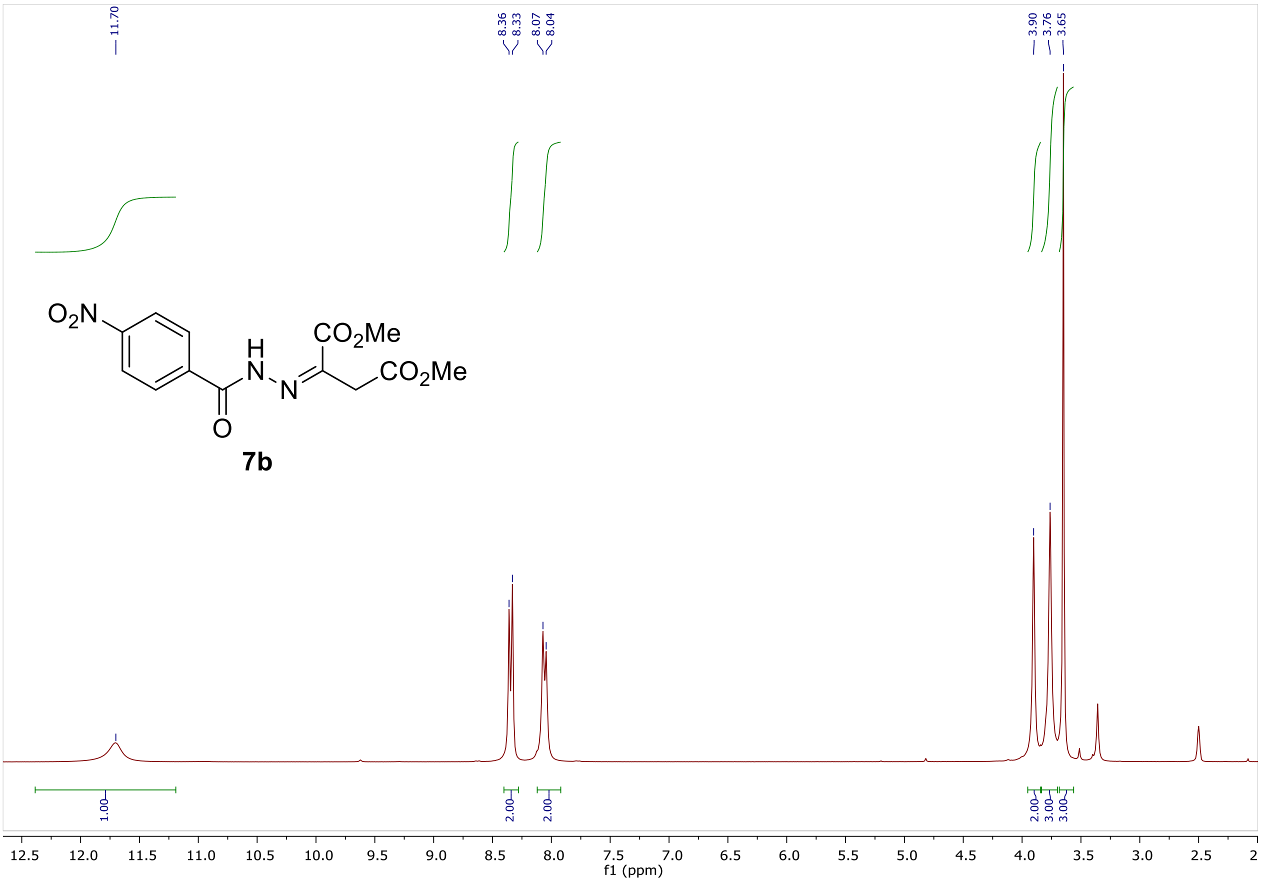

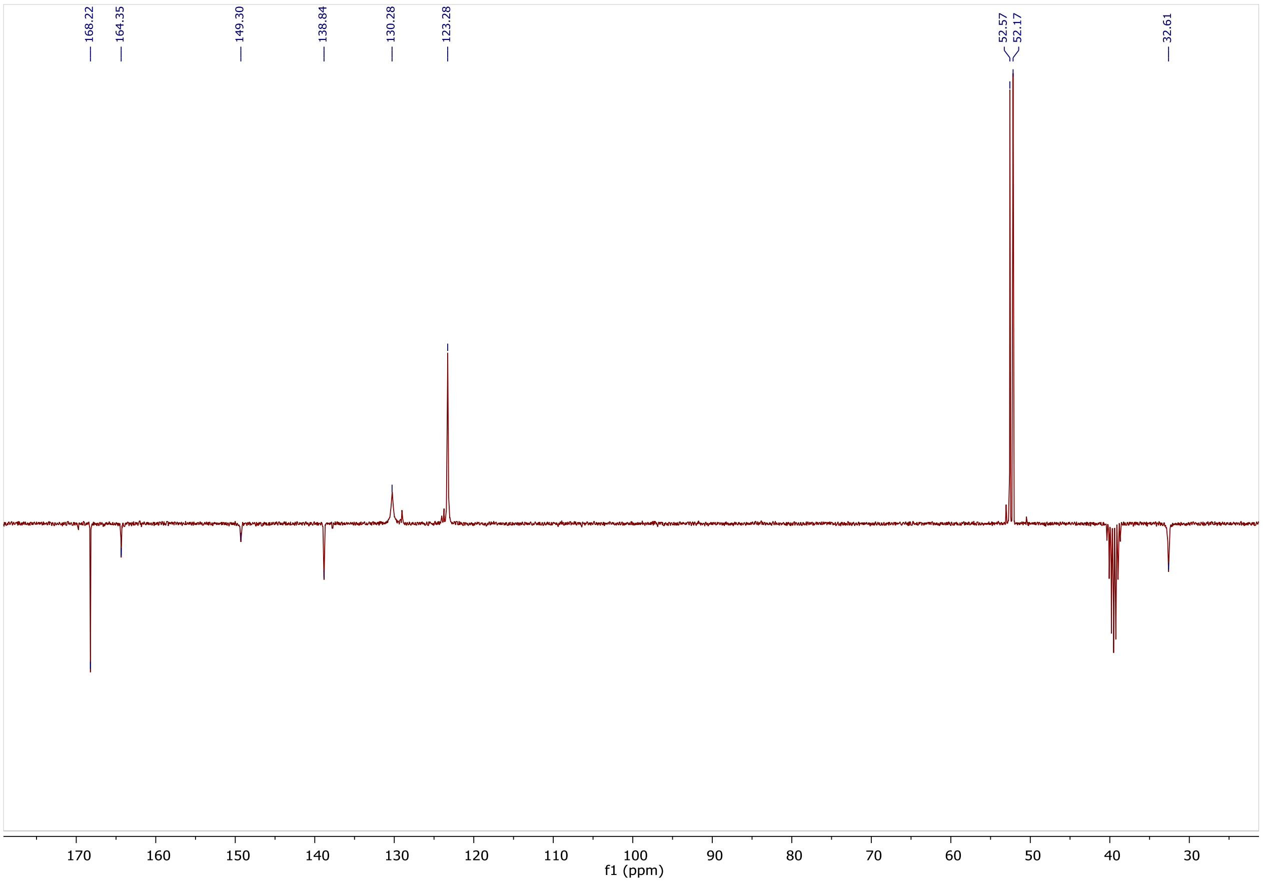

Figure S2. ^{1}H **and** ^{13}C**-APT NMR spectra of (*E*)-dimethyl 2-(2-(4-chlorobenzoyl)hydrazono)succinate (7c)**

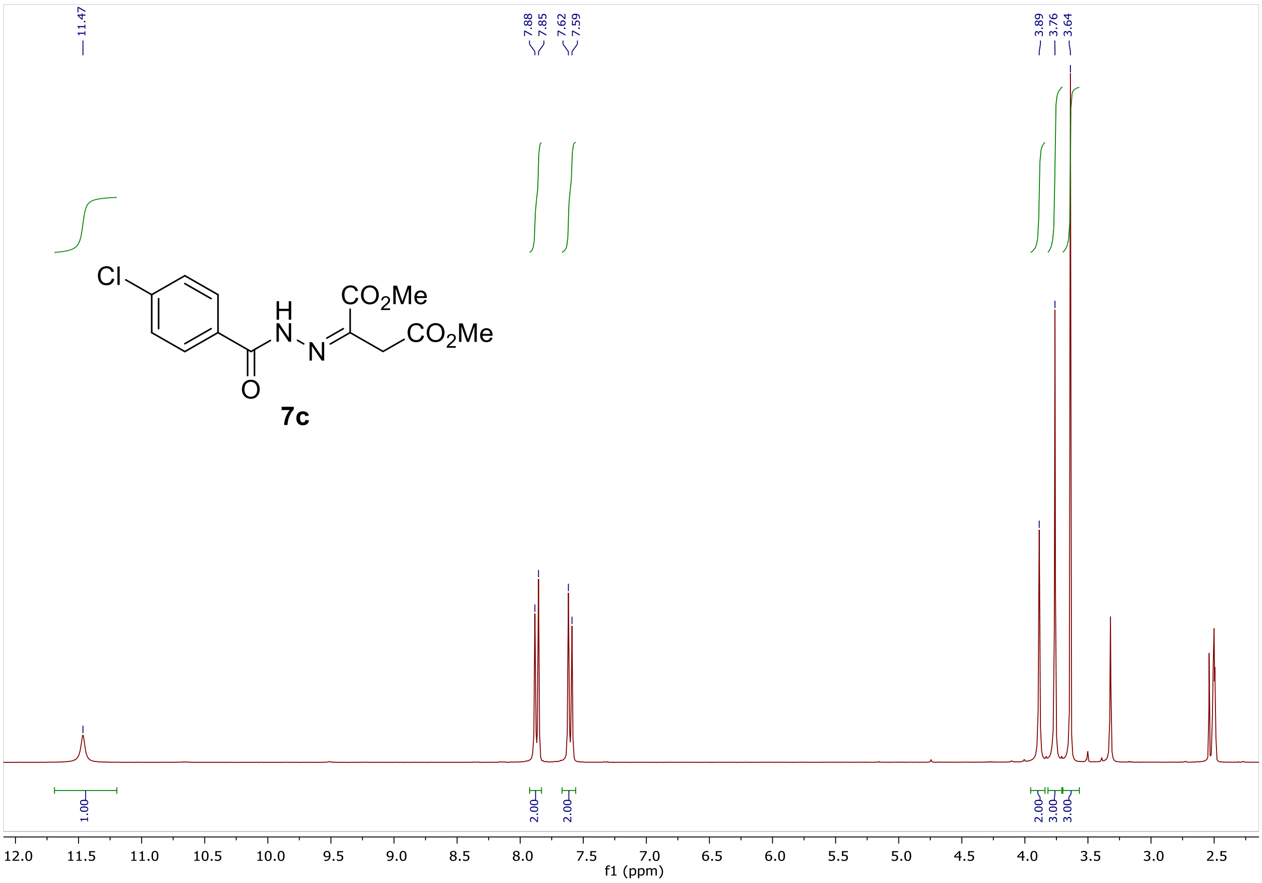

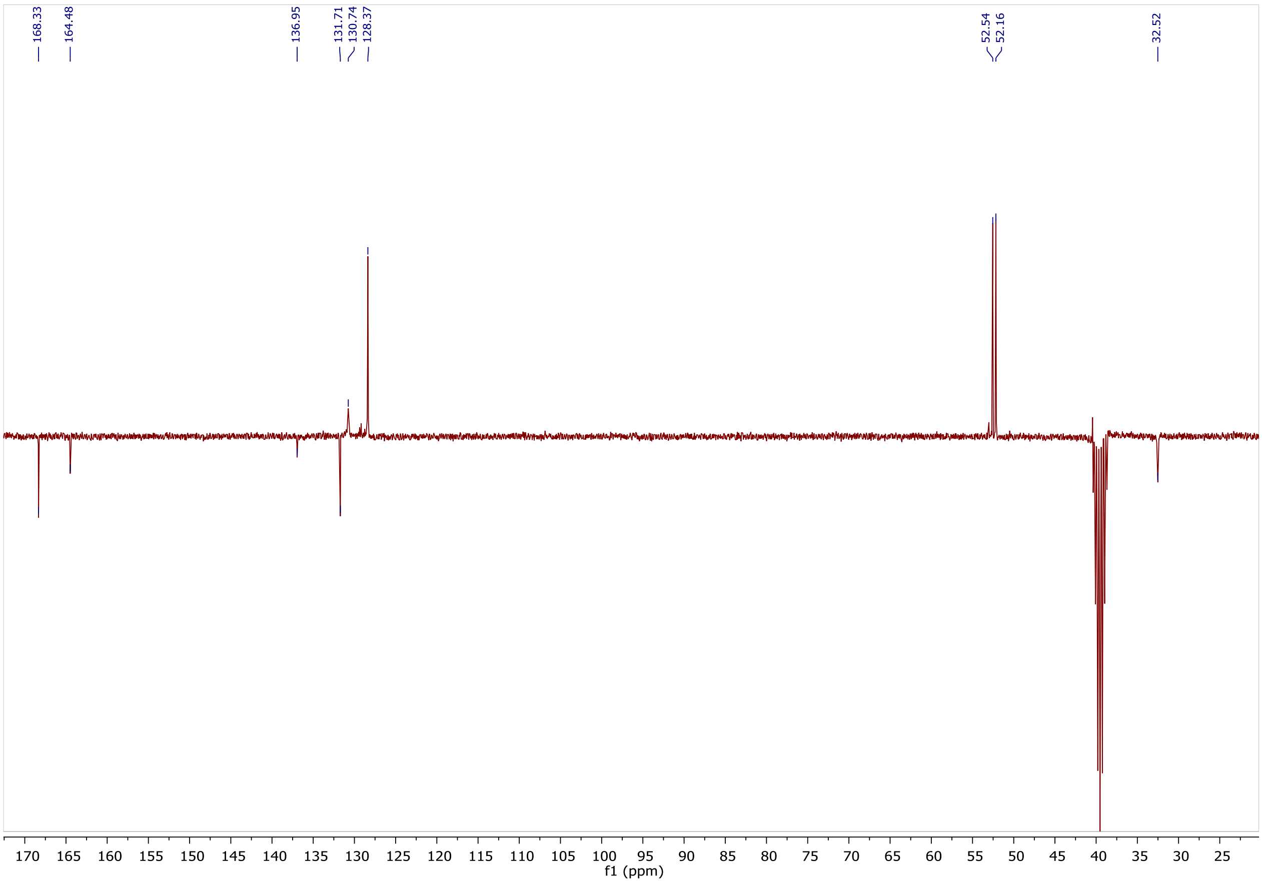

Figure S3. ^{1}H **and** ^{13}C**-APT NMR spectra of (E)-dimethyl 2-(2-(4-bromobenzoyl)hydrazono)succinate (7d)**

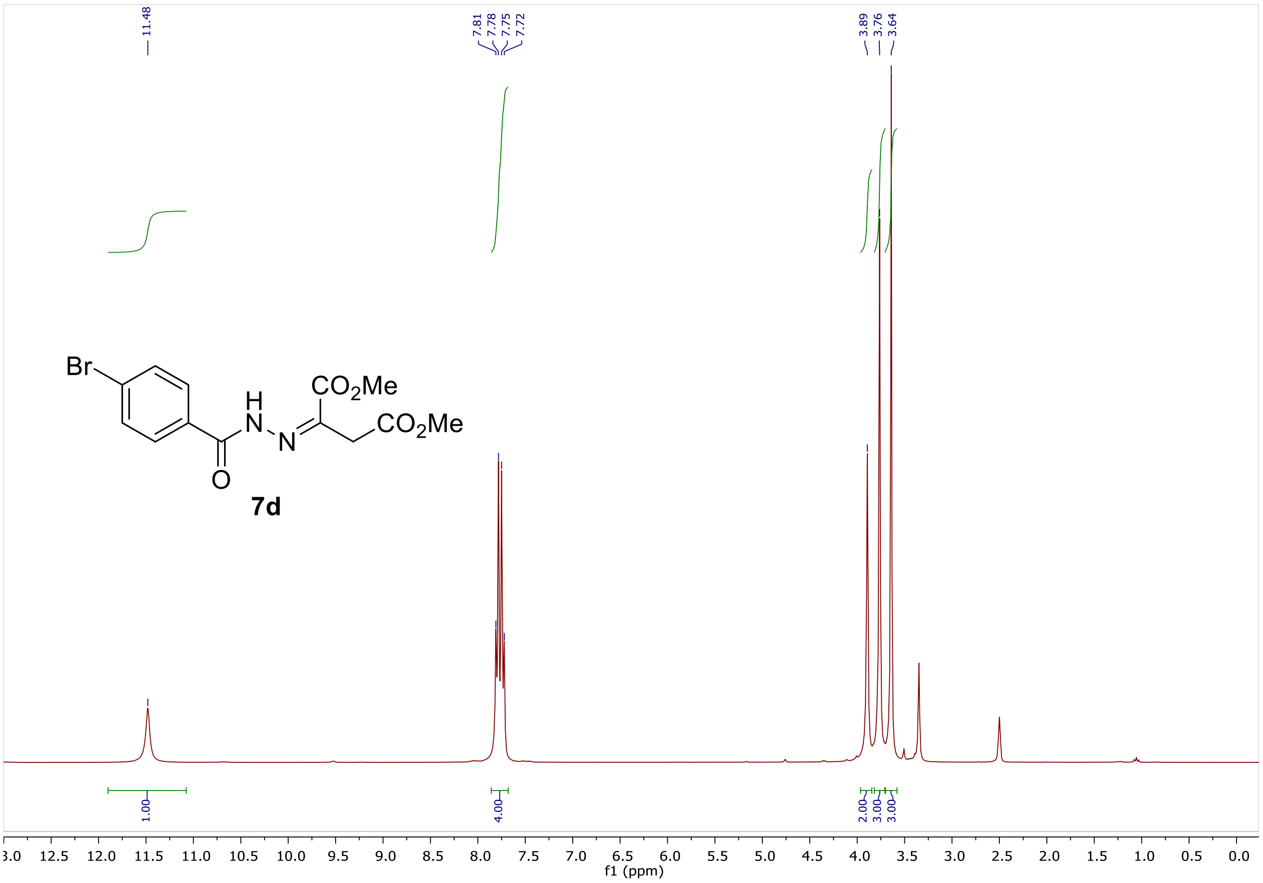

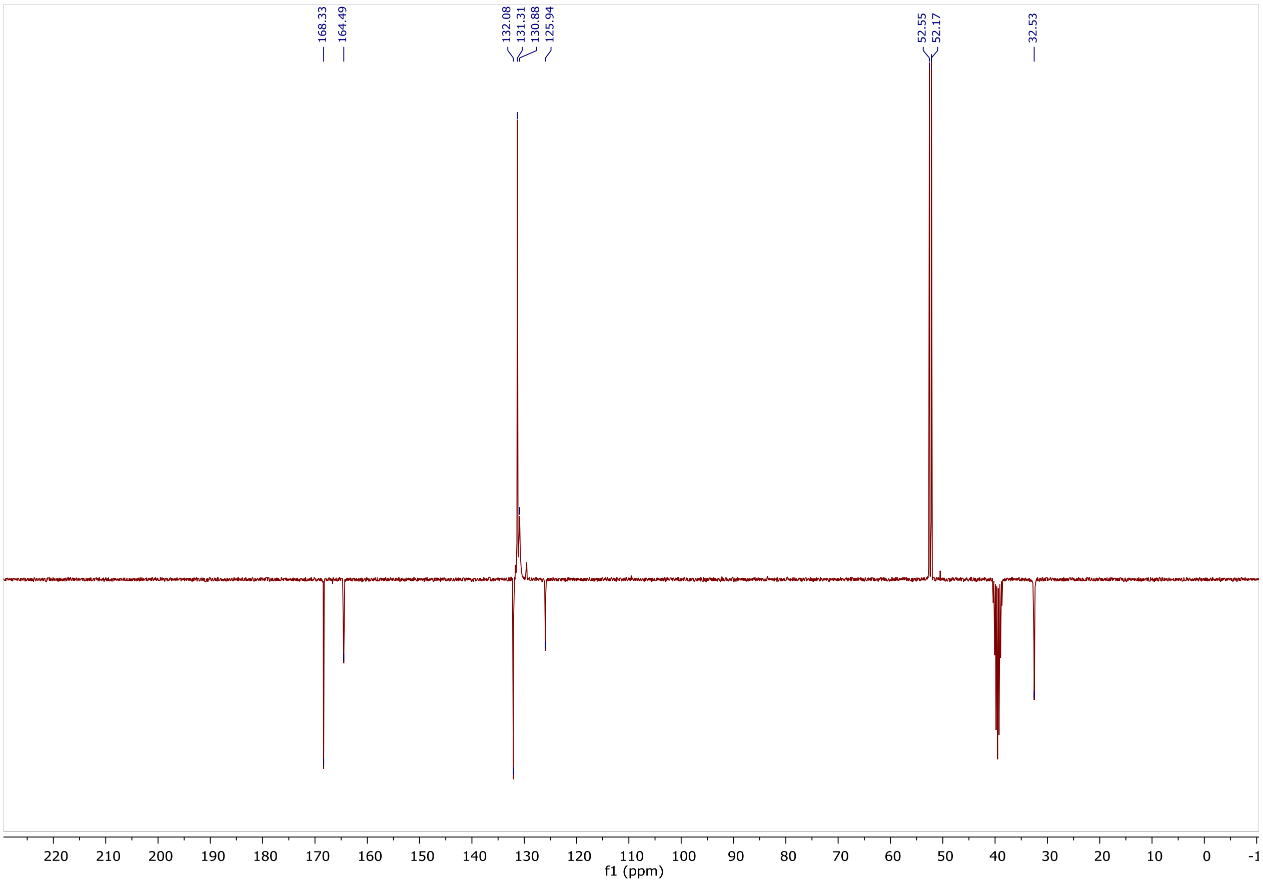

Figure S4. ^{1}H **and** ^{13}C**-APT NMR spectra of (E)-dimethyl 2-(2-(4-tert-butylbenzoyl)hydrazono)succinate (7e)**

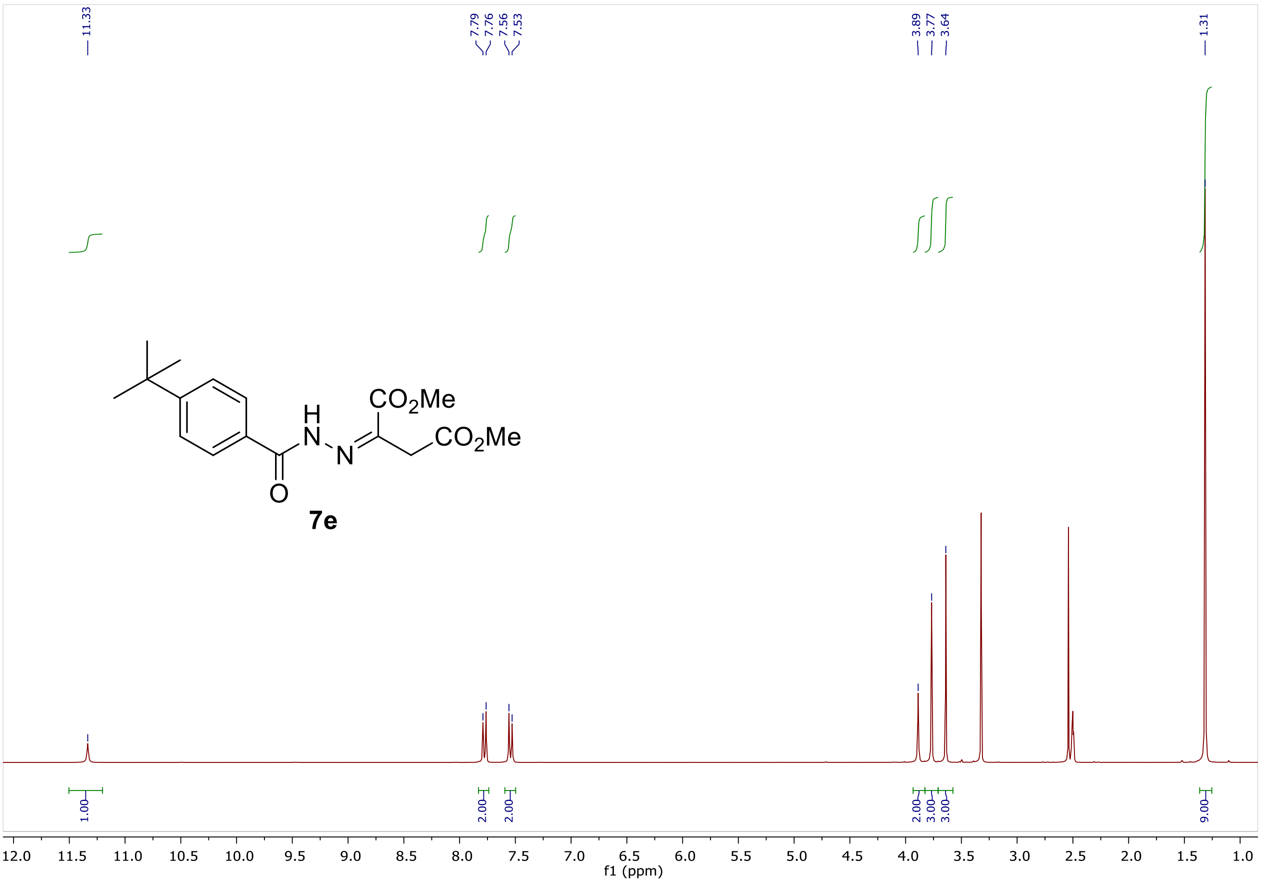

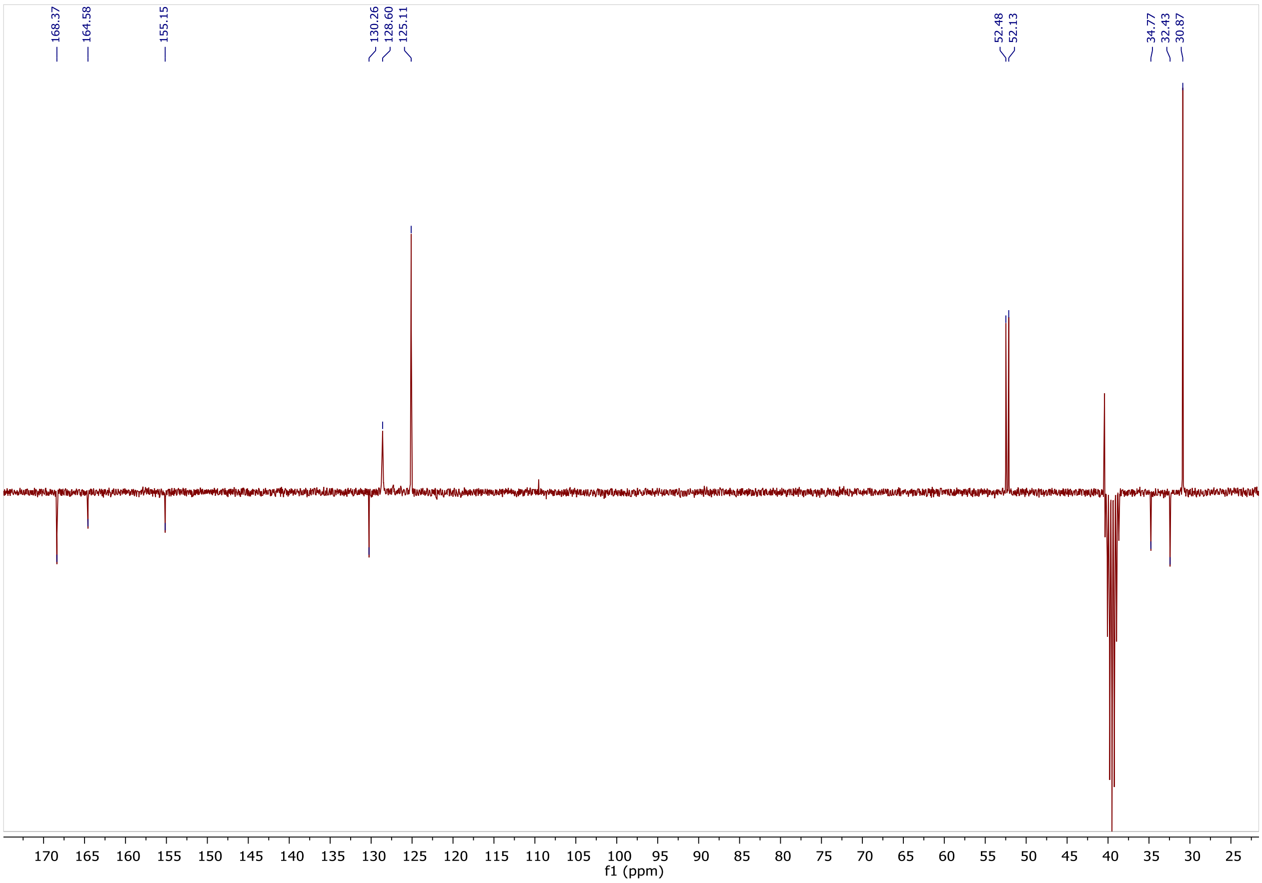

Figure S5. ^{1}H and ^{13}C-APT NMR spectra of (E)-dimethyl 2-(2-(4-methoxybenzoyl)hydrazono)succinate (7f)

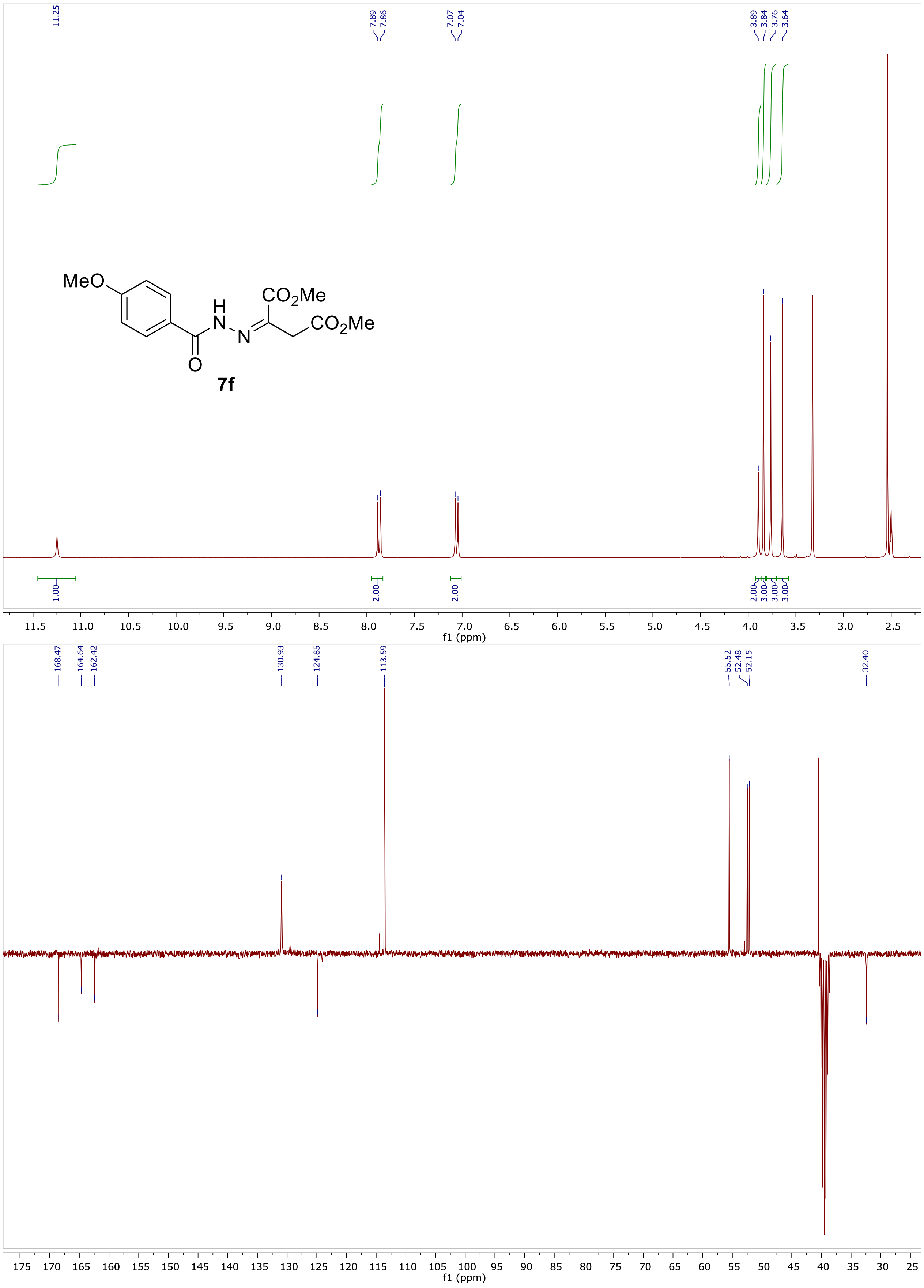

Figure S6. ^{1}H and ^{13}C-APT NMR spectra of dimethyl 6-amino-1-benzamido-5-cyano-4-phenyl-1,4-dihydropyridine-2,3-dicarboxylate (10aa)

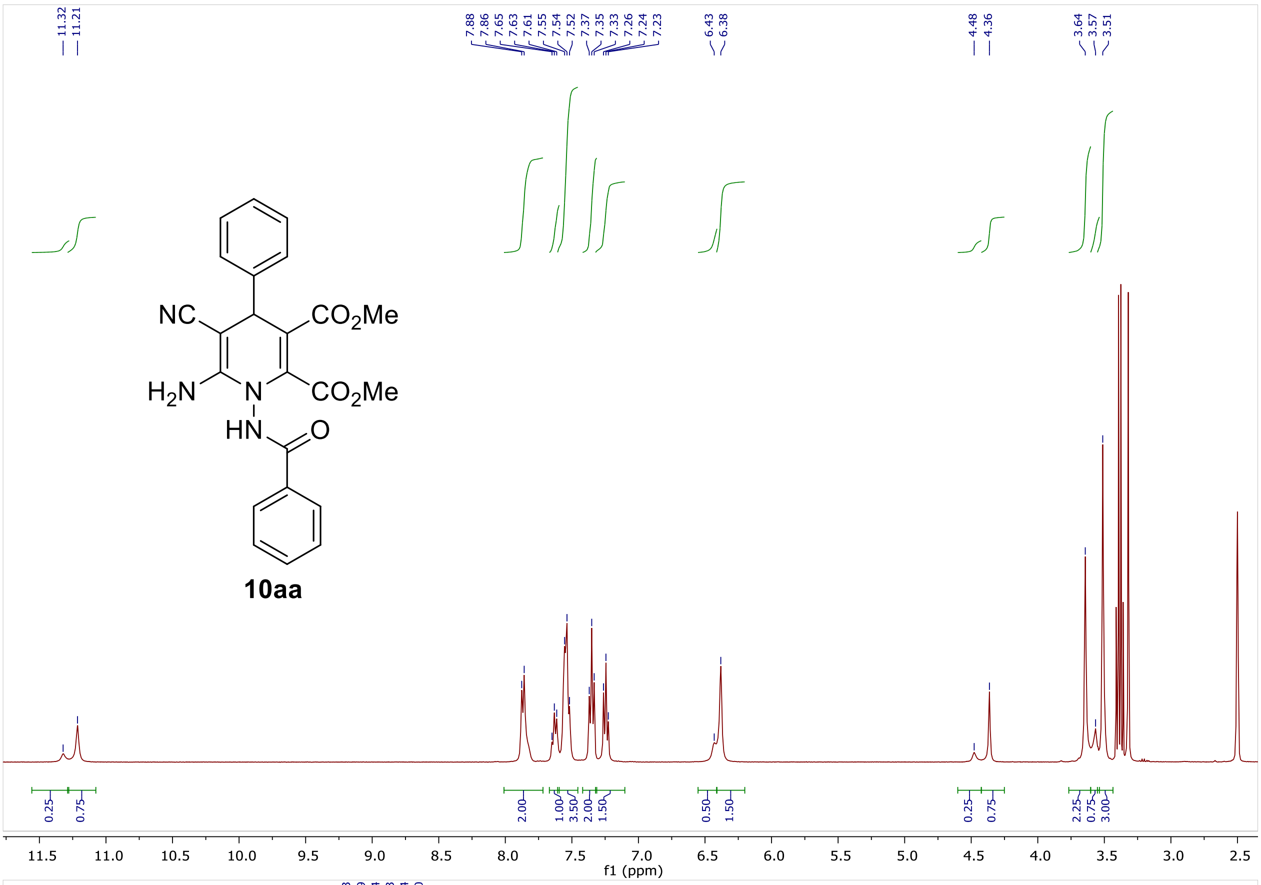

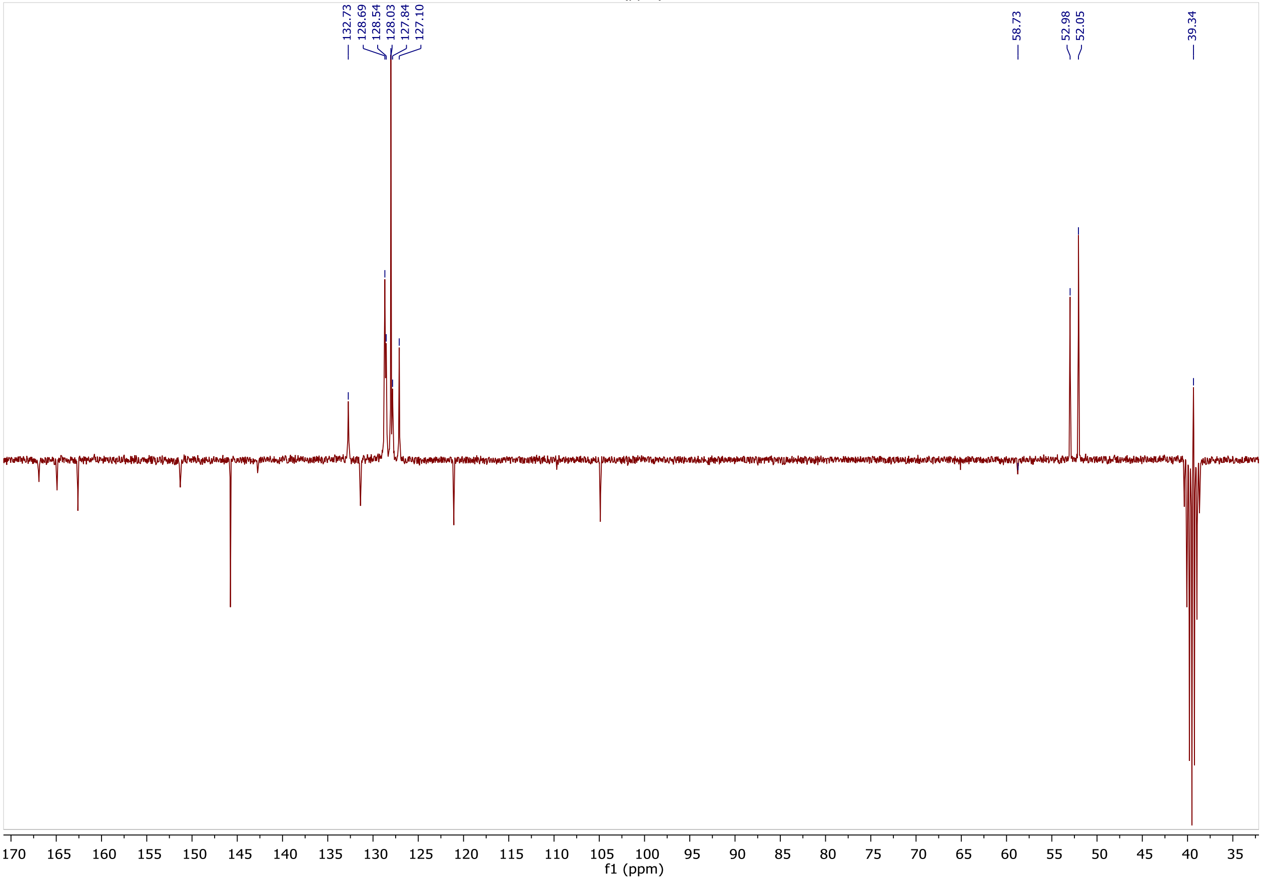

Figure S7. ^{1}H and ^{13}C-APT NMR spectra of dimethyl 6-amino-5-cyano-1-(4-nitrobenzamido)-4-phenyl-1,4-dihydropyridine-2,3-dicarboxylate (10ba)

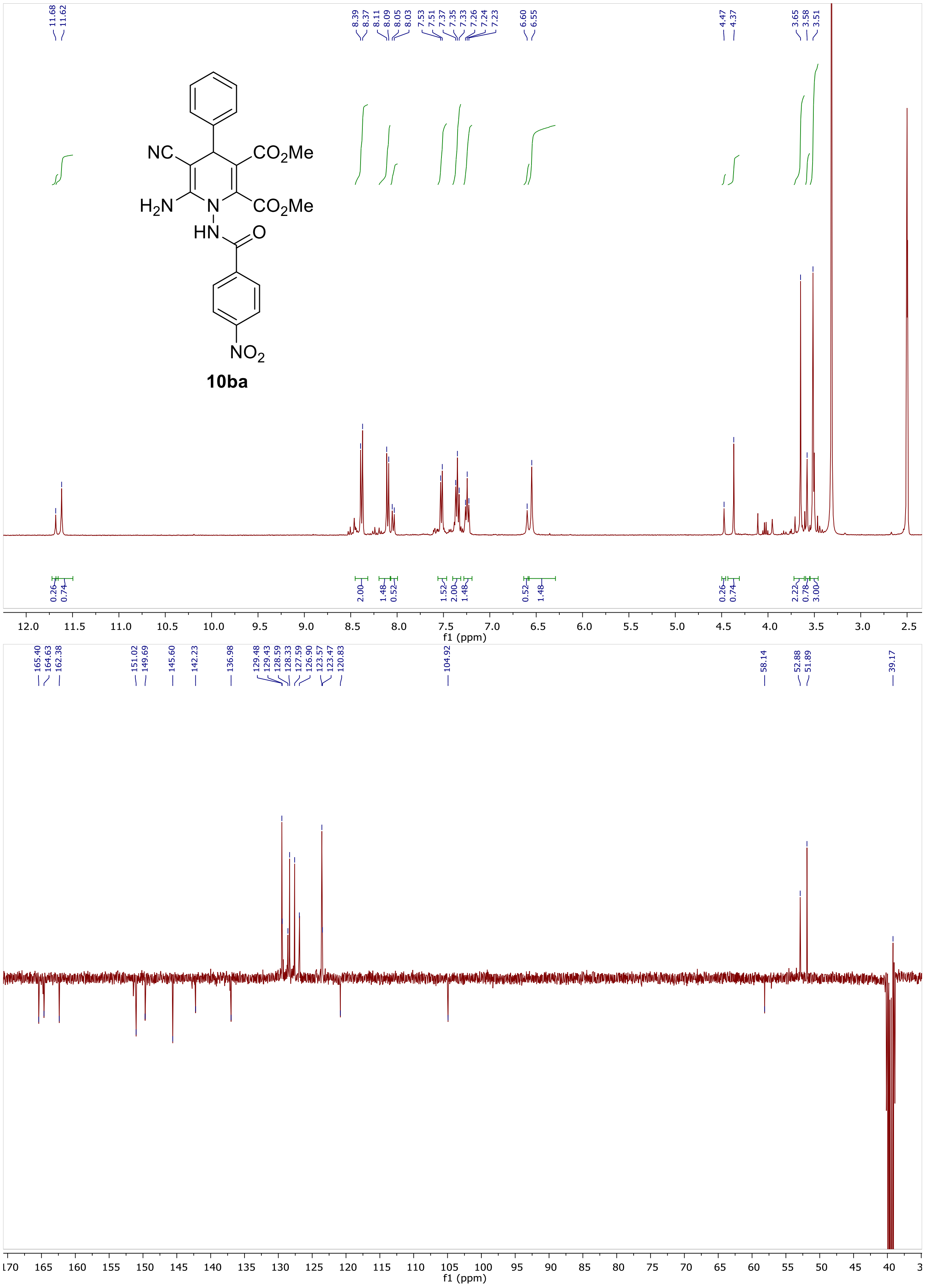

Figure S8. ^{1}H and ^{13}C-APT NMR spectra of dimethyl 6-amino-1-(4-chlorobenzamido)-5-cyano-4-phenyl-1,4-dihydropyridine-2,3-dicarboxylate (10ca)

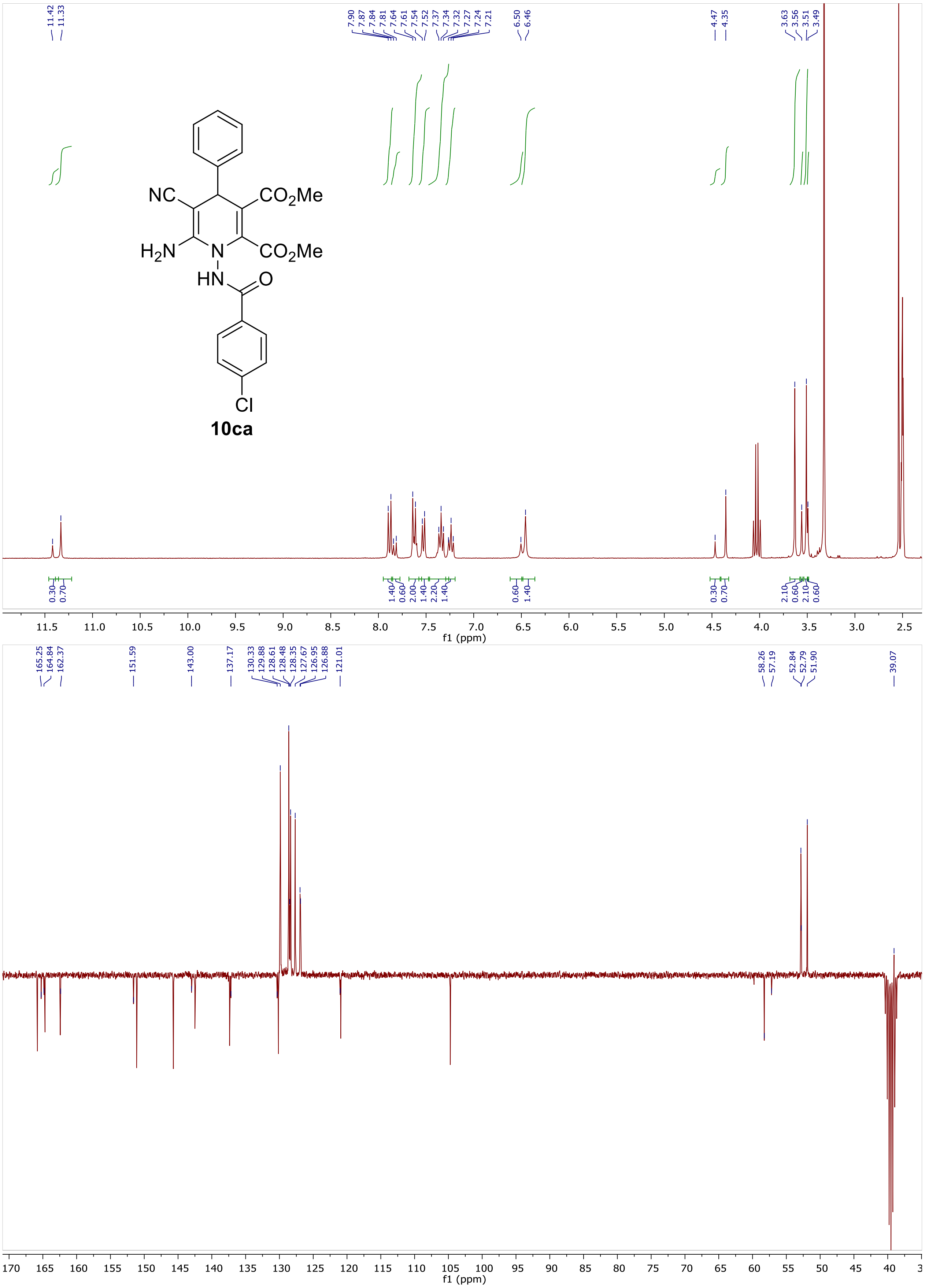

Figure S9. ^{1}H **and** ^{13}C**-APT NMR spectra of dimethyl 6-amino-1-(4-bromobenzamido)-5-cyano-4-phenyl-1,4-dihydropyridine-2,3-dicarboxylate (10da)**

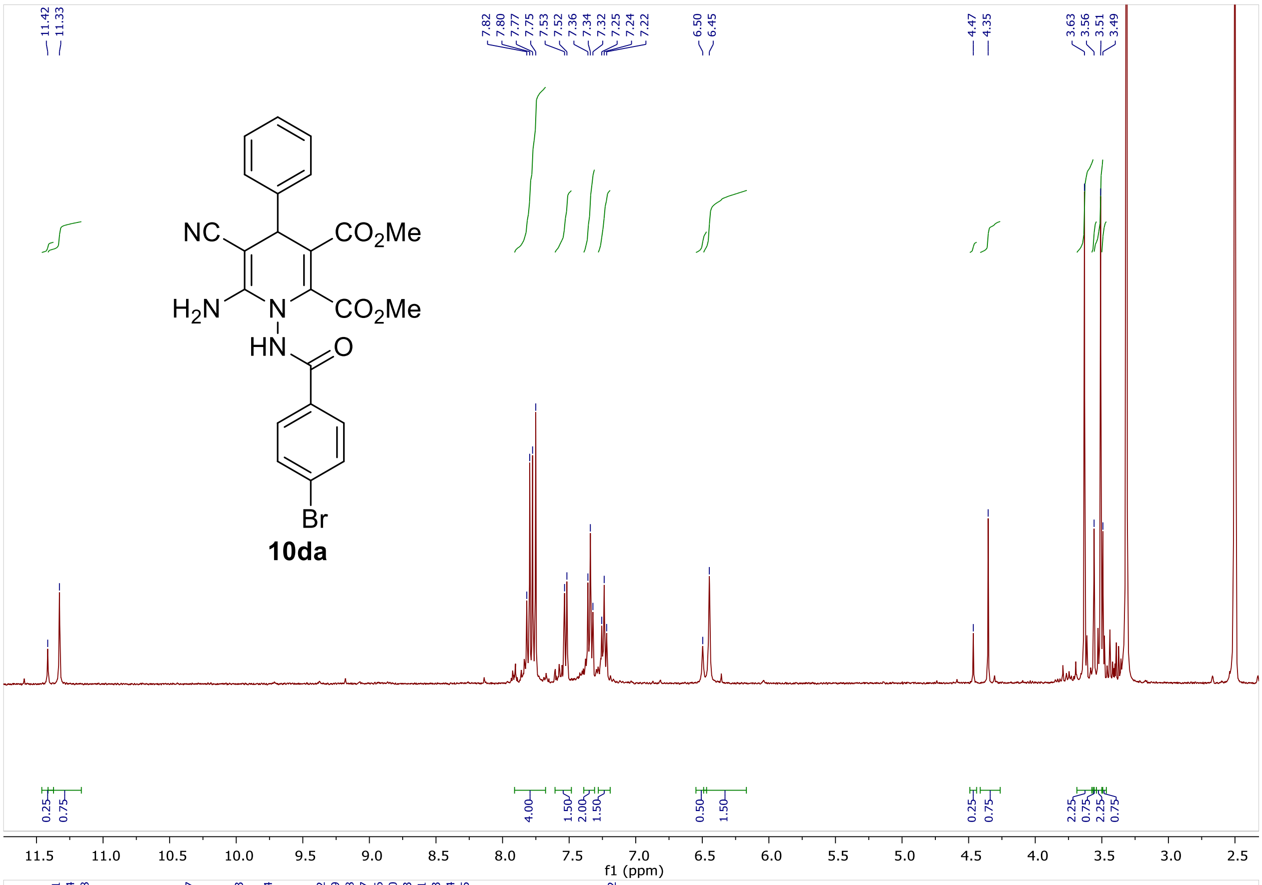

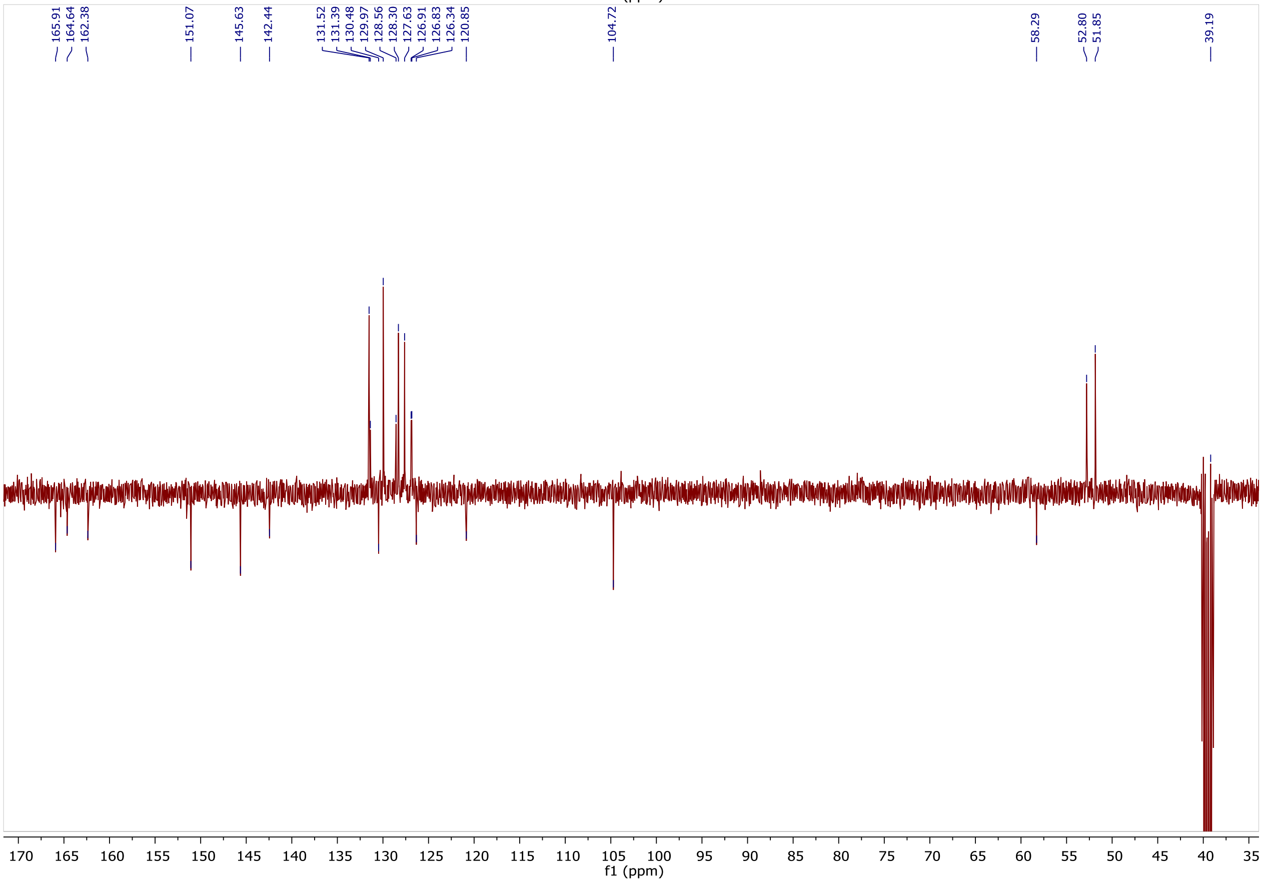

Figure S10. ^{1}H **and** ^{13}C**-APT NMR spectra of dimethyl 6-amino-1-(4-(tert-butyl)benzamido)-5-cyano-4-phenyl-1,4-dihydropyridine-2,3-dicarboxylate (10ea)**

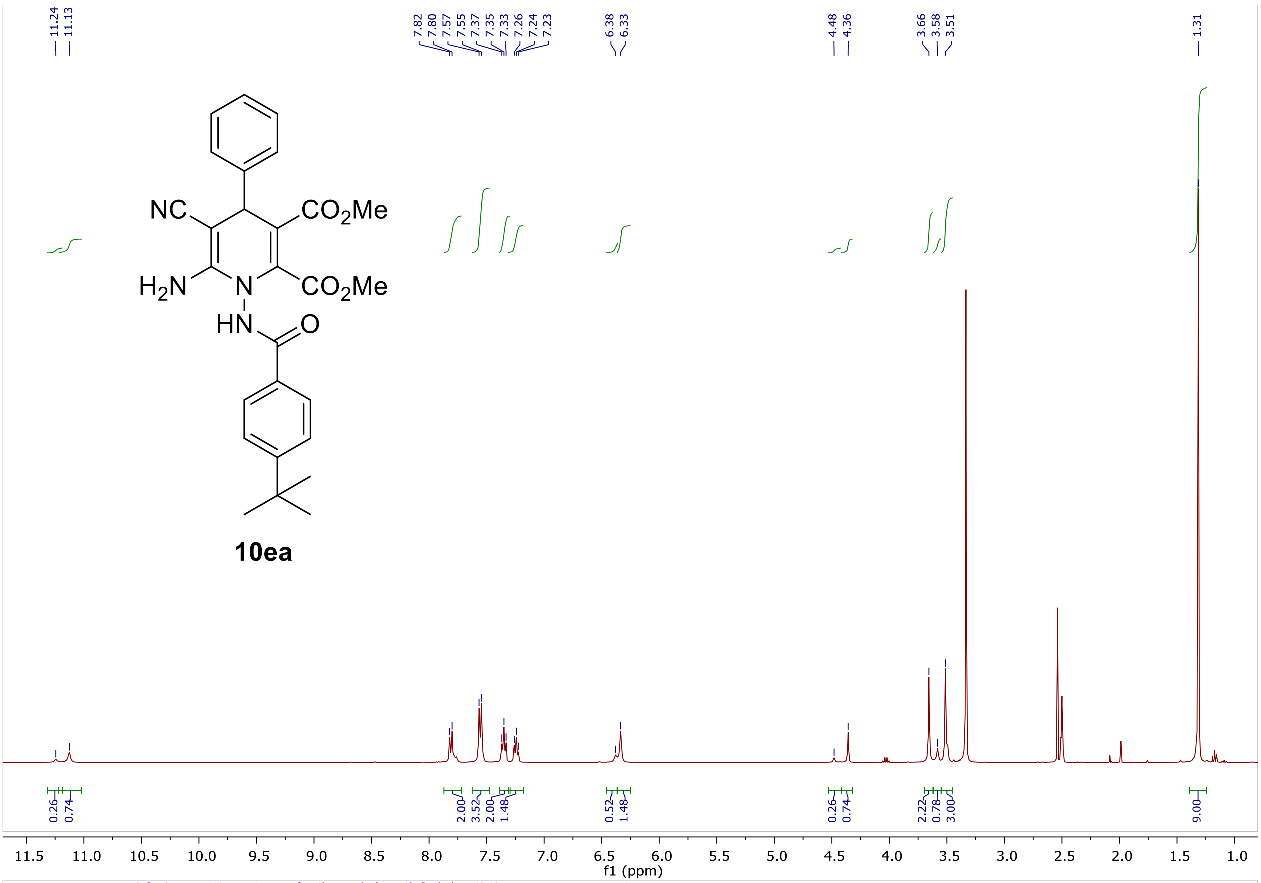

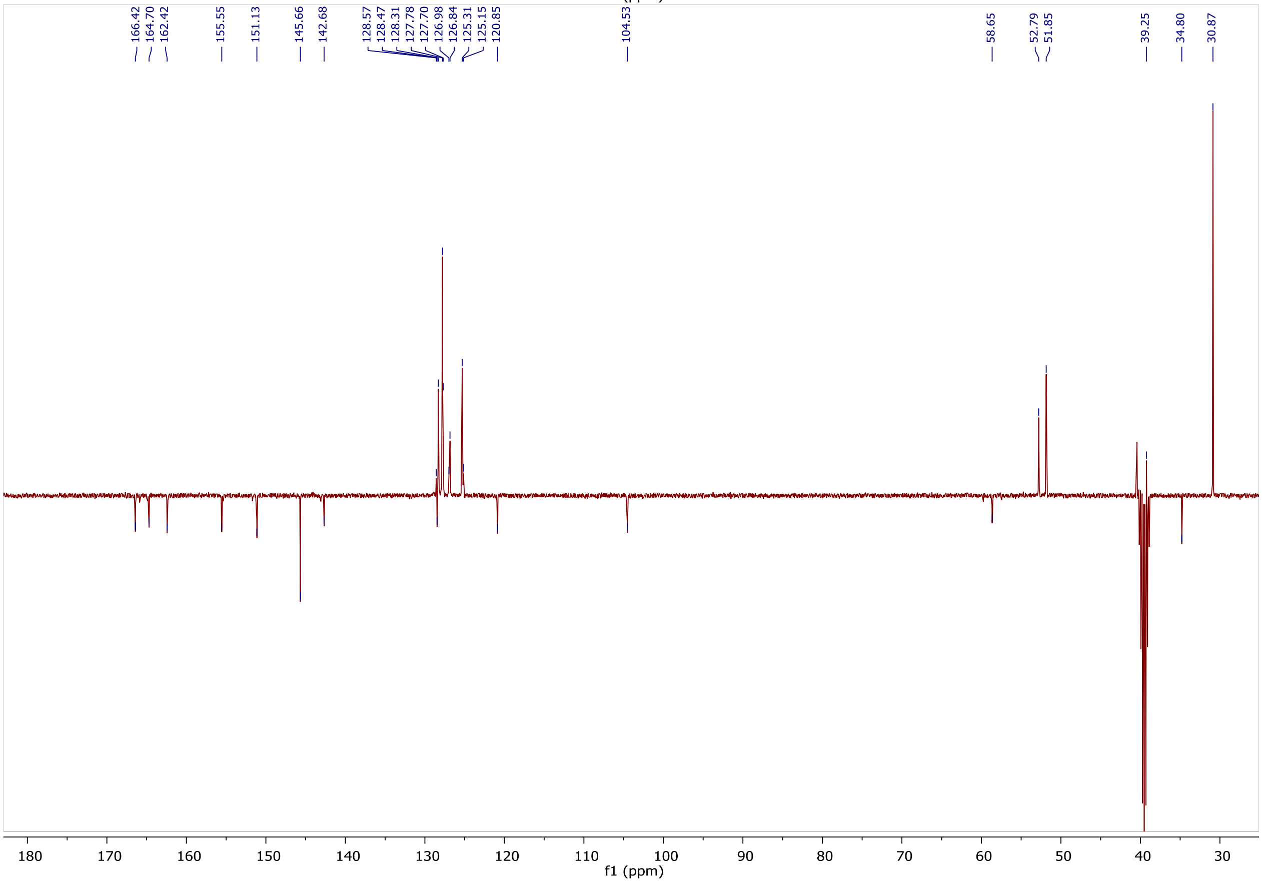

Figure S11. ^{1}H and ^{13}C-APT NMR spectra of dimethyl 6-amino-5-cyano-1-(4-methoxybenzamido)-4-phenyl-1,4-dihydropyridine-2,3-dicarboxylate (10fa)

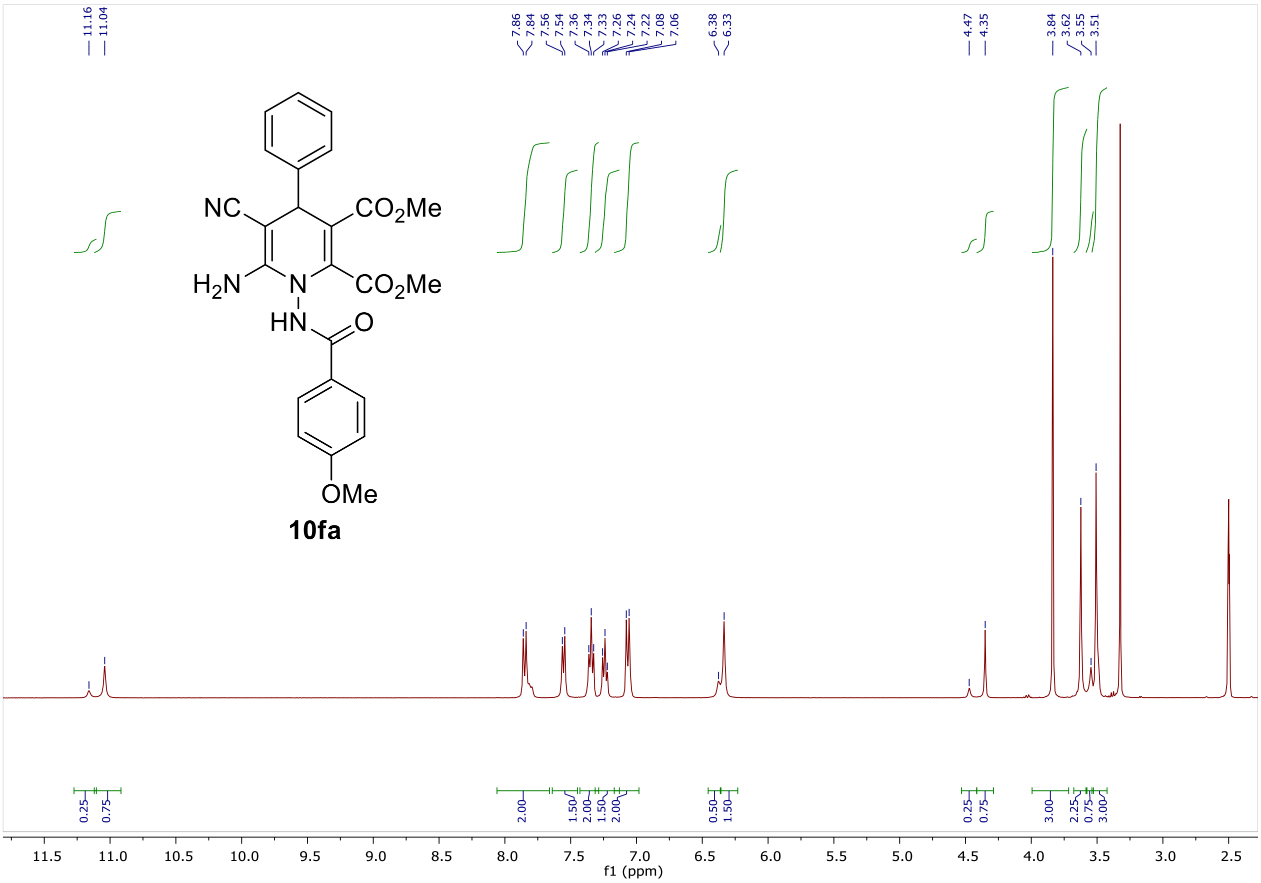

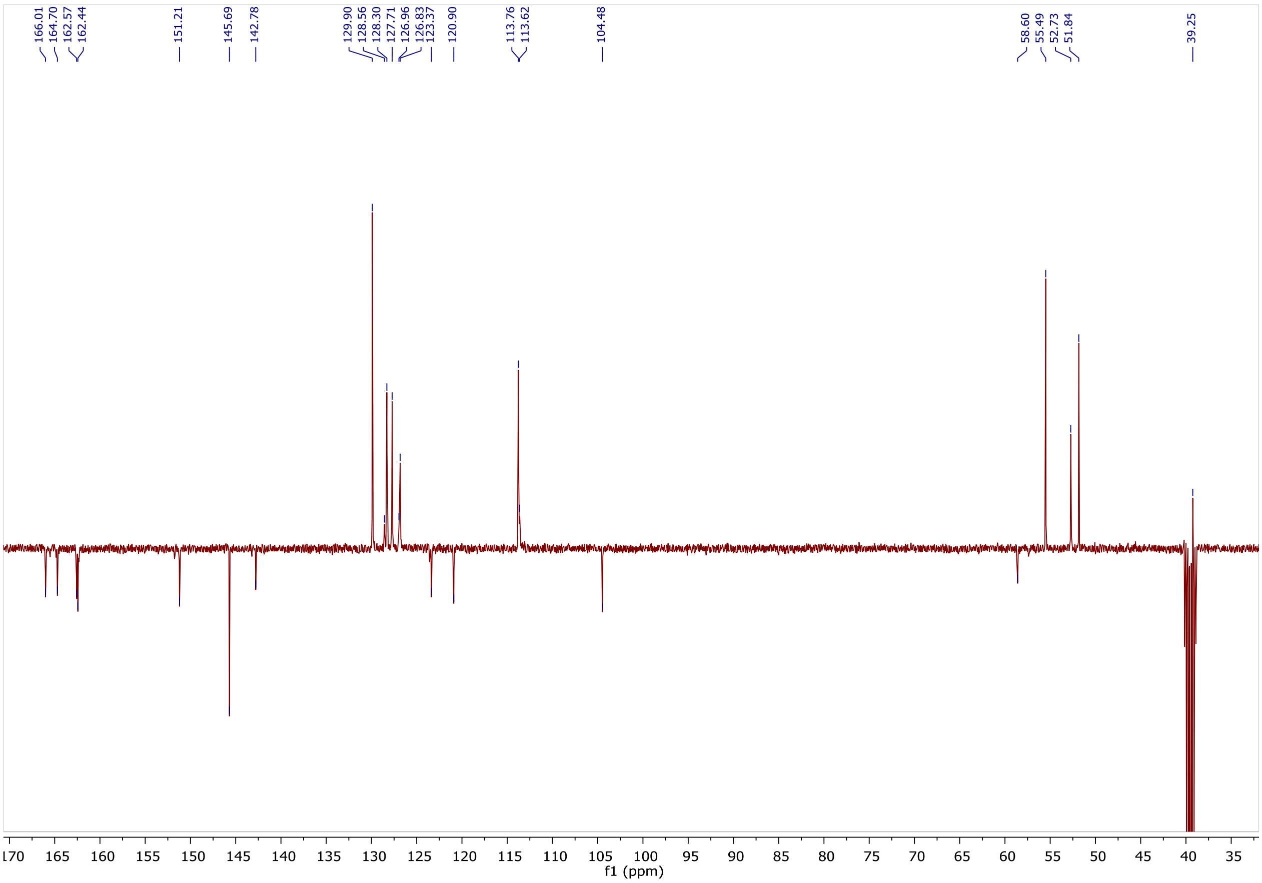

Figure S12. ^{1}H **and** ^{13}C**-APT NMR spectra of dimethyl 6-amino-1-benzamido-5-cyano-4-(4-nitrophenyl)-1,4-dihydropyridine-2,3-dicarboxylate (10ab)**

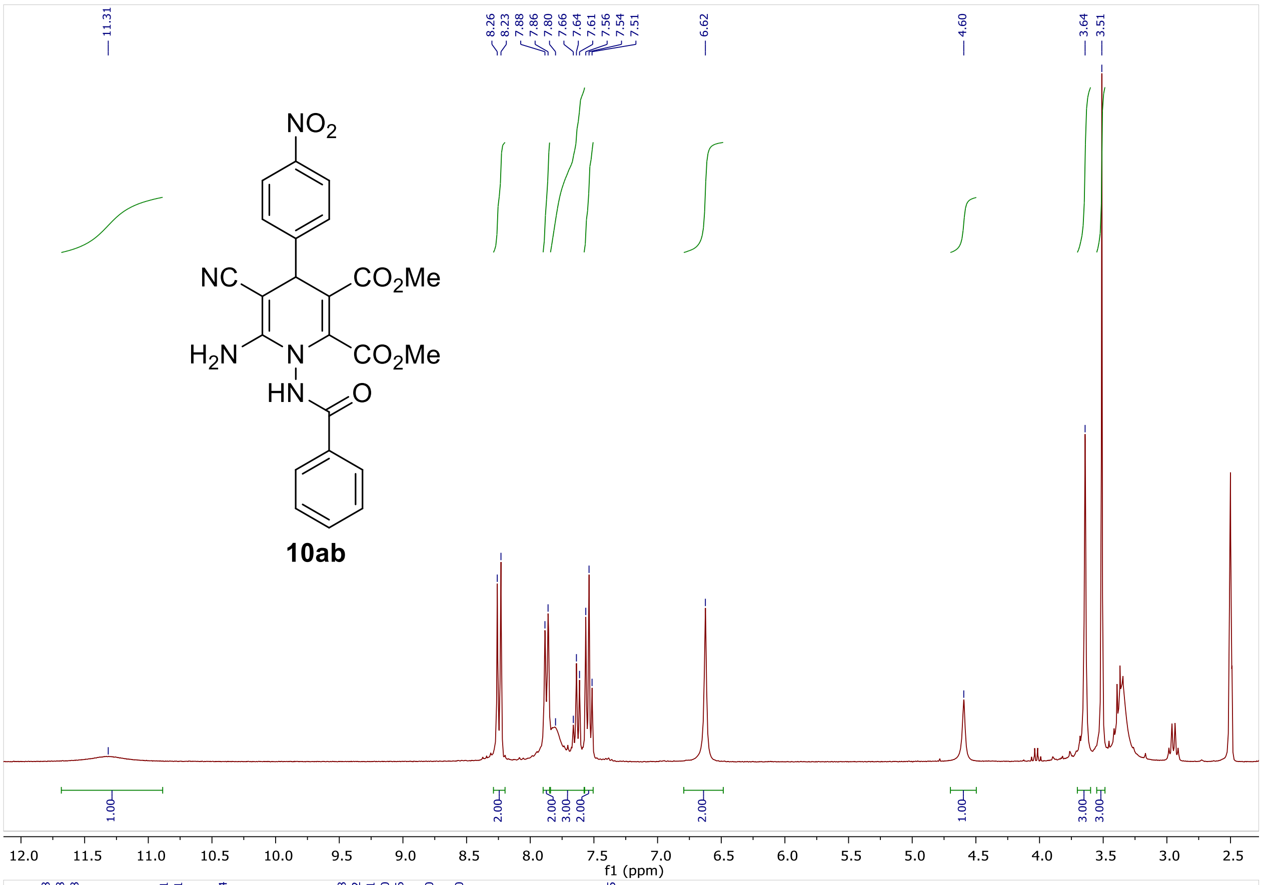

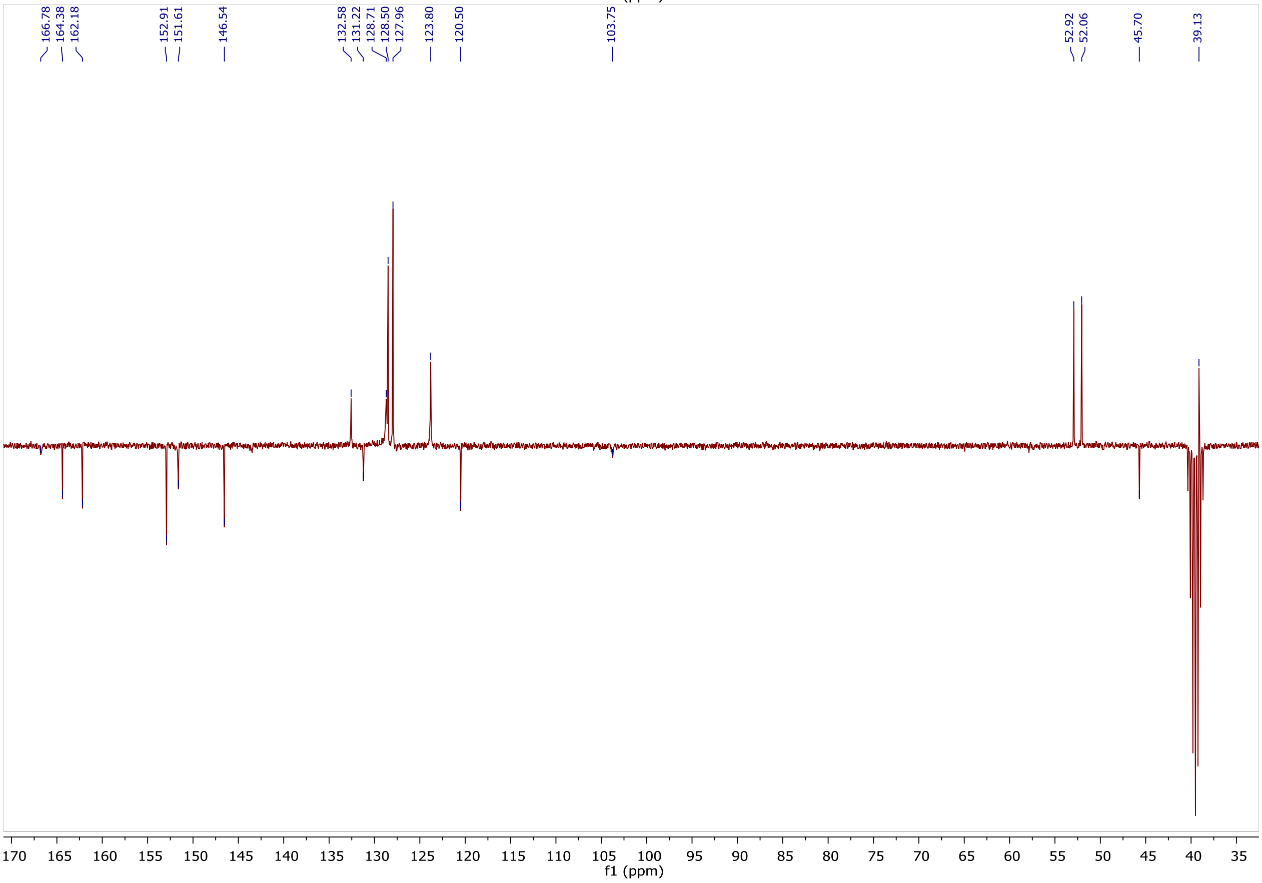

Figure S13. ^{1}H and ^{13}C-APT NMR spectra of dimethyl 6-amino-1-benzamido-4-(3-chlorophenyl)-5-cyano-1,4-dihydropyridine-2,3-dicarboxylate (10ad)

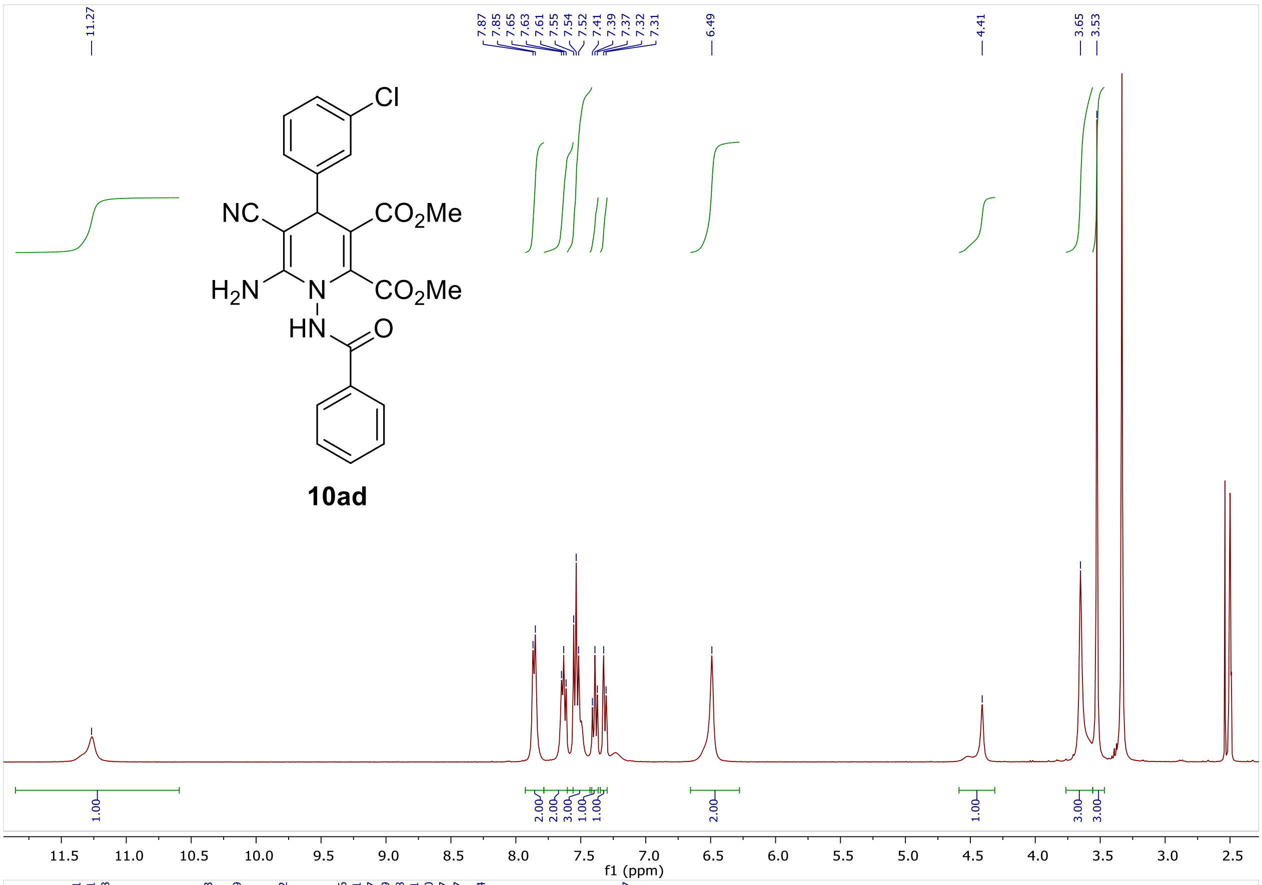

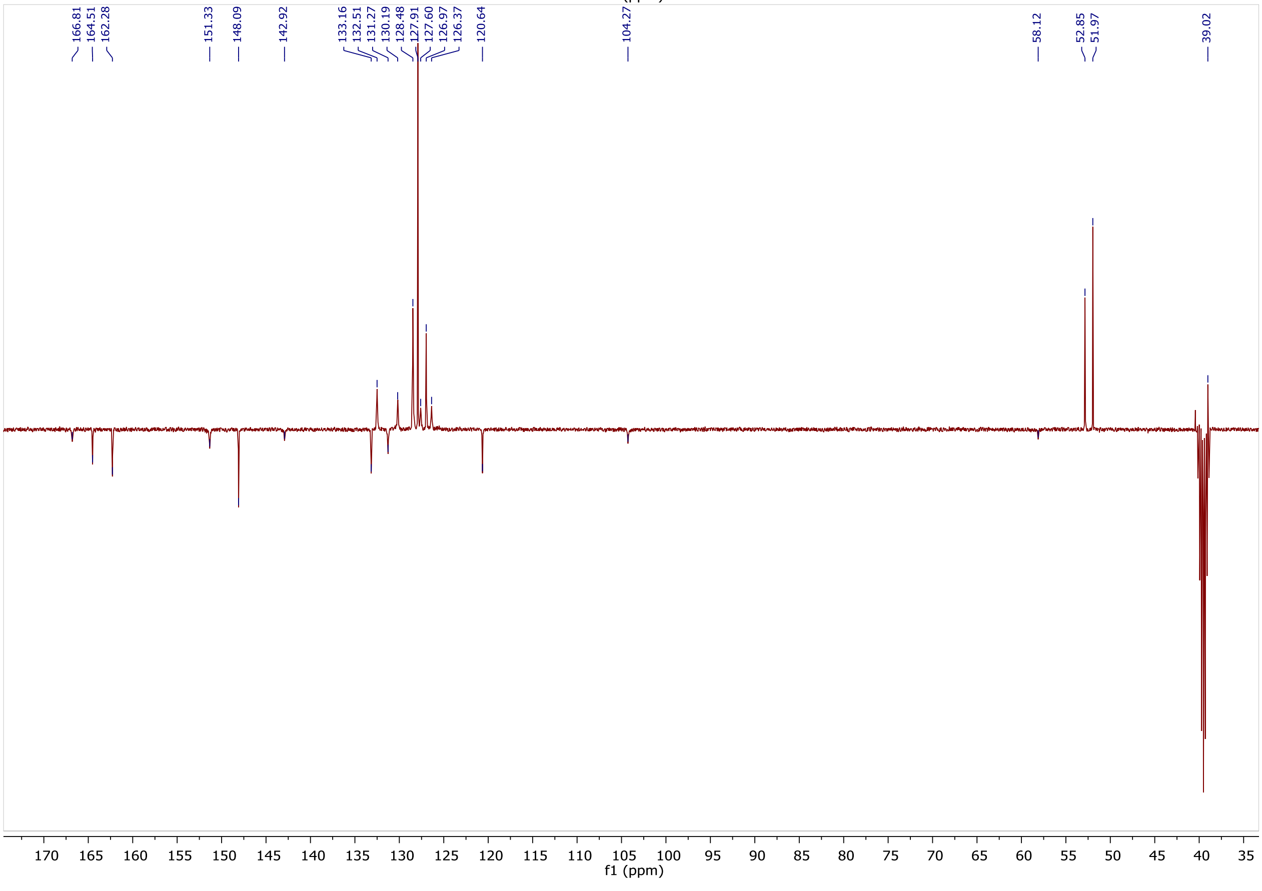

Figure S14. ^{1}H and ^{13}C-APT NMR spectra of dimethyl 6-amino-1-benzamido-5-cyano-4-(4-cyanophenyl)-1,4-dihydropyridine-2,3-dicarboxylate (10ag)

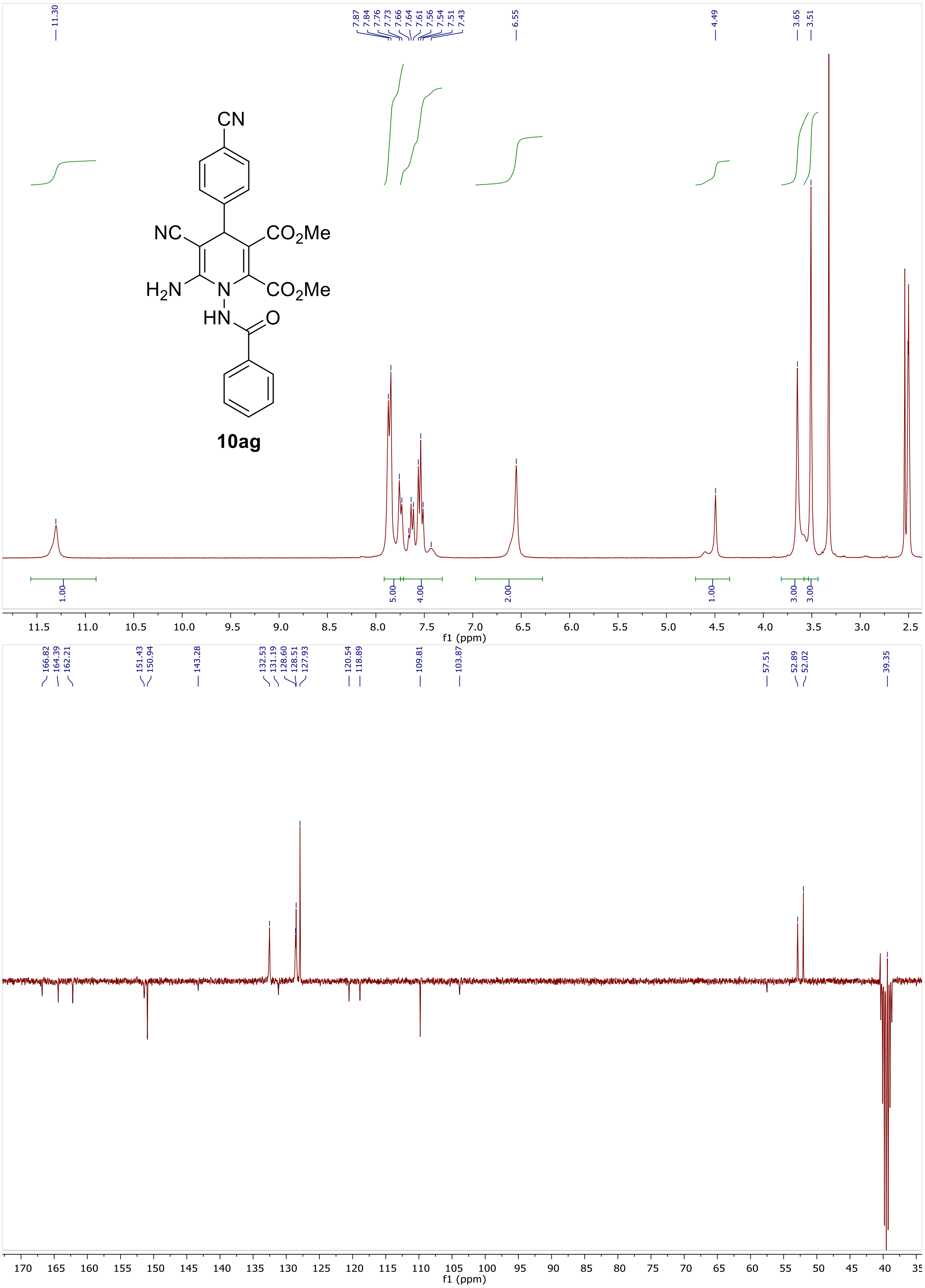

Figure S15. ^{1}H **and** ^{13}C**-APT NMR spectra of dimethyl 6-amino-1-benzamido-5-cyano-4-(naphthalen-1-yl)-1,4-dihydropyridine-2,3-dicarboxylate (10ah)**

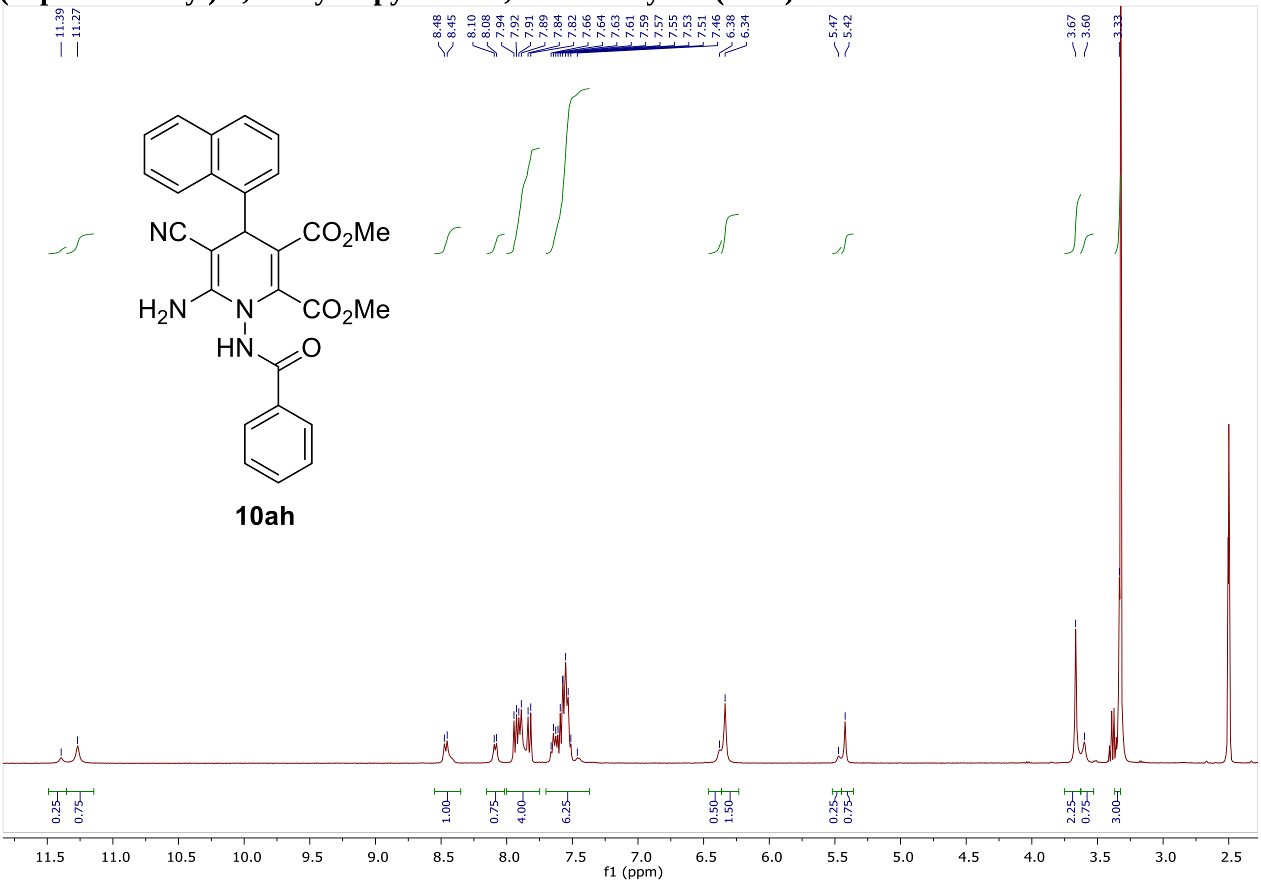

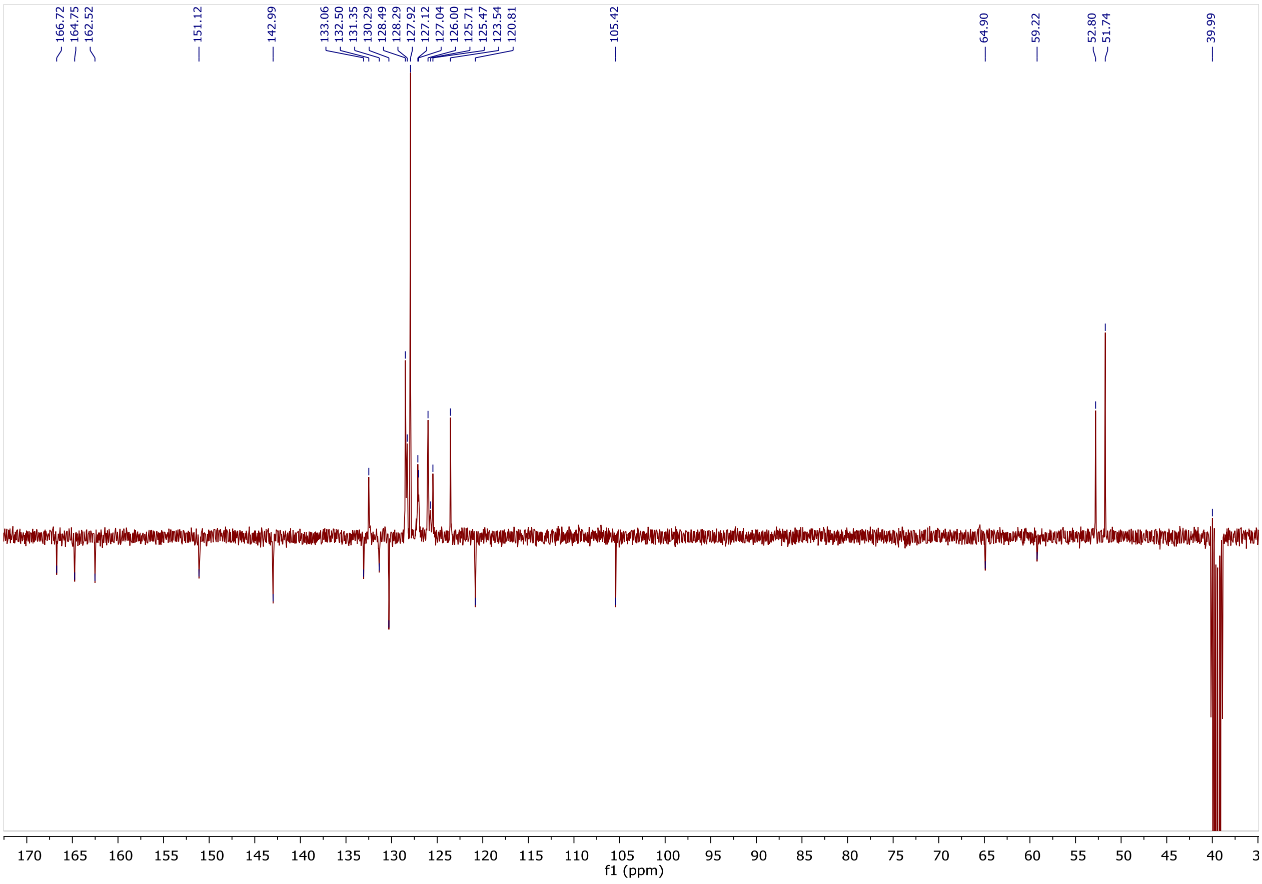

Figure S16. ^{1}H **and** ^{13}C**-APT NMR spectra of dimethyl 6-amino-1-benzamido-5-cyano-4-(4-methoxyphenyl)-1,4-dihydropyridine-2,3-dicarboxylate (10aj)**

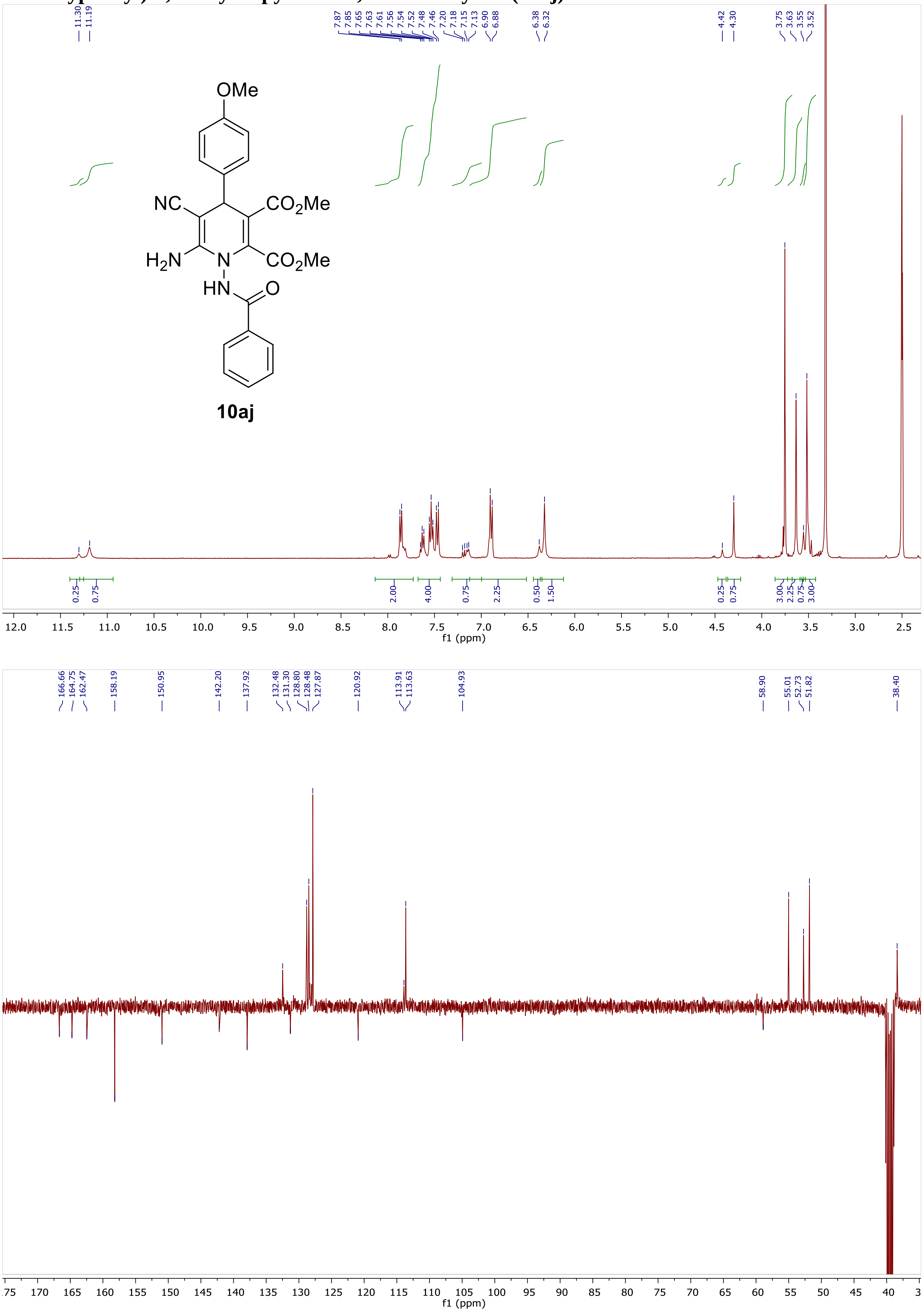

Figure S17. ^{1}H and ^{13}C-APT NMR spectra of dimethyl 6-amino-1-benzamido-5-cyano-4-(furan-2-yl)-1,4-dihydropyridine-2,3-dicarboxylate (10ak)

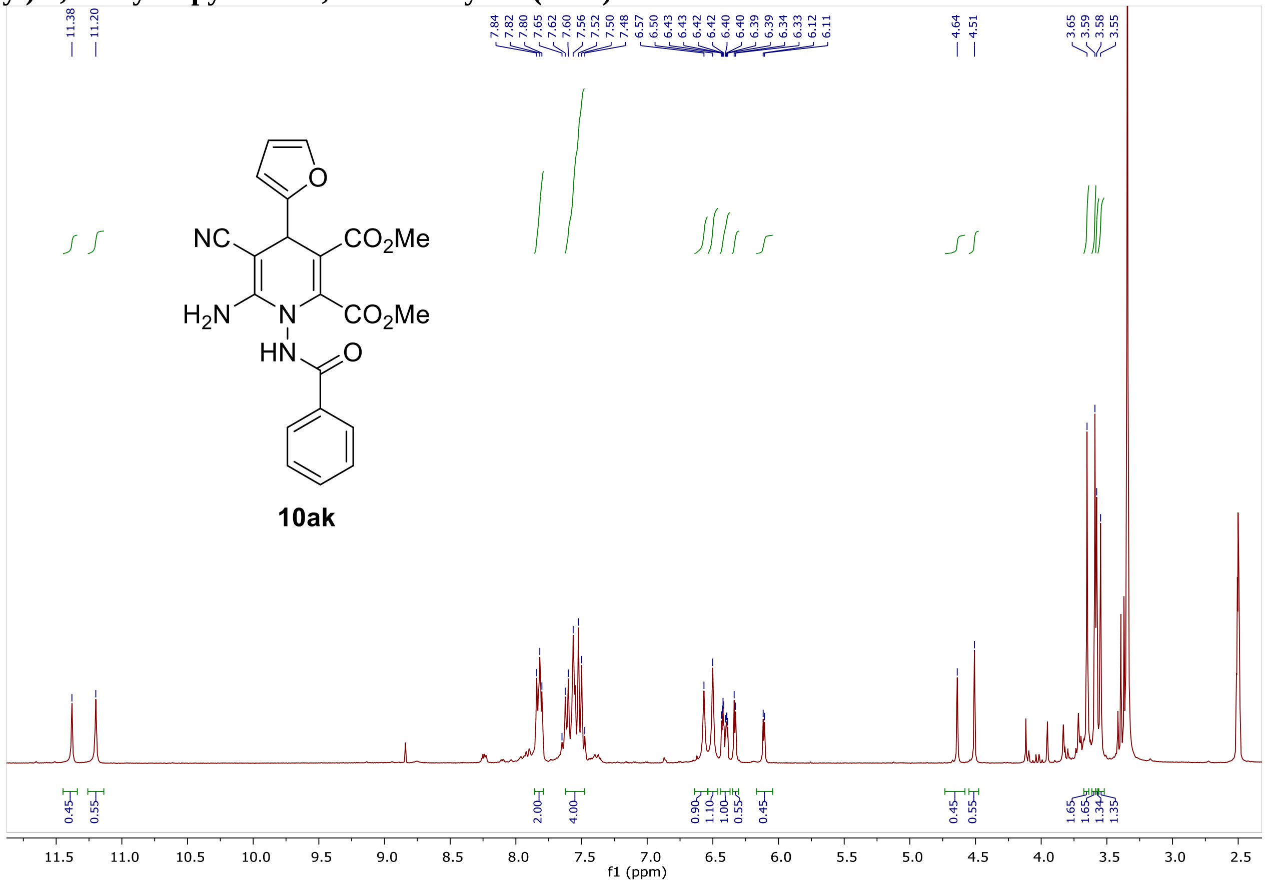

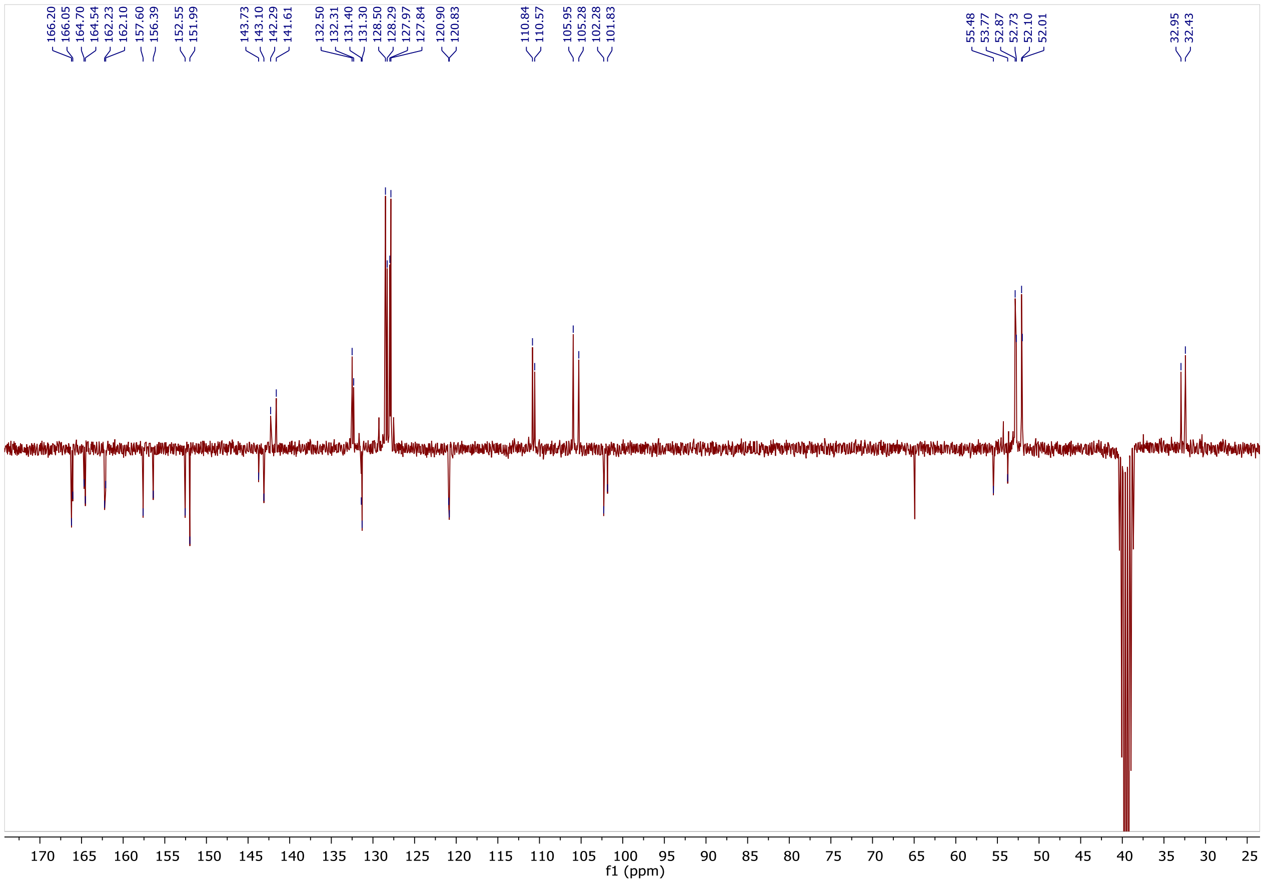

Figure S18. ^{1}H **and** ^{13}C**-APT NMR spectra of dimethyl 6-amino-1-benzamido-5-cyano-4-(thiophen-2-yl)-1,4-dihydropyridine-2,3-dicarboxylate (10al)**

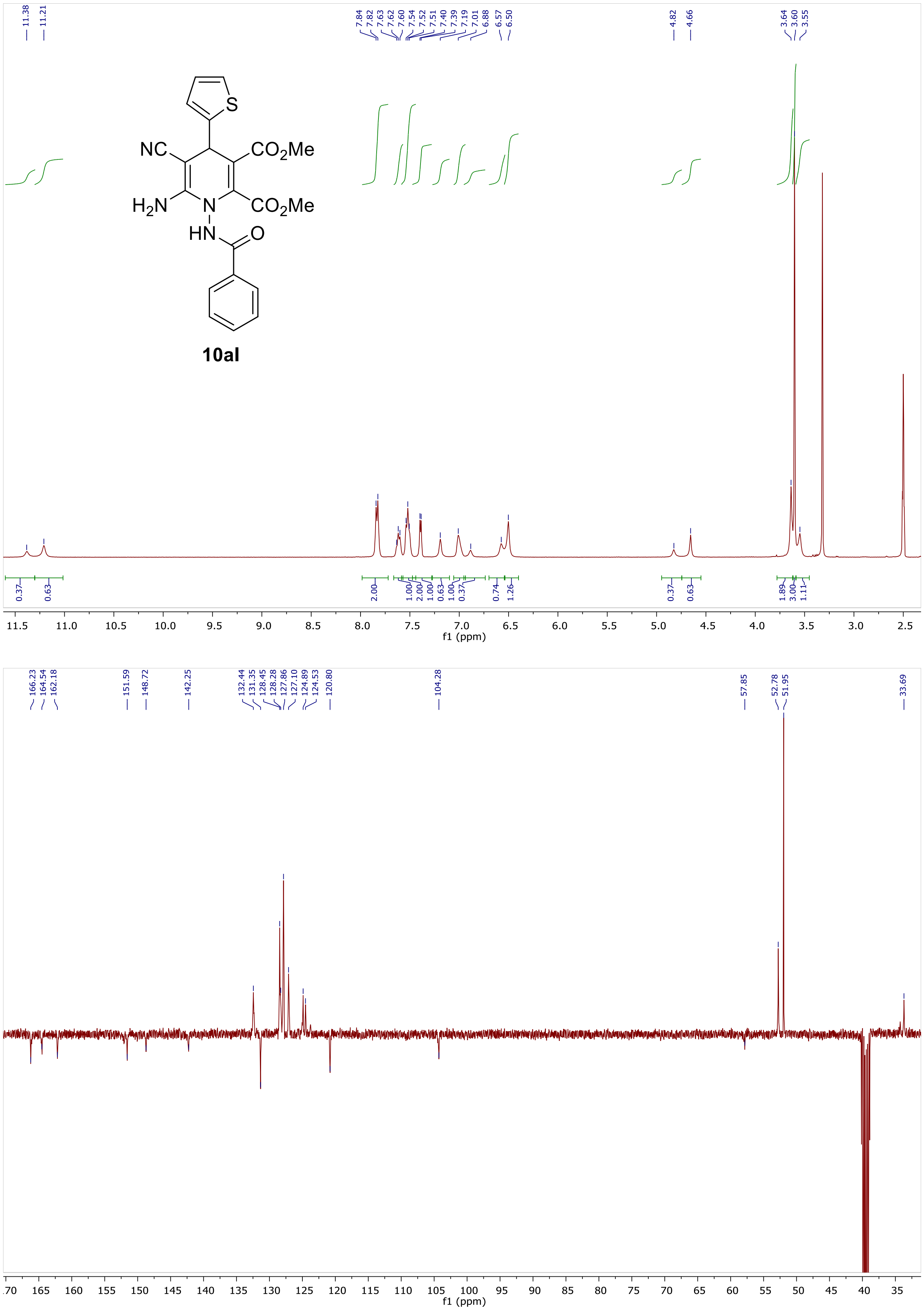

Figure S19. ^{1}H and ^{13}C-APT NMR spectra of dimethyl 6'-amino-1'-benzamido-5'-cyano-1',4'-dihydro-[3,4'-bipyridine]-2',3'-dicarboxylate (10am)

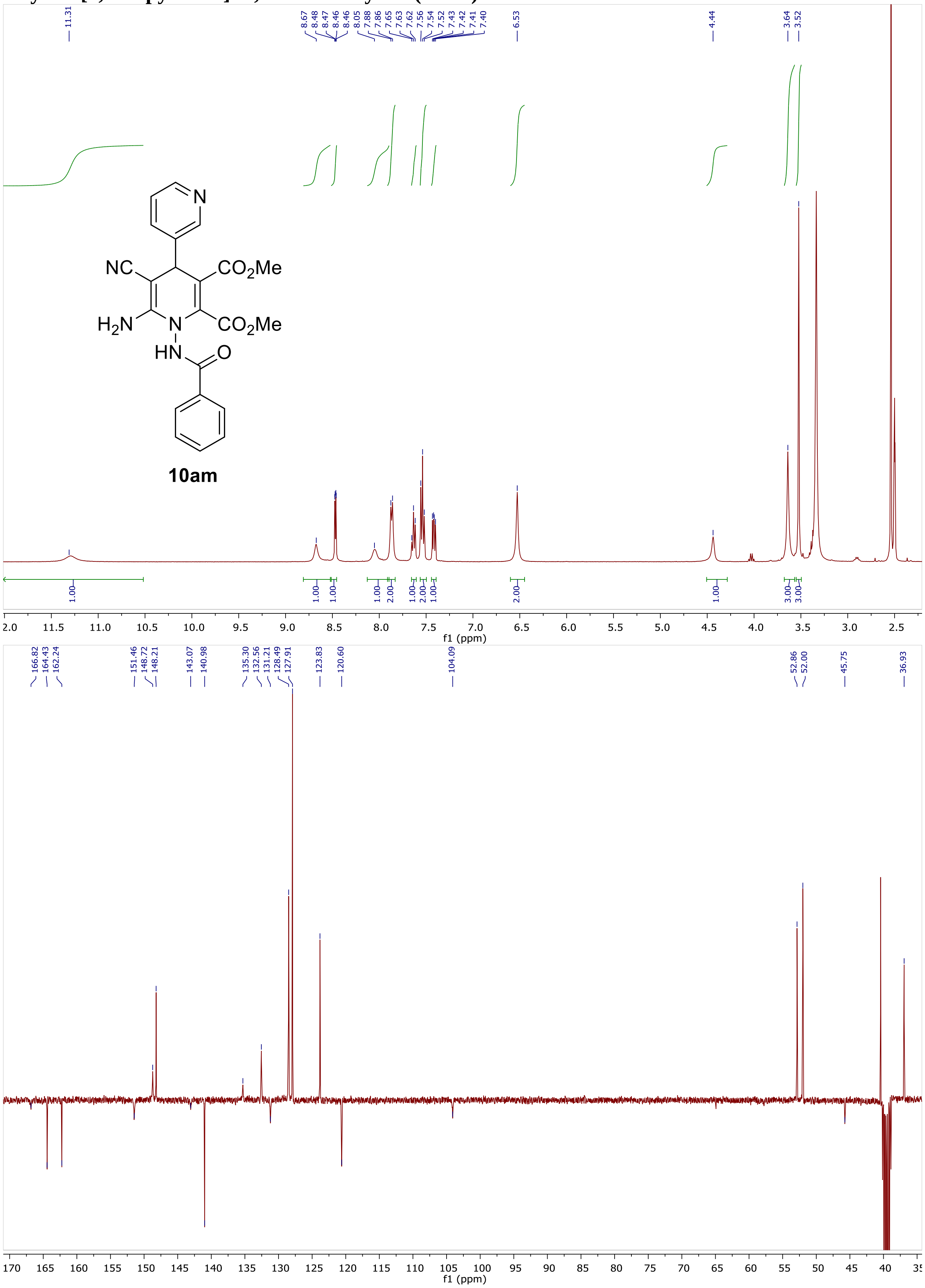

HPLC ANALYSIS OF 1,4-DIHYDROPYRIDINES 10

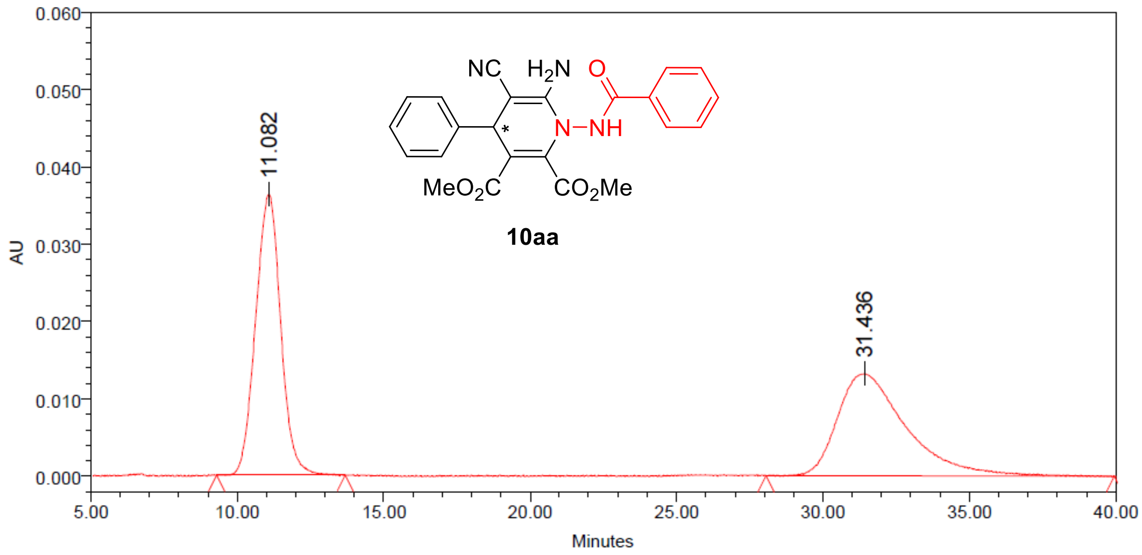

Processed Channel: PDA 337.7 nm

	Processed Channel	Retention Time (min)	Area	% Area	Height
1	PDA 337.7 nm	11.082	2158670	50.14	36296
2	PDA 337.7 nm	31.436	2146356	49.86	13164

Figure S20. Racemic mixture of **10aa**. Daicel Chiralpak IC column (*n*-hexane/*i*-PrOH = 70:30, flow rate 1 mL/min).

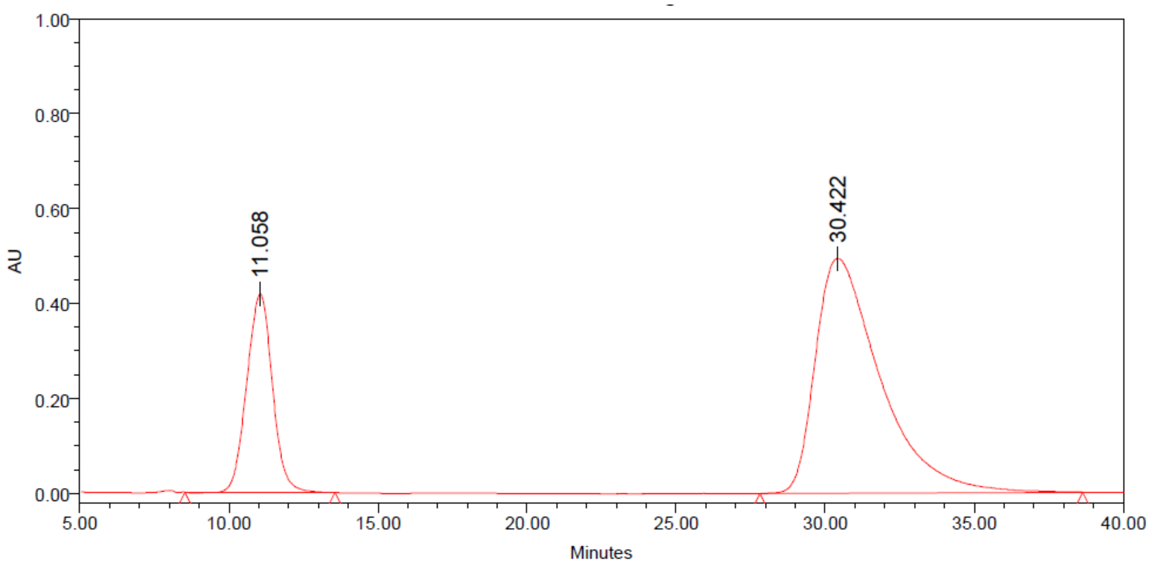

Processed Channel: PDA 237.7 nm

	Processed Channel	Retention Time (min)	Area	% Area	Height
1	PDA 237.7 nm	11.058	25161769	25.20	417936
2	PDA 237.7 nm	30.422	74681844	74.80	494807

Figure S21. Chiral sample of **dimethyl 6-amino-1-benzamido-5-cyano-4-phenyl-1,4-dihydropyridine-2,3-dicarboxylate (10aa)**.

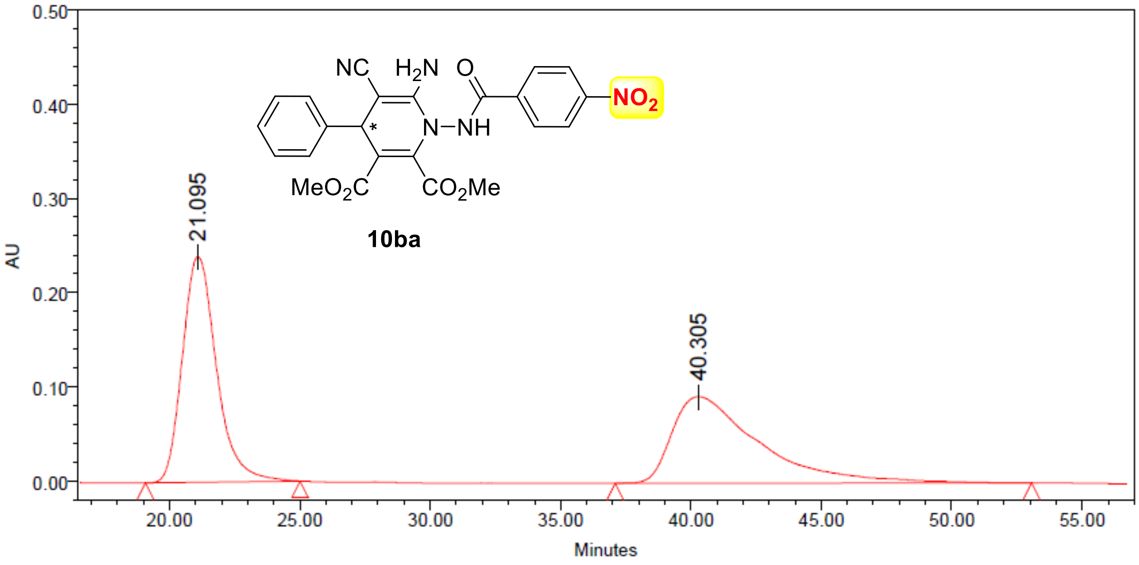

Processed Channel: PDA 249.6 nm

	Processed Channel	Retention Time (min)	Area	% Area	Height
1	PDA 249.6 nm	21.095	21776897	50.50	239114
2	PDA 249.6 nm	40.305	21347894	49.50	91917

Figure S22. Racemic mixture of **10ba**. Daicel Chiralpak IC column (*n*-hexane/*i*-PrOH = 70:30, flow rate 1 mL/min).

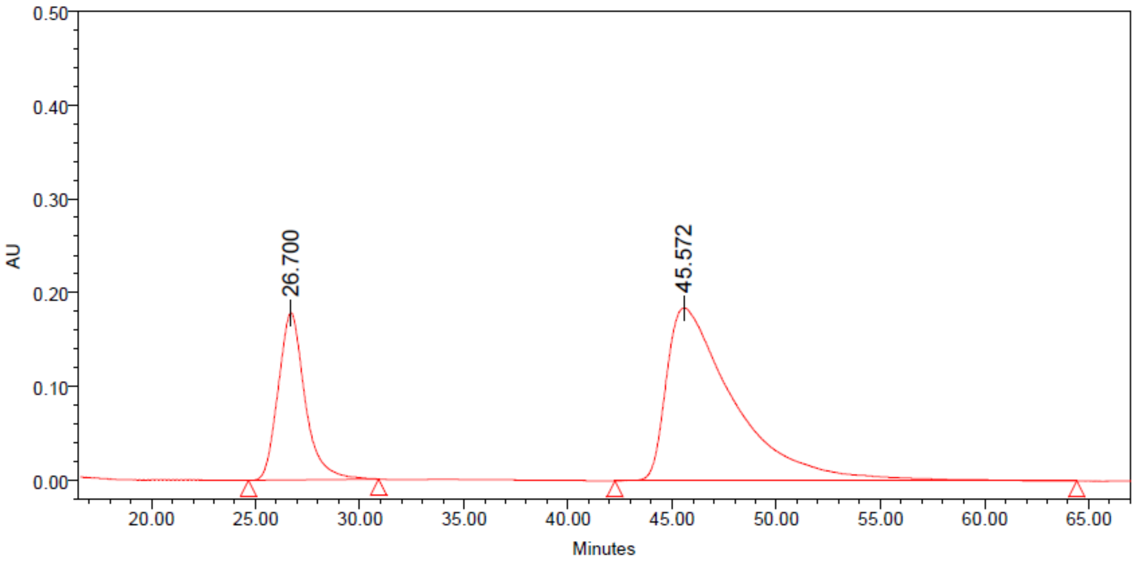

Processed Channel: PDA 249.6 nm

	Processed Channel	Retention Time (min)	Area	% Area	Height
1	PDA 249.6 nm	26.700	15938249	27.60	178196
2	PDA 249.6 nm	45.572	41807602	72.40	184381

Figure S23. Chiral sample of **dimethyl 6-amino-5-cyano-1-(4-nitrobenzamido)-4-phenyl-1,4-dihydropyridine-2,3-dicarboxylate (10ba).**

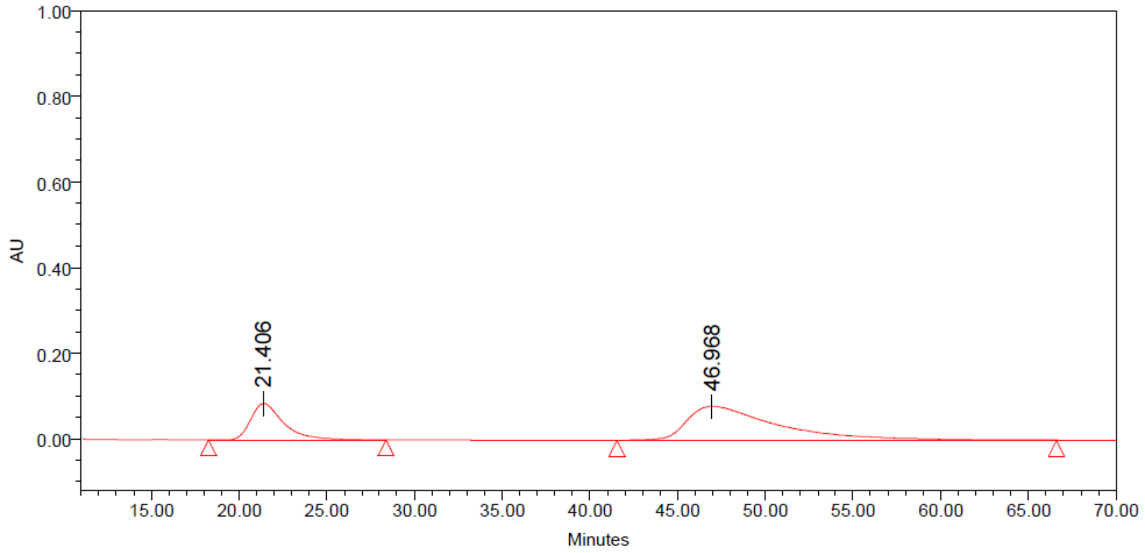

Processed Channel: PDA 241.8 nm

	Processed Channel	Retention Time (min)	Area	% Area	Height
1	PDA 241.8 nm	21.406	11105207	28.00	85216
2	PDA 241.8 nm	46.968	28562492	72.00	79410

Figure S24. Chiral sample of **dimethyl 6-amino-5-cyano-1-(4-nitrobenzamido)-4-phenyl-1,4-dihydropyridine-2,3-dicarboxylate (10ba)** injected 3 months later.

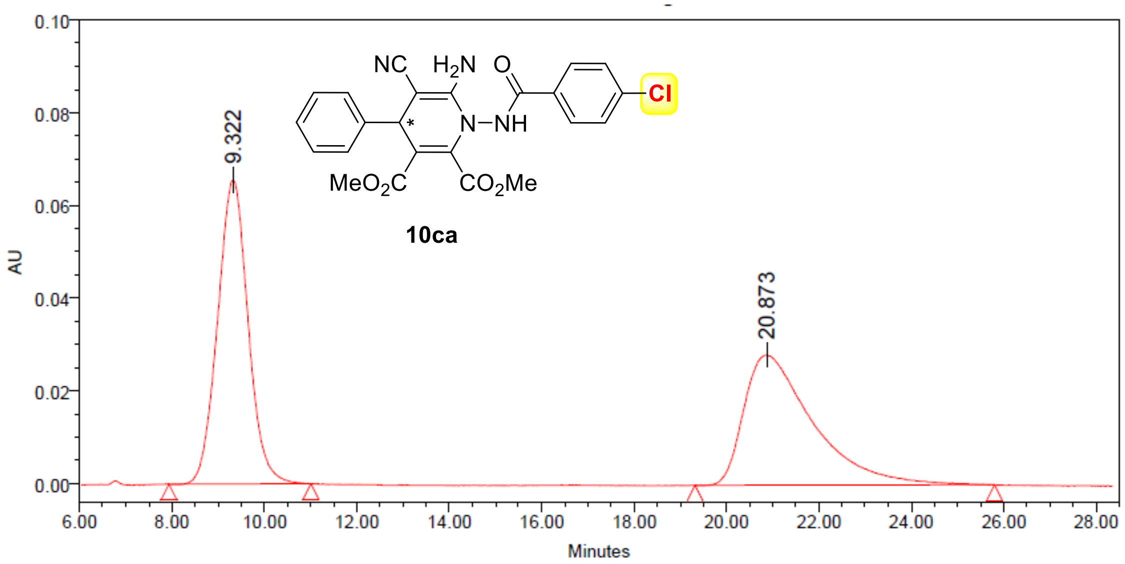

Processed Channel: PDA 310.5 nm

	Processed Channel	Retention Time (min)	Area	% Area	Height
1	PDA 310.5 nm	9.322	3013526	50.80	65614
2	PDA 310.5 nm	20.873	2918428	49.20	28078

Figure S25. Racemic mixture of **10ca**. Daicel Chiralpak IC column (*n*-hexane/*i*-PrOH = 70:30, flow rate 1 mL/min).

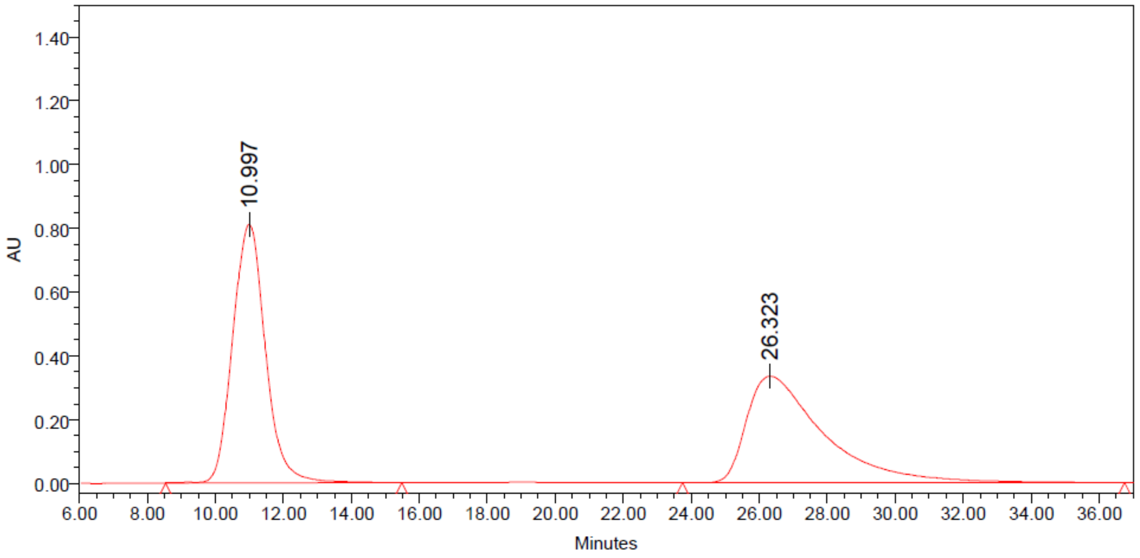

Processed Channel: PDA 254.0 nm

	Processed Channel	Retention Time (min)	Area	% Area	Height
1	PDA 254.0 nm	10.997	54334077	50.83	810319
2	PDA 254.0 nm	26.323	52551482	49.17	334036

Figure S26. Racemic mixture of **10ca**. Daicel Chiralpak IC column (*n*-hexane/*i*-PrOH = 70:30, flow rate 1 mL/min) injected 3 months later.

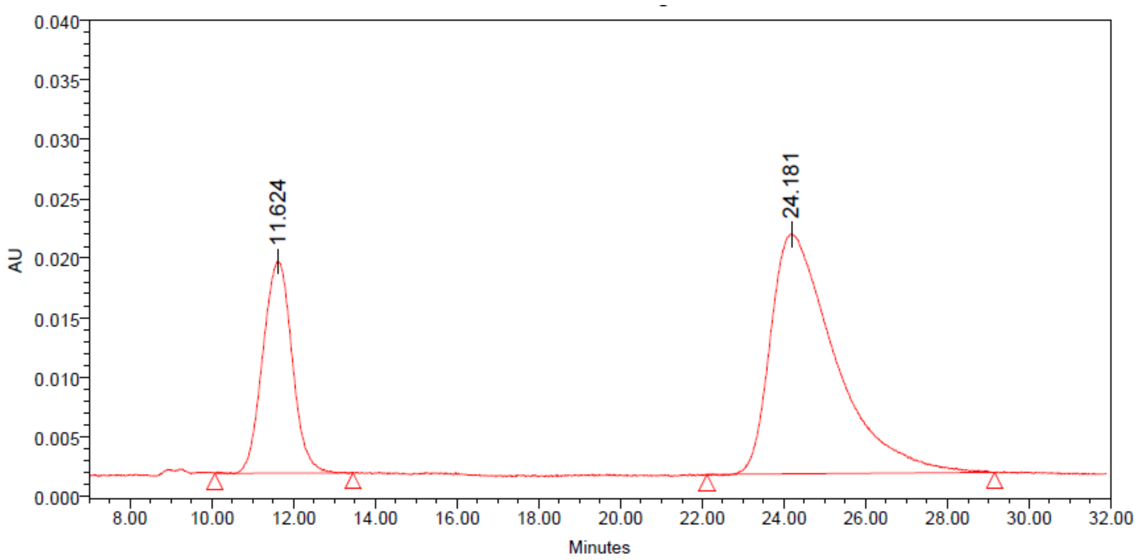

Processed Channel: PDA 310.5 nm

	Processed Channel	Retention Time (min)	Area	% Area	Height
1	PDA 310.5 nm	11.624	885053	28.54	17794
2	PDA 310.5 nm	24.181	2216192	71.46	20151

Figure S27. Chiral sample of **dimethyl 6-amino-1-(4-chlorobenzamido)-5-cyano-4-phenyl-1,4-dihydropyridine-2,3-dicarboxylate (10ca)**.

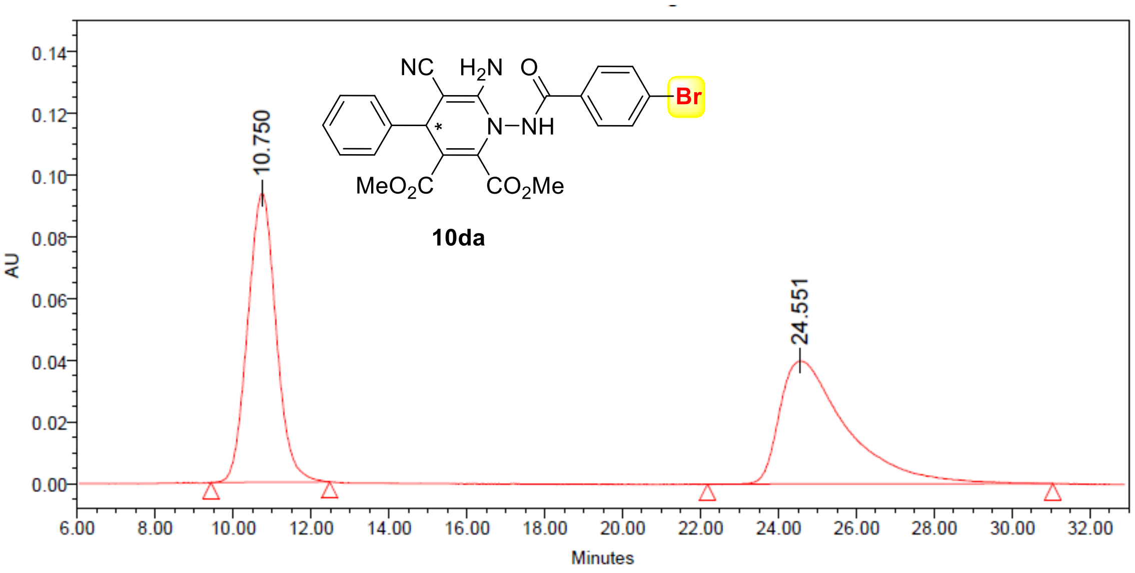

Processed Channel: PDA 330.0 nm

	Processed Channel	Retention Time (min)	Area	% Area	Height
1	PDA 330.0 nm	10.750	4738746	49.95	93627
2	PDA 330.0 nm	24.551	4747889	50.05	39905

Figure S28. Racemic mixture of **10da**. Daicel Chiralpak IC column (*n*-hexane/*i*-PrOH = 70:30, flow rate 1 mL/min).

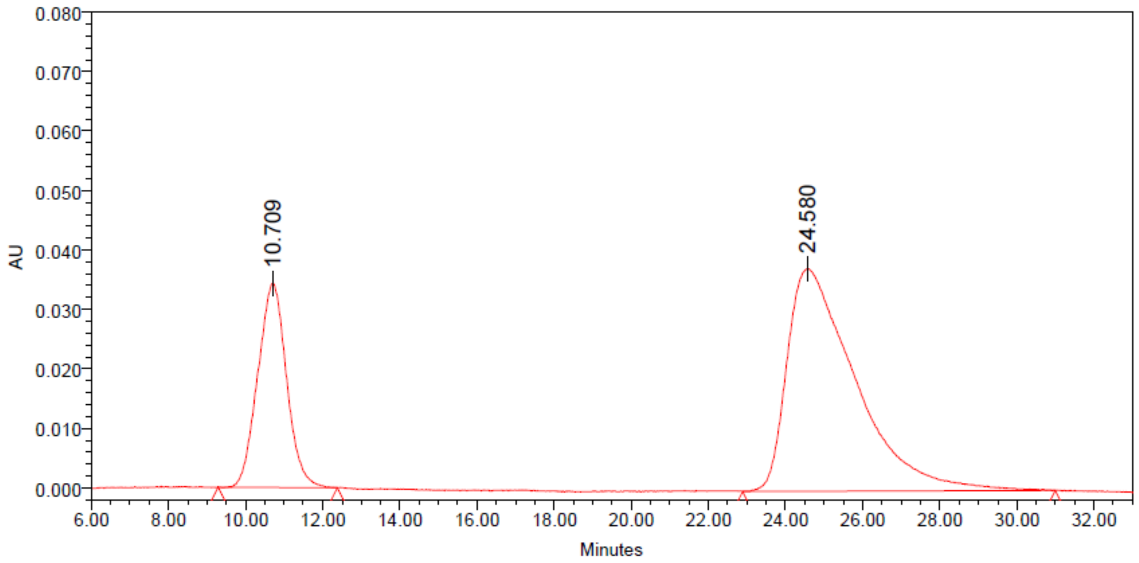

Processed Channel: PDA 330.0 nm

	Processed Channel	Retention Time (min)	Area	% Area	Height
1	PDA 330.0 nm	10.709	1776175	27.90	34259
2	PDA 330.0 nm	24.580	4590529	72.10	37376

Figure S29. Chiral sample of **dimethyl 6-amino-1-(4-bromobenzamido)-5-cyano-4-phenyl-1,4-dihydropyridine-2,3-dicarboxylate (10da)**.

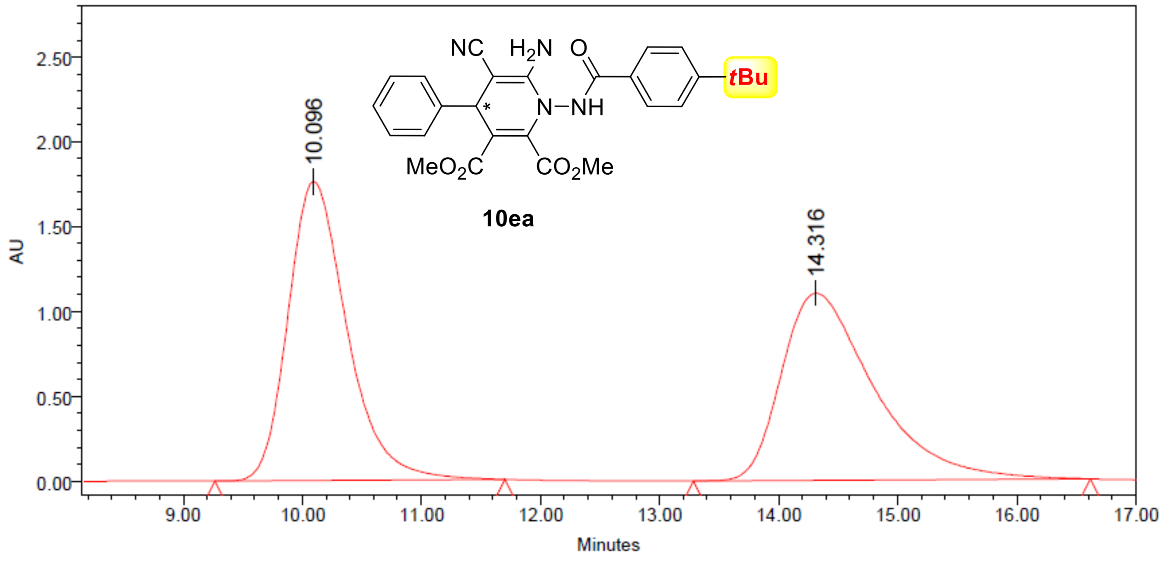

Processed Channel: PDA 242.5 nm

	Processed Channel	Retention Time (min)	Area	% Area	Height
1	PDA 242.5 nm	10.096	60689125	50.51	1761519
2	PDA 242.5 nm	14.316	59471736	49.49	1101201

Figure S30. Racemic mixture of **10ea**. Daicel Chiralpak IC column (*n*-hexane/*i*-PrOH = 70:30, flow rate 1 mL/min).

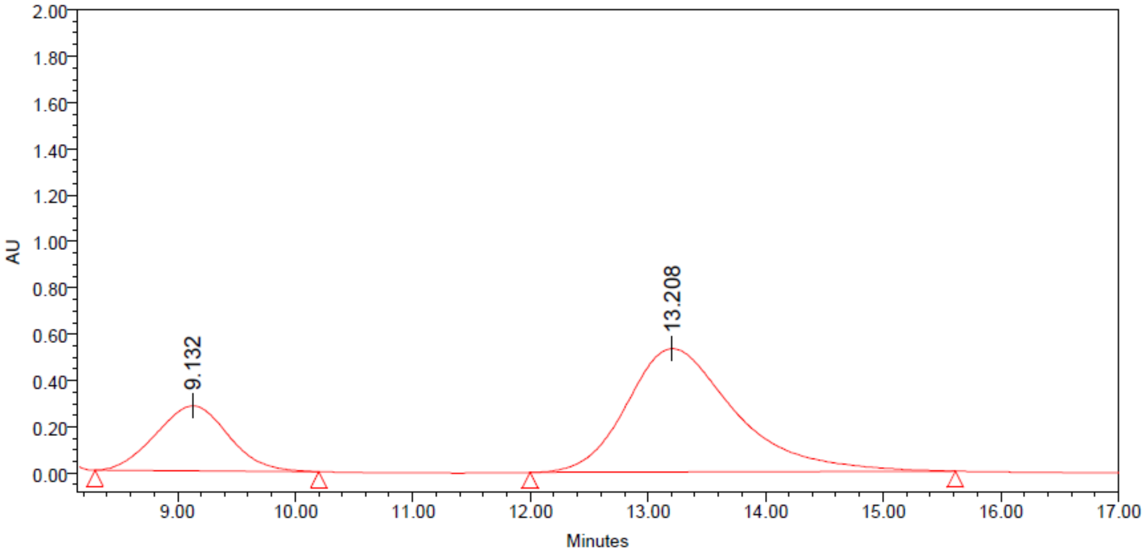

Processed Channel: PDA 242.5 nm

	Processed Channel	Retention Time (min)	Area	% Area	Height
1	PDA 242.5 nm	9.132	12055280	26.06	280193
2	PDA 242.5 nm	13.208	34205034	73.94	533262

Figure S31. Chiral sample of **dimethyl 6-amino-1-(4-(*tert*-butyl)benzamido)-5-cyano-4-phenyl-1,4-dihydropyridine-2,3-dicarboxylate (10ea)**.

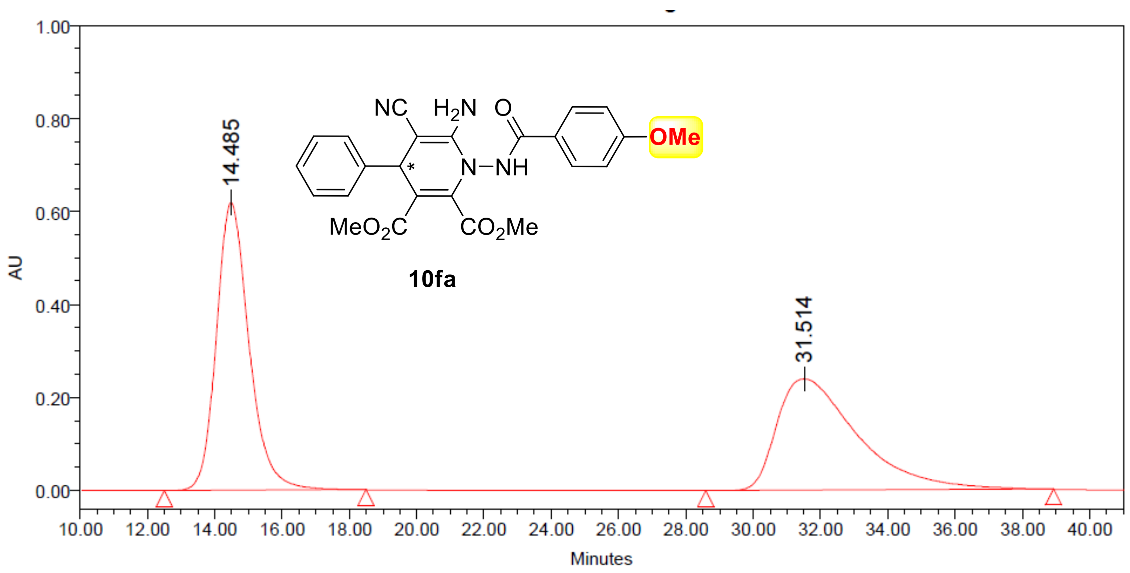

Processed Channel: PDA 253.2 nm

	Processed Channel	Retention Time (min)	Area	% Area	Height
1	PDA 253.2 nm	14.485	41736937	51.06	618777
2	PDA 253.2 nm	31.514	39997758	48.94	239602

Figure S32. Racemic mixture of **10fa**. Daicel Chiralpak IC column (*n*-hexane/*i*-PrOH = 70:30, flow rate 1 mL/min) injected 3 months later.

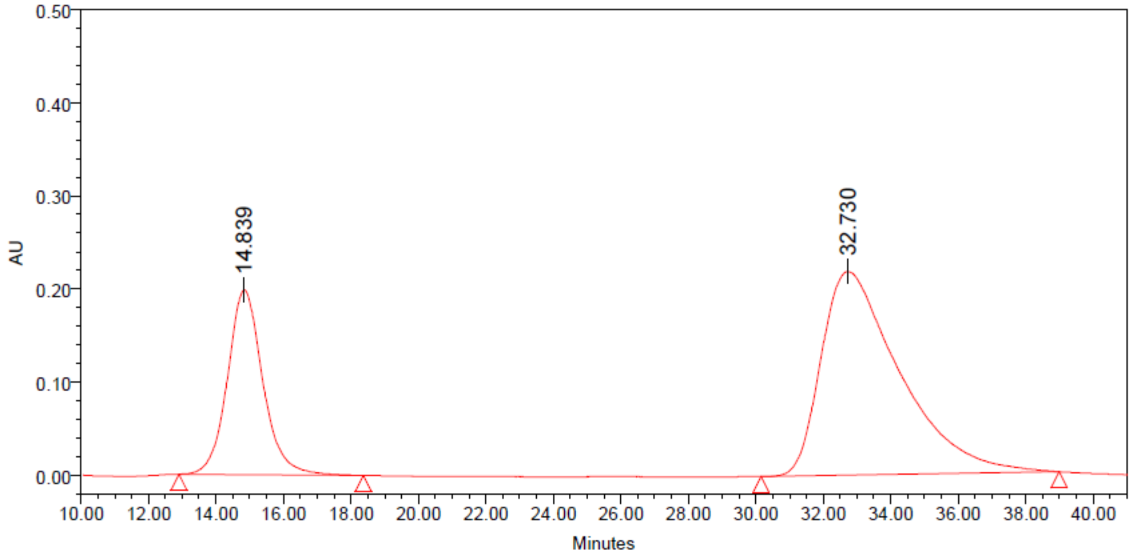

Processed Channel: PDA 253.2 nm

	Processed Channel	Retention Time (min)	Area	% Area	Height
1	PDA 253.2 nm	14.839	14454503	28.96	198394
2	PDA 253.2 nm	32.730	35454138	71.04	218546

Figure S33. Chiral sample of **dimethyl 6-amino-5-cyano-1-(4-methoxybenzamido)-4-phenyl-1,4-dihydropyridine-2,3-dicarboxylate (10fa)**.

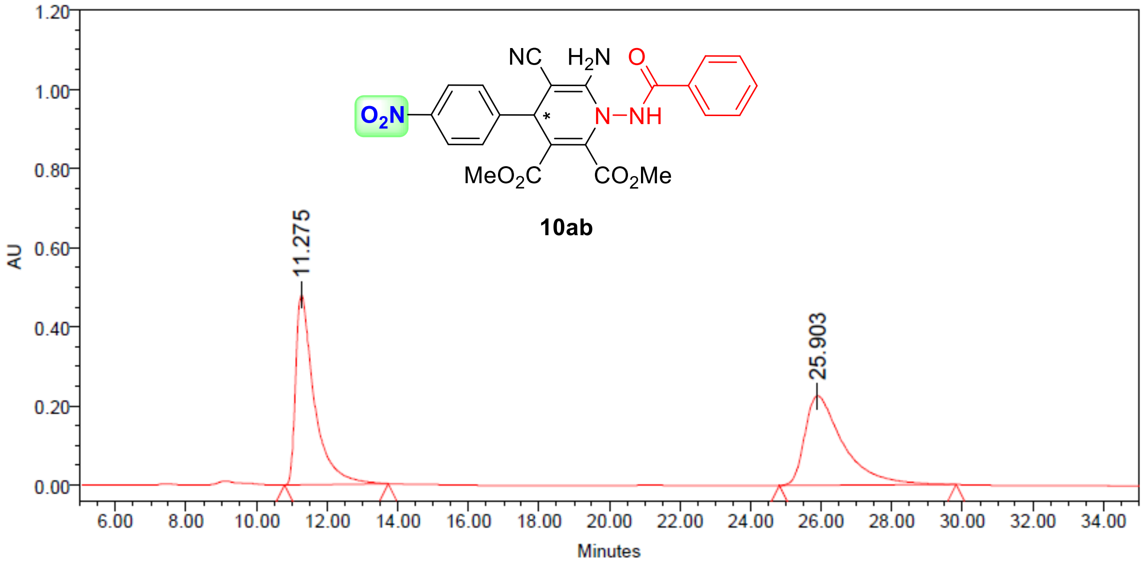

Processed Channel: PDA 236.6 nm

	Processed Channel	Retention Time (min)	Area	% Area	Height
1	PDA 236.6 nm	11.275	17622984	50.78	479710
2	PDA 236.6 nm	25.903	17082399	49.22	225531

Figure S34. Racemic mixture of **10ab**. Daicel Chiralpak IA column (*n*-hexane/*i*-PrOH = 70:30, flow rate 1 mL/min).

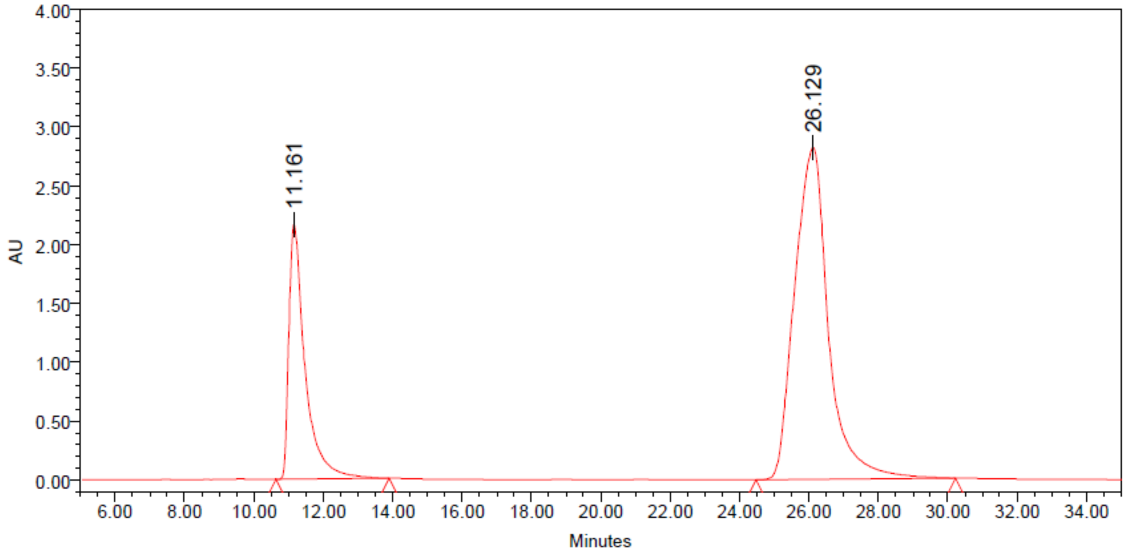

Processed Channel: PDA 236.6 nm

	Processed Channel	Retention Time (min)	Area	% Area	Height
1	PDA 236.6 nm	11.161	70343801	26.94	2168349
2	PDA 236.6 nm	26.129	190731169	73.06	2819267

Figure S35. Chiral sample of **dimethyl 6-amino-1-benzamido-5-cyano-4-(4-nitrophenyl)-1,4-dihydropyridine-2,3-dicarboxylate (10ab)**.

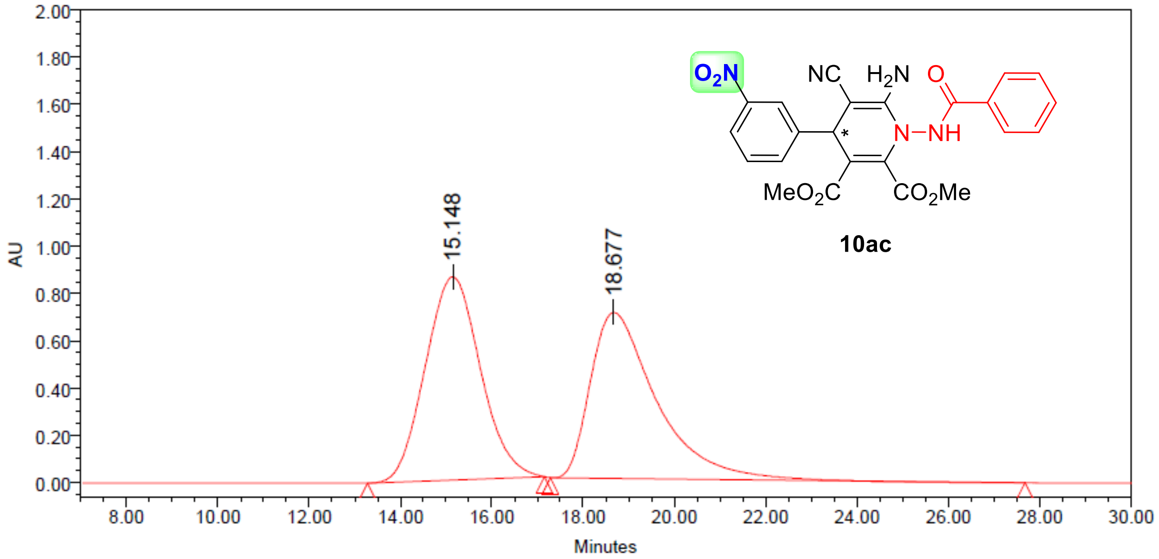

Processed Channel: PDA 238.9 nm

	Processed Channel	Retention Time (min)	Area	% Area	Height
1	PDA 238.9 nm	15.148	72022869	50.49	859290
2	PDA 238.9 nm	18.677	70638421	49.51	700873

Figure S36. Racemic mixture of **10ac**. Daicel Chiralpak IC column (*n*-hexane/*i*-PrOH = 70:30, flow rate 1 mL/min).

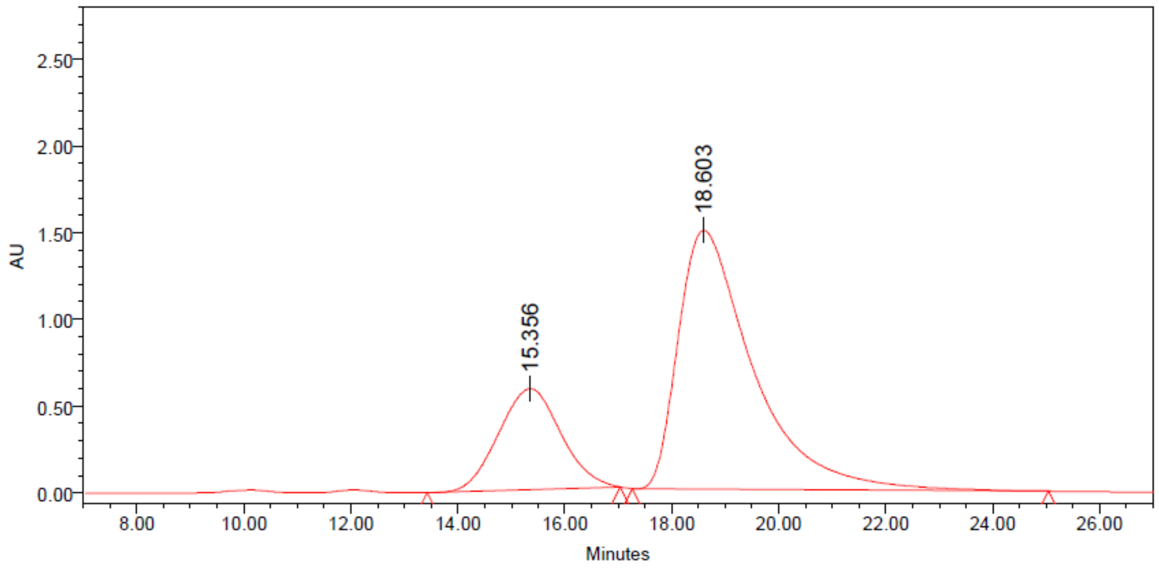

Processed Channel: PDA 238.9 nm

	Processed Channel	Retention Time (min)	Area	% Area	Height
1	PDA 238.9 nm	15.356	47060023	24.46	581731
2	PDA 238.9 nm	18.603	145335733	75.54	1491373

Figure S37. Chiral sample of **dimethyl 6-amino-1-benzamido-5-cyano-4-(3-nitrophenyl)-1,4-dihydropyridine-2,3-dicarboxylate (10ac)**.

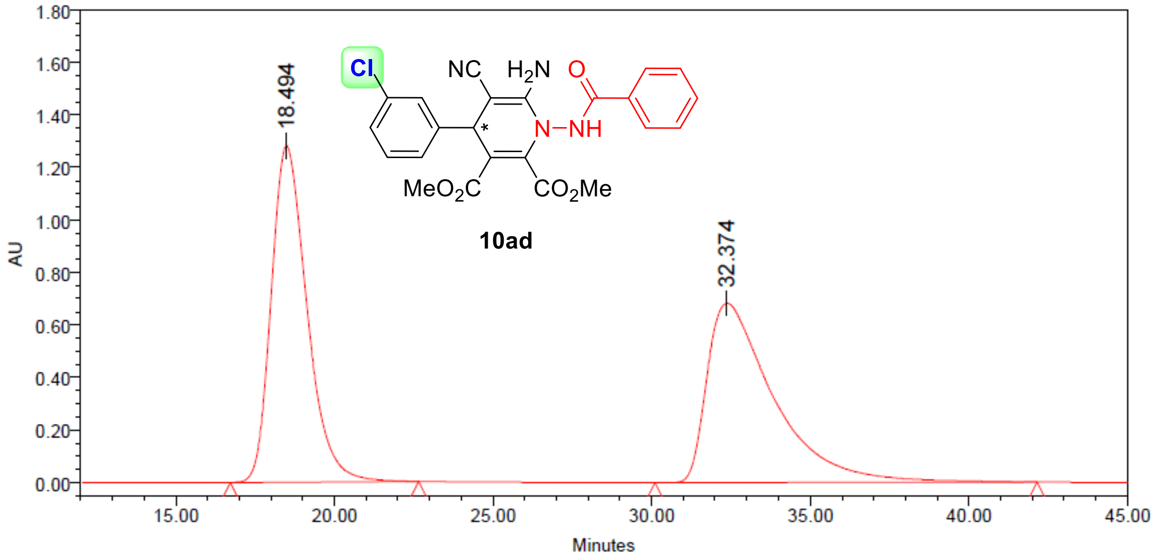

Processed Channel: PDA 236.6 nm

	Processed Channel	Retention Time (min)	Area	% Area	Height
1	PDA 236.6 nm	18.494	100441914	50.09	1281760
2	PDA 236.6 nm	32.374	100070753	49.91	682777

Figure S38. Racemic mixture of **10ad**. Daicel Chiralpak IC column (*n*-hexane/*i*-PrOH = 80:20, flow rate 1 mL/min).

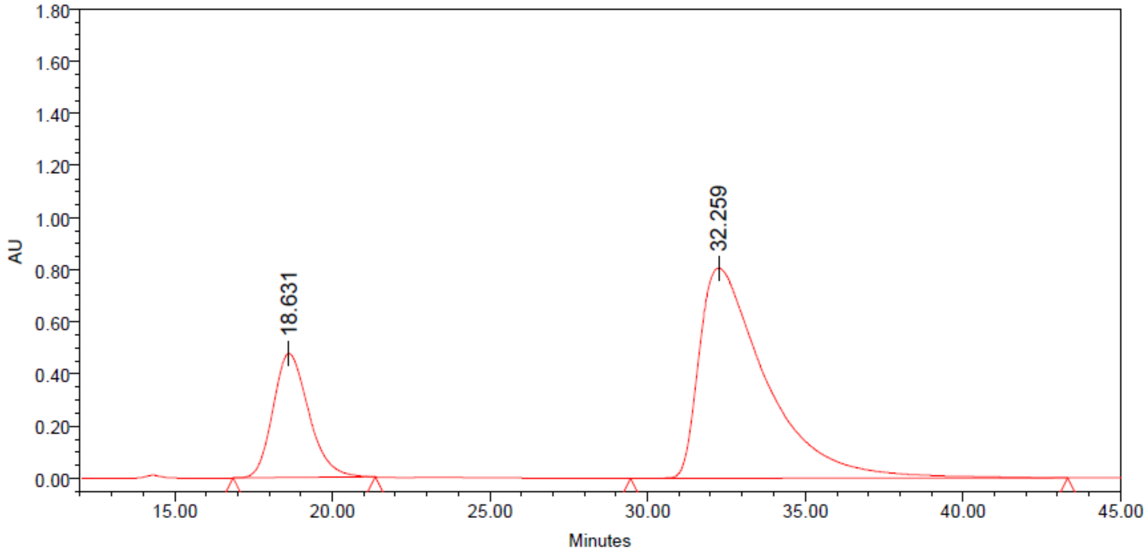

Processed Channel: PDA 236.6 nm

	Processed Channel	Retention Time (min)	Area	% Area	Height
1	PDA 236.6 nm	18.631	37654156	24.09	477002
2	PDA 236.6 nm	32.259	118683057	75.91	806171

Figure S39. Chiral sample of **dimethyl 6-amino-1-benzamido-4-(3-chlorophenyl)-5-cyano-1,4-dihydropyridine-2,3-dicarboxylate (10ad)**.

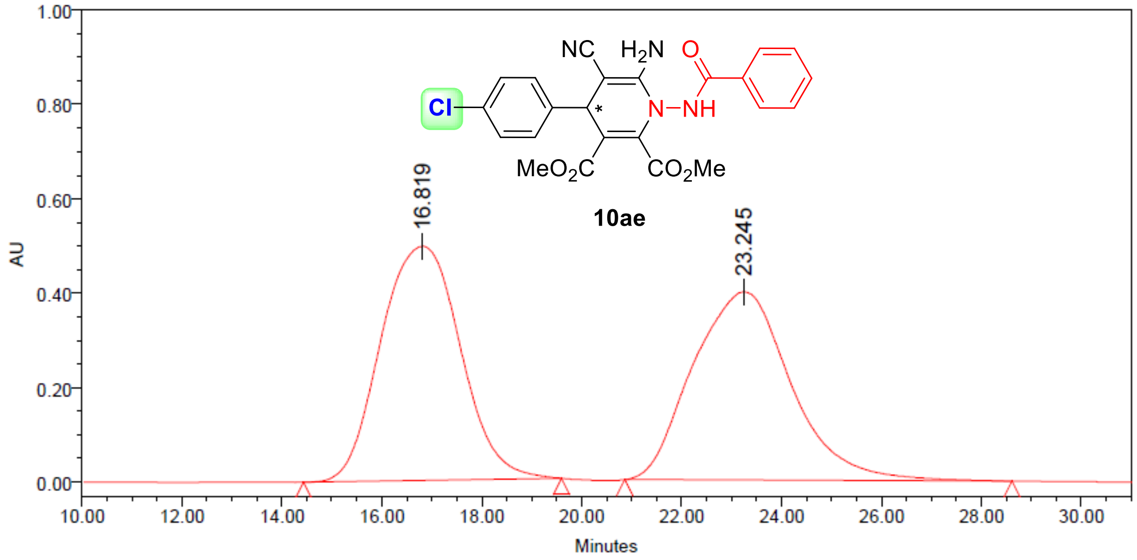

Processed Channel: PDA 237.7 nm

	Processed Channel	Retention Time (min)	Area	% Area	Height
1	PDA 237.7 nm	16.819	55049476	50.24	495723
2	PDA 237.7 nm	23.245	54529618	49.76	398603

Figure S40. Racemic mixture of **10ae**. Daicel Chiralpak IC column (*n*-hexane/*i*-PrOH = 80:20, flow rate 1 mL/min).

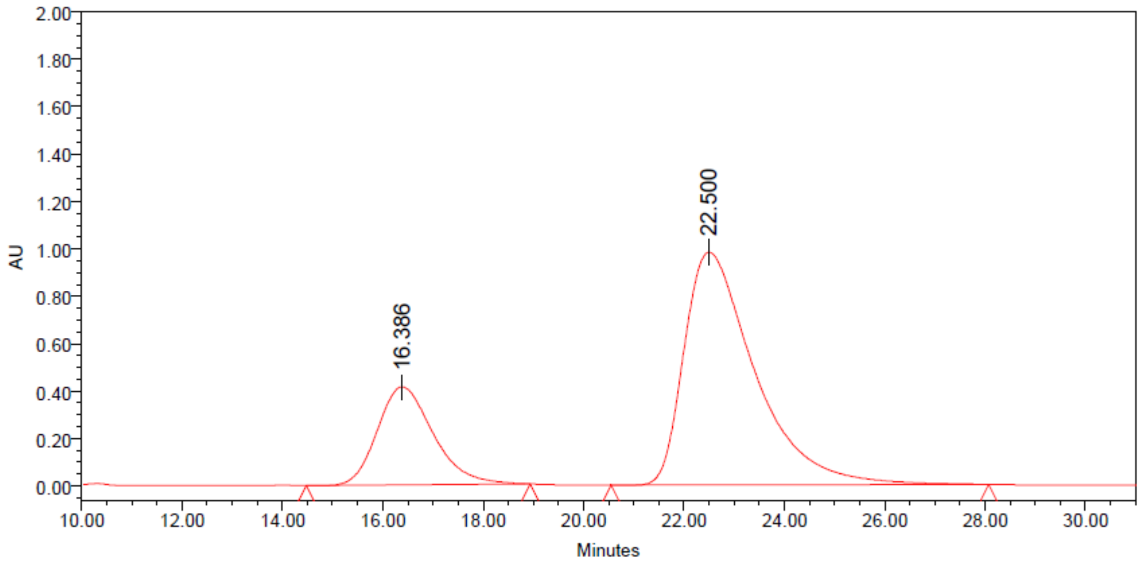

Processed Channel: PDA 237.7 nm

	Processed Channel	Retention Time (min)	Area	% Area	Height
1	PDA 237.7 nm	16.386	32047499	24.94	413395
2	PDA 237.7 nm	22.500	96450196	75.06	981830

Figure S41. Chiral sample of **dimethyl 6-amino-1-benzamido-4-(4-chlorophenyl)-5-cyano-1,4-dihydropyridine-2,3-dicarboxylate (10ae)**.

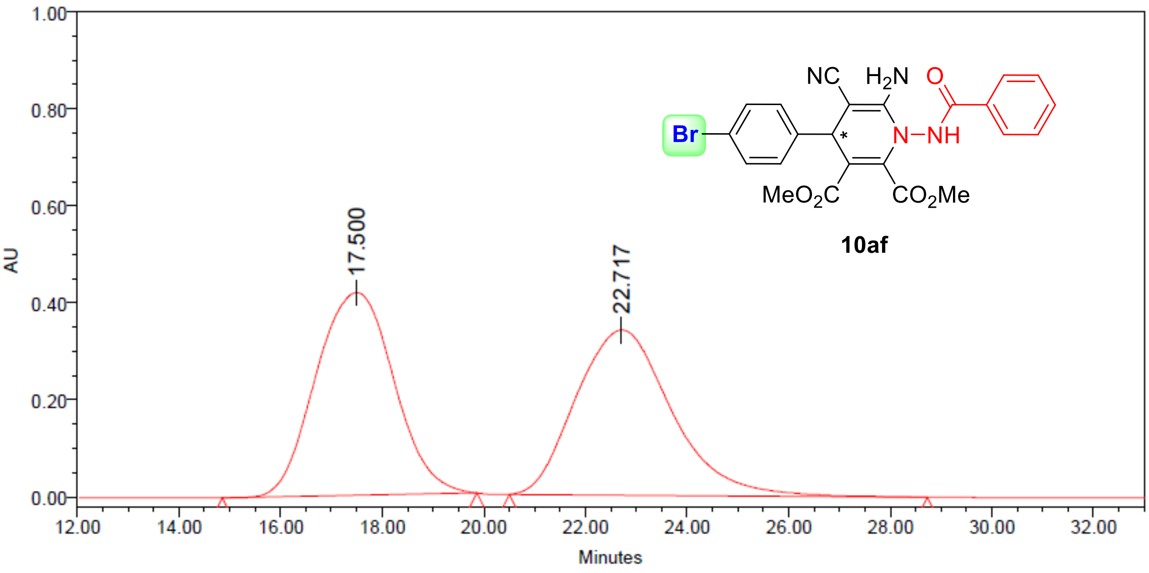

Processed Channel: PDA 236.6 nm

	Processed Channel	Retention Time (min)	Area	% Area	Height
1	PDA 236.6 nm	17.500	44708714	50.06	417705
2	PDA 236.6 nm	22.717	44606766	49.94	340507

Figure S42. Racemic mixture of **10af**. Daicel Chiralpak IC column (*n*-hexane/*i*-PrOH = 80:20, flow rate 1 mL/min).

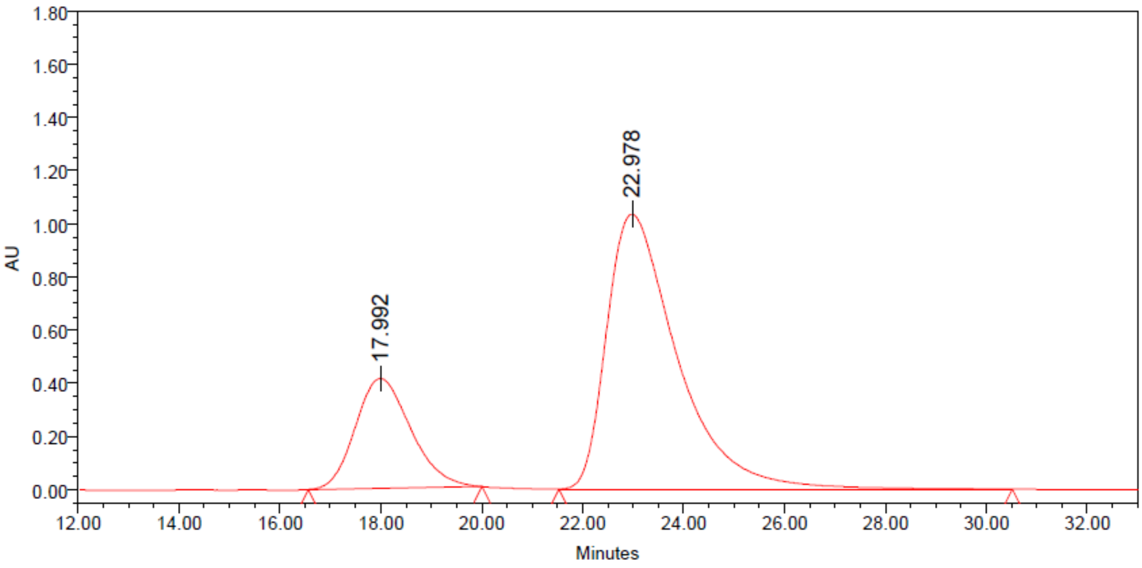

Processed Channel: PDA 236.6 nm

	Processed Channel	Retention Time (min)	Area	% Area	Height
1	PDA 236.6 nm	17.992	31599134	23.88	413822
2	PDA 236.6 nm	22.978	100733240	76.12	1035302

Figure S43. Chiral sample of **dimethyl 6-amino-1-benzamido-4-(4-bromophenyl)-5-cyano-1,4-dihydropyridine-2,3-dicarboxylate (10af)**.

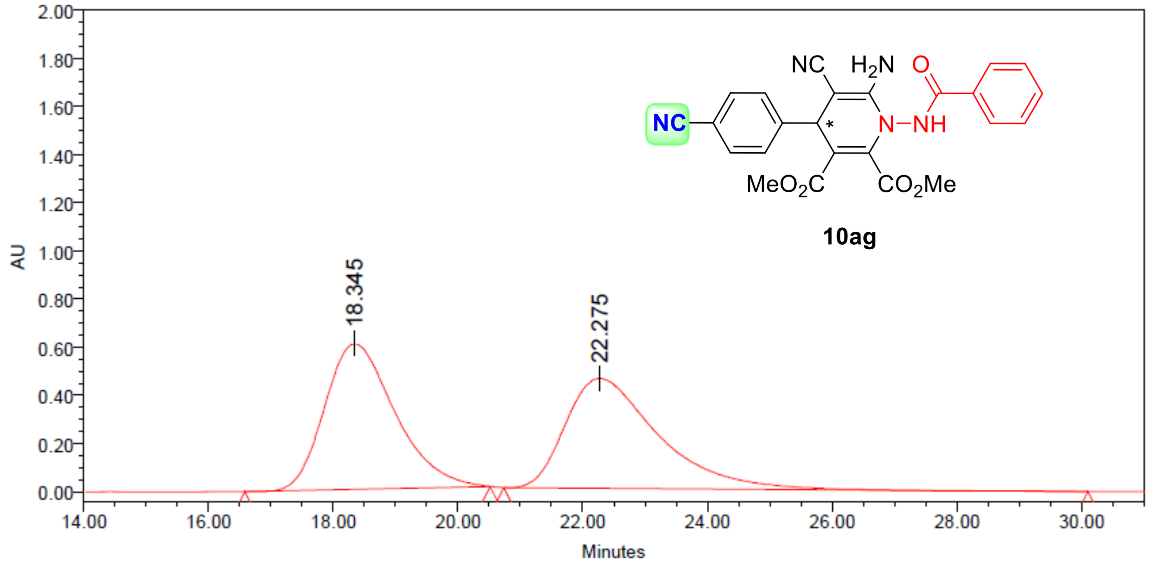

Processed Channel: PDA 234.2 nm

	Processed Channel	Retention Time (min)	Area	% Area	Height
1	PDA 234.2 nm	18.345	47416686	50.17	604355
2	PDA 234.2 nm	22.275	47099216	49.83	455713

Figure S44. Racemic mixture of **10ag**. Daicel Chiralpak IC column (*n*-hexane/*i*-PrOH = 70:30, flow rate 1 mL/min).

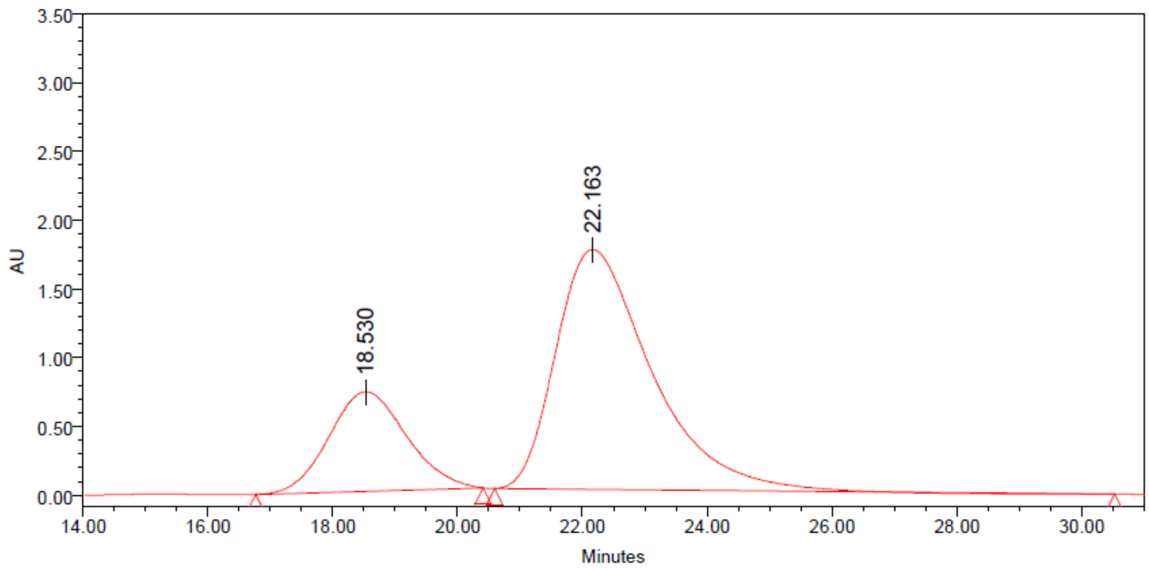

Processed Channel: PDA 234.2 nm

	Processed Channel	Retention Time (min)	Area	% Area	Height
1	PDA 234.2 nm	18.530	62541011	25.05	724498
2	PDA 234.2 nm	22.163	187103255	74.95	1746223

Figure S45. Chiral sample of **dimethyl 6-amino-1-benzamido-5-cyano-4-(4-cyanophenyl)-1,4-dihydropyridine-2,3-dicarboxylate (10ag)**.

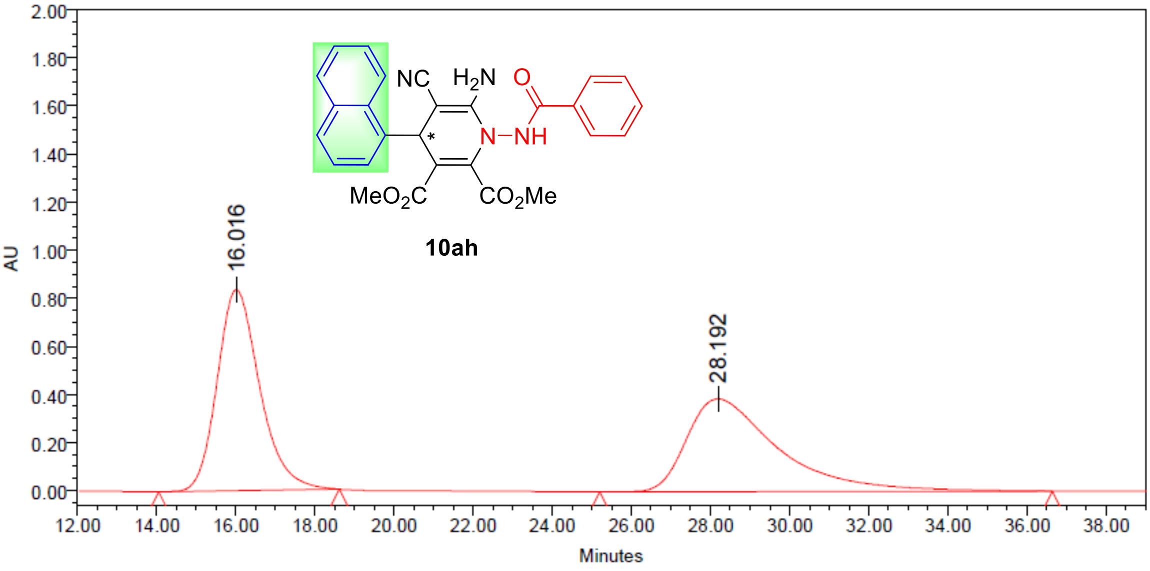

Processed Channel: PDA 221.2 nm

	Processed Channel	Retention Time (min)	Area	% Area	Height
1	PDA 221.2 nm	16.016	62495350	50.32	836041
2	PDA 221.2 nm	28.192	61702057	49.68	385566

Figure S46. Racemic mixture of **10ah**. Daicel Chiralpak IC column (*n*-hexane/*i*-PrOH = 70:30, flow rate 1 mL/min).

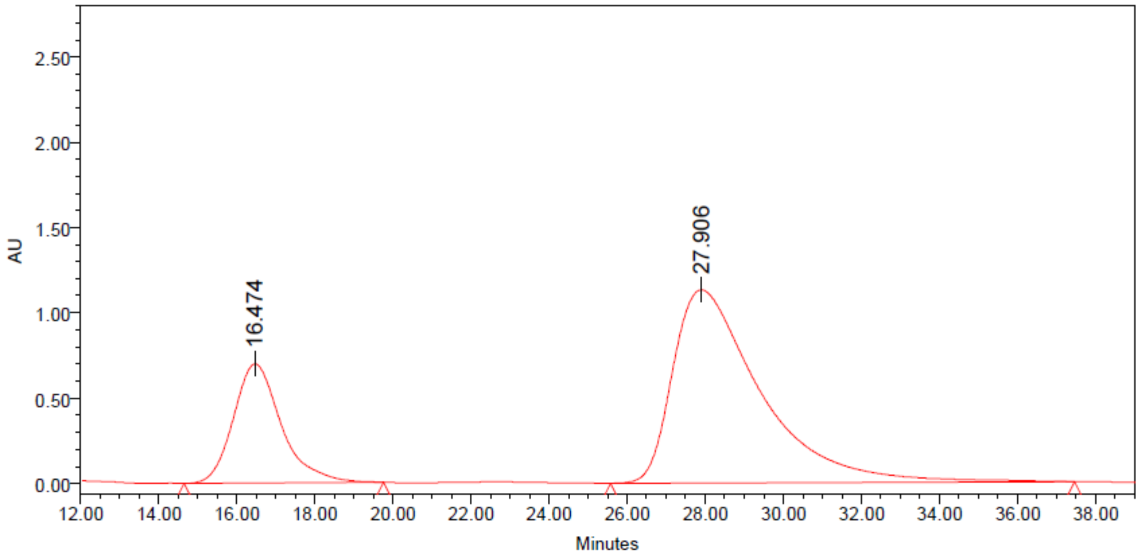

Processed Channel: PDA 221.2 nm

	Processed Channel	Retention Time (min)	Area	% Area	Height
1	PDA 221.2 nm	16.474	59358674	25.17	697447
2	PDA 221.2 nm	27.906	176513601	74.83	1130499

Figure S47. Chiral sample of **dimethyl 6-amino-1-benzamido-5-cyano-4-(naphthalen-1-yl)-1,4-dihydropyridine-2,3-dicarboxylate (10ah)**.

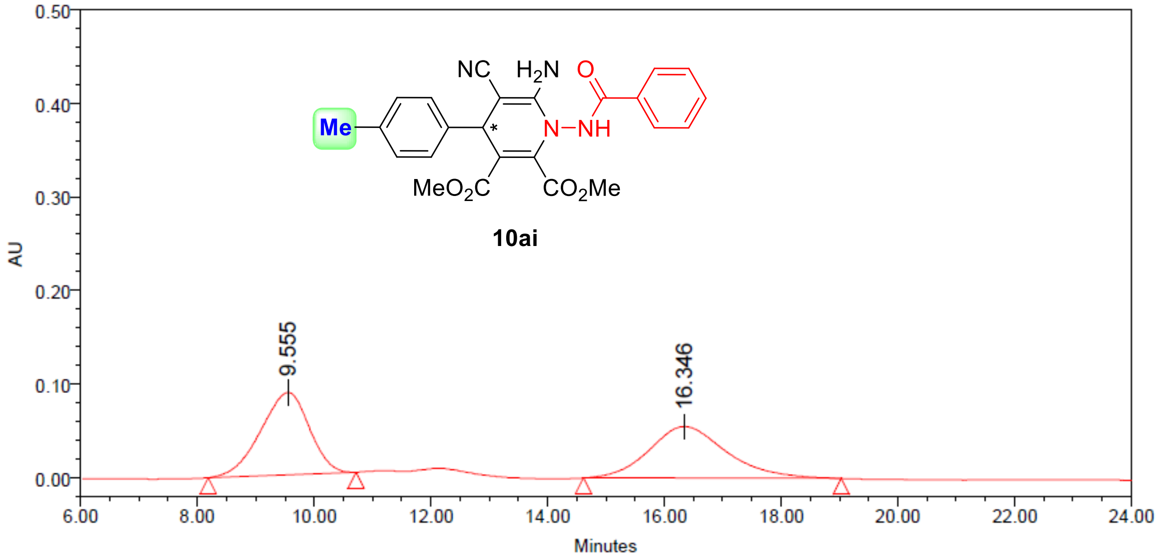

Processed Channel: PDA 237.7 nm

	Processed Channel	Retention Time (min)	Area	% Area	Height
1	PDA 237.7 nm	9.555	5079543	49.91	88016
2	PDA 237.7 nm	16.346	5098577	50.09	55184

Figure S48. Racemic mixture of **10ai**. Daicel Chiralpak IC column (*n*-hexane/*i*-PrOH = 70:30, flow rate 1 mL/min).

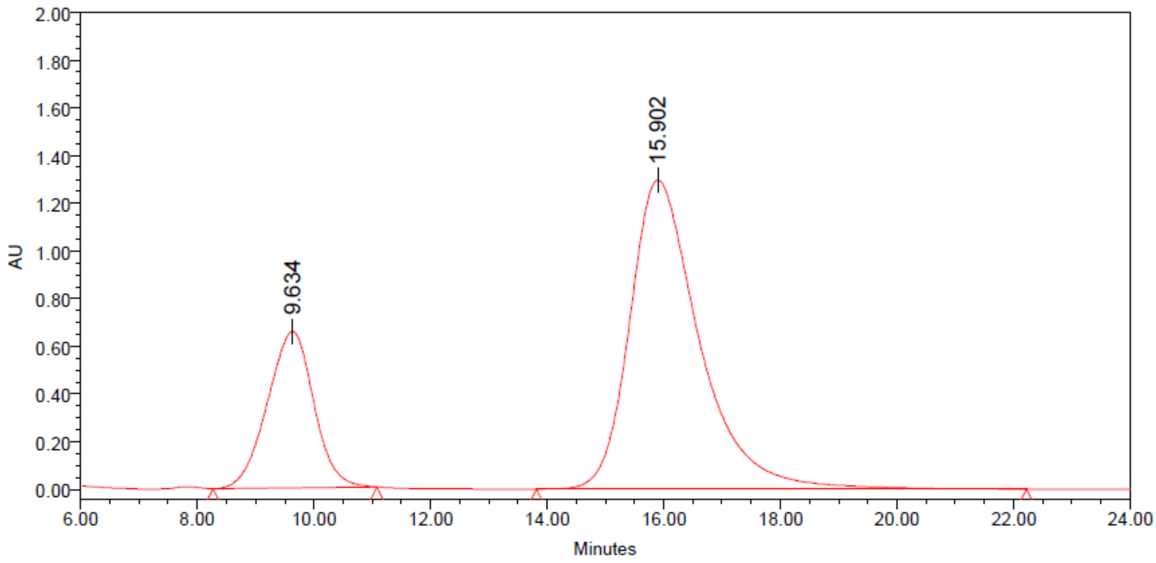

Processed Channel: PDA 237.7 nm

	Processed Channel	Retention Time (min)	Area	% Area	Height
1	PDA 237.7 nm	9.634	36213748	25.17	655692
2	PDA 237.7 nm	15.902	107660471	74.83	1295935

Figure S49. Chiral sample of **dimethyl 6-amino-1-benzamido-5-cyano-4-(p-tolyl)-1,4-dihydropyridine-2,3-dicarboxylate (10ai)**.

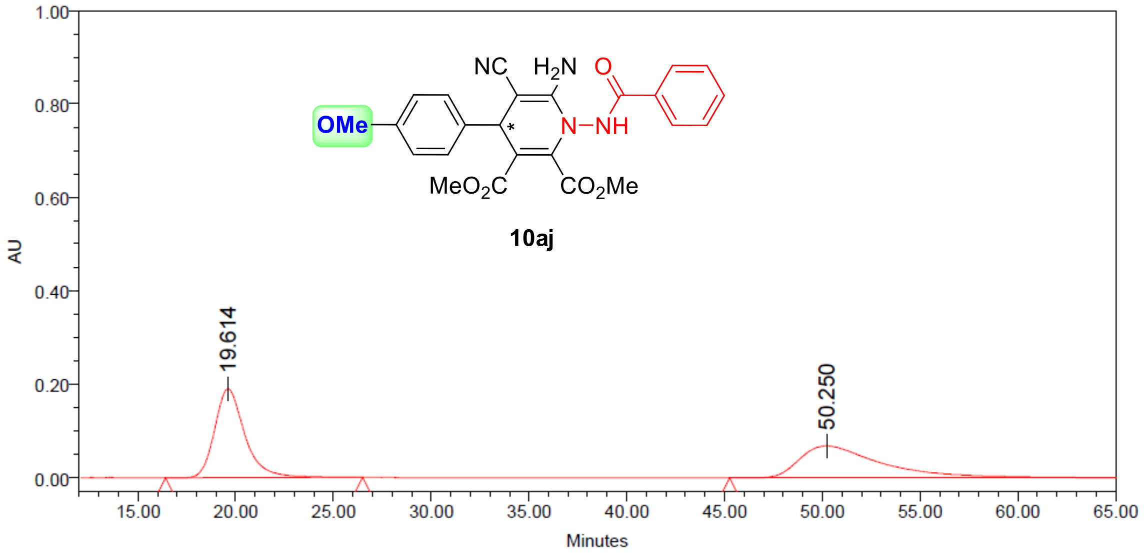

Processed Channel: PDA 247.4 nm

	Processed Channel	Retention Time (min)	Area	% Area	Height
1	PDA 247.4 nm	19.614	20322514	50.29	190004
2	PDA 247.4 nm	50.250	20087936	49.71	68499

Figure S50. Racemic mixture of **10aj**. Daicel Chiralpak IC column (*n*-hexane/*i*-PrOH = 70:30, flow rate 1 mL/min).

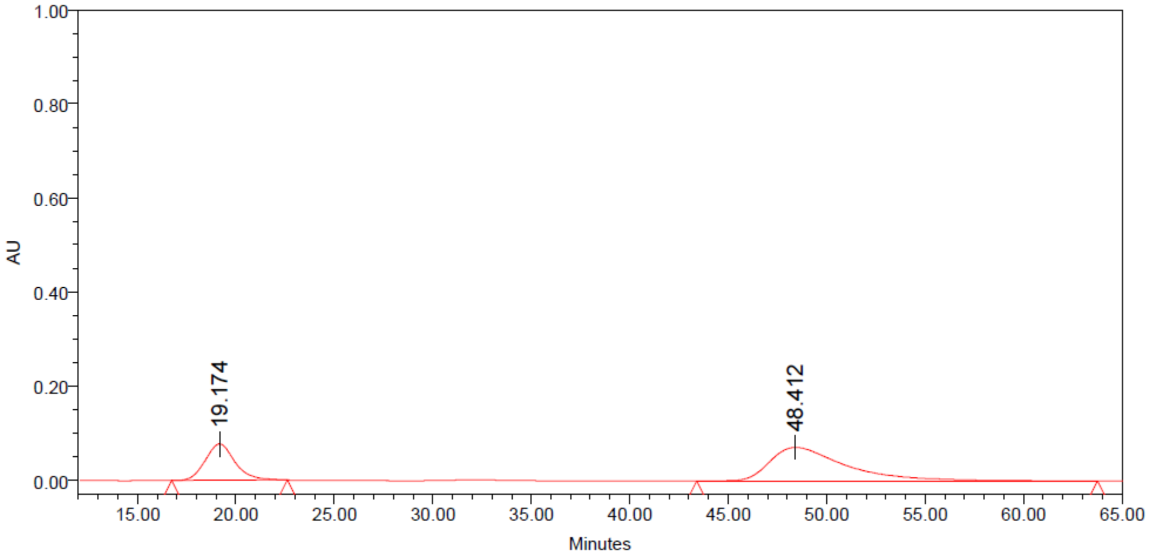

Processed Channel: PDA 237.7 nm

	Processed Channel	Retention Time (min)	Area	% Area	Height
1	PDA 237.7 nm	19.174	8146189	29.96	77509
2	PDA 237.7 nm	48.412	19046679	70.04	71318

Figure S51. Chiral sample of **dimethyl 6-amino-1-benzamido-5-cyano-4-(4-methoxyphenyl)-1,4-dihydropyridine-2,3-dicarboxylate (10aj)**.

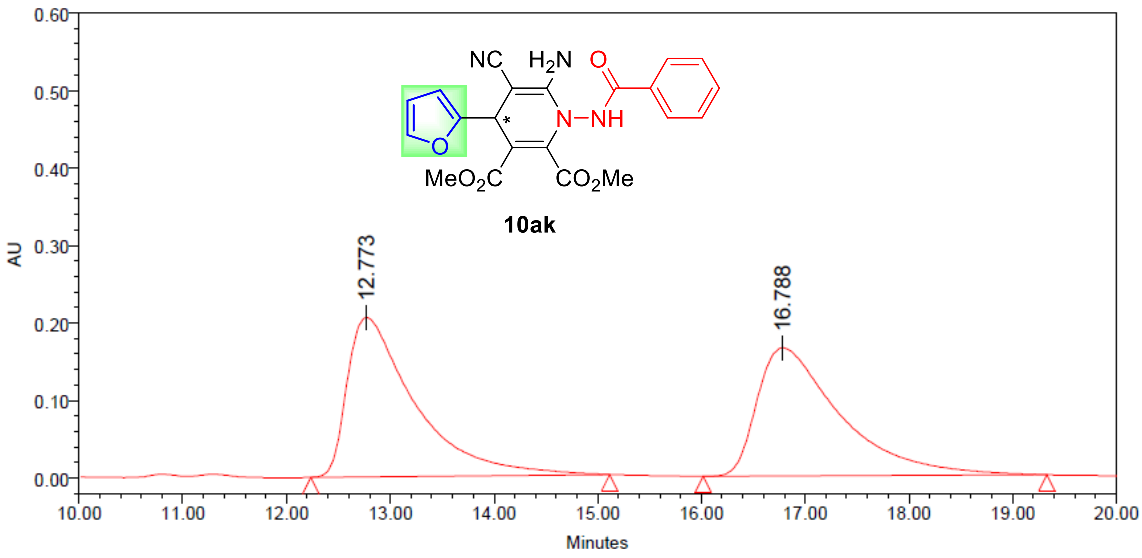

Processed Channel: PDA 236.6 nm

	Processed Channel	Retention Time (min)	Area	% Area	Height
1	PDA 236.6 nm	12.773	9337925	50.31	205692
2	PDA 236.6 nm	16.788	9222028	49.69	165429

Figure S52. Racemic mixture of **10ak**. Daicel Chiralpak IA column (*n*-hexane/*i*-PrOH = 80:20, flow rate 1 mL/min).

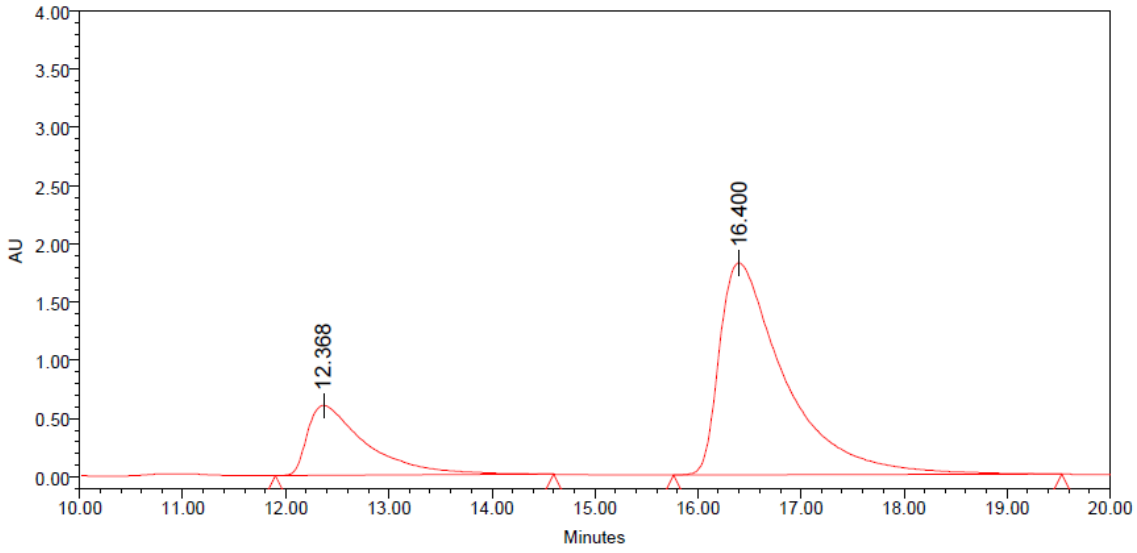

Processed Channel: PDA 236.6 nm

	Processed Channel	Retention Time (min)	Area	% Area	Height
1	PDA 236.6 nm	12.368	23993346	22.92	601900
2	PDA 236.6 nm	16.400	80675819	77.08	1822151

Figure S53. Chiral sample of **dimethyl 6-amino-1-benzamido-5-cyano-4-(furan-2-yl)-1,4-dihydropyridine-2,3-dicarboxylate (10ak)**.

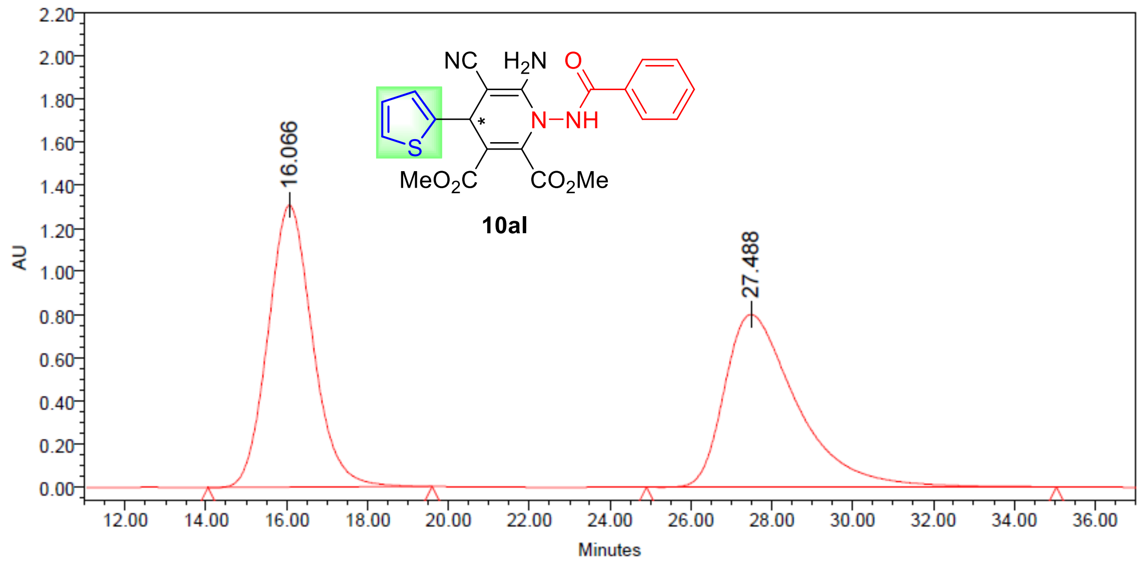

Processed Channel: PDA 236.6 nm

	Processed Channel	Retention Time (min)	Area	% Area	Height
1	PDA 236.6 nm	16.066	100596140	50.23	1308641
2	PDA 236.6 nm	27.488	99663464	49.77	800792

Figure S54. Racemic mixture of **10al**. Daicel Chiralpak IC column (*n*-hexane/*i*-PrOH = 70:30, flow rate 1 mL/min).

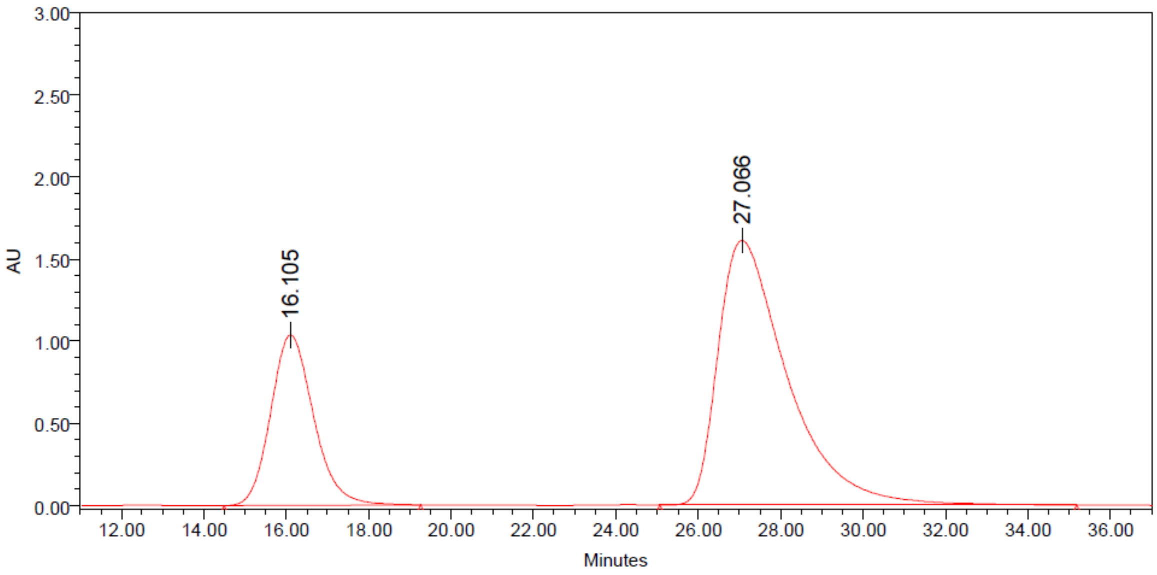

Processed Channel: PDA 236.6 nm

	Processed Channel	Retention Time (min)	Area	% Area	Height
1	PDA 236.6 nm	16.105	74932650	28.49	1035798
2	PDA 236.6 nm	27.066	188049038	71.51	1607532

Figure S55. Chiral sample of **dimethyl 6-amino-1-benzamido-5-cyano-4-(thiophen-2-yl)-1,4-dihydropyridine-2,3-dicarboxylate (10al)**.

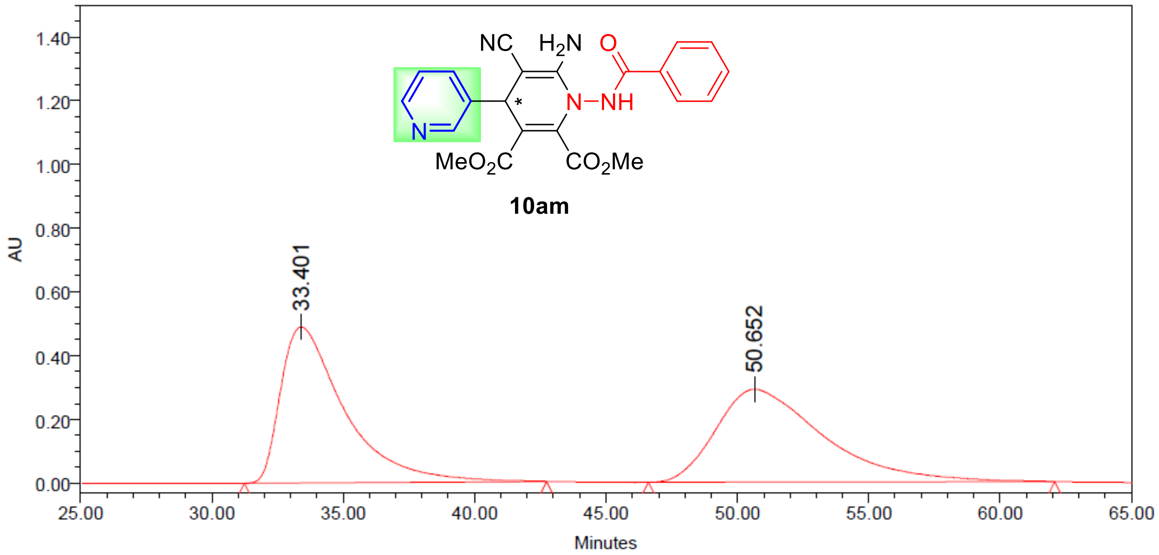

Processed Channel: PDA 237.7 nm

	Processed Channel	Retention Time (min)	Area	% Area	Height
1	PDA 237.7 nm	33.401	82969649	49.99	489103
2	PDA 237.7 nm	50.652	83002489	50.01	291330

Figure S56. Racemic mixture of **10am**. Daicel Chiralpak IC column (*n*-hexane/*i*-PrOH = 70:30, flow rate 1 mL/min).

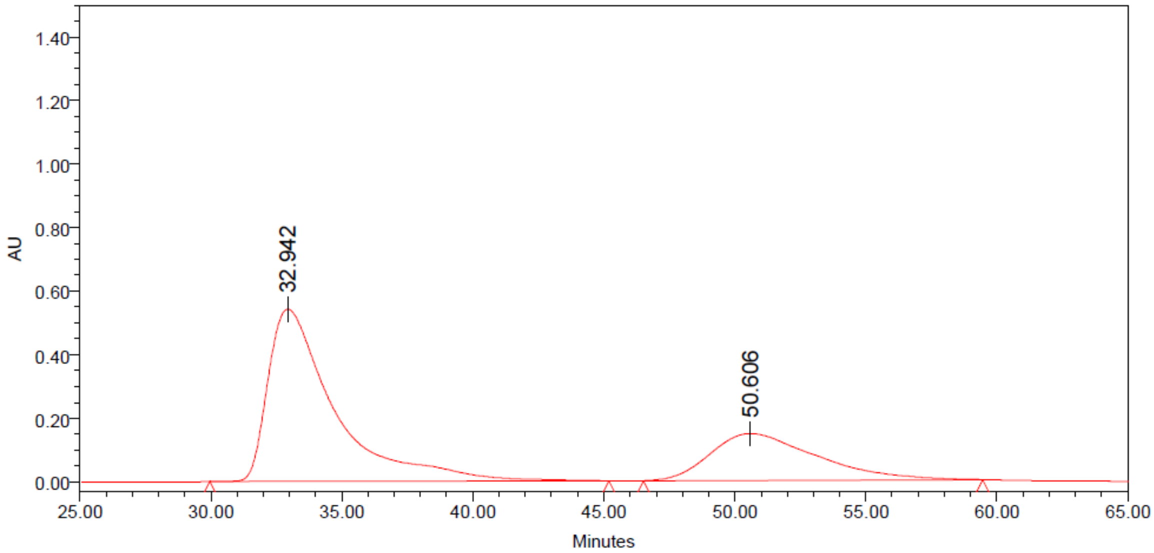

Processed Channel: PDA 237.7 nm

	Processed Channel	Retention Time (min)	Area	% Area	Height
1	PDA 237.7 nm	32.942	95793294	69.83	542245
2	PDA 237.7 nm	50.606	41390261	30.17	146845

Figure S57. Chiral sample of **dimethyl 6'-amino-1'-benzamido-5'-cyano-1',4'-dihydro-[3,4'-bipyridine]-2',3'-dicarboxylate (10am)**.
